# Design, Synthesis, In Vitro Biological Activity Evaluation and Stabilized Nanostructured Lipid Carrier Formulation of Newly Synthesized Schiff Bases-Based TMP Moieties

**DOI:** 10.3390/ph15060679

**Published:** 2022-05-28

**Authors:** Syed Nasir Abbas Bukhari, Mohamed Y. Zakaria, Muhammad Usman Munir, Naveed Ahmad, Mervat A Elsherif, Rasha Emad Badr, Ahmad Khalaf Hassan, Ali H. Abu Almaaty, Islam Zaki

**Affiliations:** 1 Department of Pharmaceutical Chemistry, College of Pharmacy, Jouf University, Sakaka 72388, Saudi Arabia; mumunir@ju.edu.sa; 2 Department of Pharmaceutics and Industrial Pharmacy, Faculty of Pharmacy, Port Said University, Port Said 42526, Egypt; dr_m_yehia@live.com; 3 Department of Pharmaceutics, College of Pharmacy, Jouf University, Sakaka 72388, Saudi Arabia; nakahmad@ju.edu.sa; 4 Chemistry Department, College of Science, Jouf University, Sakaka 72388, Saudi Arabia; maelsherif@ju.edu.sa; 5 Clinical and Chemical Pathology Department, Faculty of Medicine, Port Said University, Port Said 42526, Egypt; dr.rashaemad@hotmail.com; 6 Zoology Department, Faculty of Science, Port Said University, Port Said 42526, Egypt; ahkhalaf71@yahoo.com (A.K.H.); ali_zoology_2010@yahoo.com (A.H.A.A.); 7 Pharmaceutical Organic Chemistry Department, Faculty of Pharmacy, Port Said University, Port Said 42526, Egypt

**Keywords:** TMP, Schiff base, synthesis, cytotoxicity, tubulin, cell cycle analysis, annexin V, p53, Bax, Bcl-2

## Abstract

A series of novel Schiff bases-based TMP moieties have been designed and synthesized as potential anticancer agents. The target Schiff bases were screened for their cytotoxic activity against the MDA-MB-231 breast cancer cell line. Most of the tested molecules revealed good cytotoxic activity, especially compounds **4h,** **4j** and **5d**. Being the most potent, compound **4h** showed good tubulin polymerization inhibition activity as revealed by immunofluorescence analysis and ELISA assay. Additionally, compound **4h** was screened for cell cycle disturbance and apoptosis induction. Pre-G1 apoptosis and cell growth halt at the G2/M phase were discovered to be caused by it. Moreover, compound **4h** induced apoptosis via p53 and Bax activation, as well as reduced the level of Bcl-2. Additionally, the most potent compound **4h** was lodged on nanostructured lipid carriers (NLCs). 2^3^ full factorial design was involved to govern the influence of the fabrication variables on the in vitro characters of the casted NLCs. F3 was picked as the optimum formula exhibiting dominant desirability value 0.805, EE% 95.6 ± 2.4, PS 222.4 ±18.7, PDI 0.23 ± 0.05 and ZP −39.2 ± 3.9 Mv. Furthermore, F3 affirmed improved solubility and release over the drug suspension. In the comparative cytotoxic activity, F3 was capable of diminishing the IC_50_ by around 2.15 times for pure **4h**, while nearly close to the IC_50_ of the reference drug. Thus, NLCs could be a potential platform for boosted antitumor activity.

## 1. Introduction

**Figure 1:**
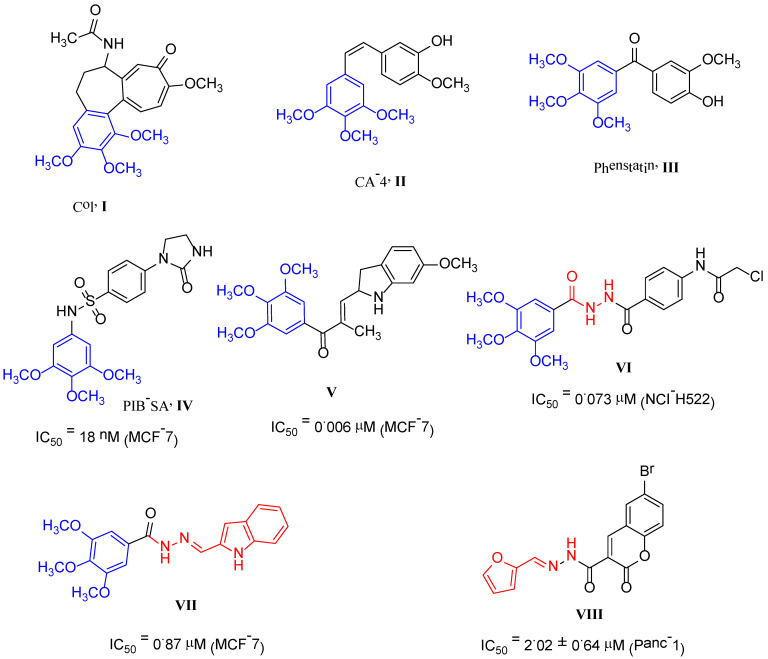
Chemical structure of reported tubulin polymerization inhibitors containing TMP moiety (**I**–**VI**) and reported Schiff base derivatives (**VII**–**VIII**) possessing anticancer activity.

Microtubules are among the most important molecular targets for cancer chemotherapeutic treatment [1,2,3]. The formation of microtubules is a dynamic process that involves the assembly and disassembly of α and β-tubulin subunits [4,5,6]. A chemotherapeutic agent binds to tubulin and interferes with this dynamic stability, and thus induces cell cycle disturbance, resulting in apoptosis induction and cell death [7,8,9]. Compounds containing trimethoxyphenyl (TMP) ring have been reported to have excellent potent cytotoxic activity against a broad range of cancer cell lines through inhibition of α and β-tubulin dynamic equilibrium [10,11,12,13]. The creation of tubulin polymerization inhibitors has been the subject of several investigations in recent years. As a result, anticancer medicines with tubulin polymerization inhibitory activity have been designed and developed using TMP-containing compounds. [14,15,16]. Colchicine (Col) **I**, combretastatin A-4 (CA-4) **II** and phenstatin **III** are natural compounds that bind to the colchicine binding site and prevent the polymerization of tubulins to form microtubules [17,18,19]. In addition, PIB-SA **IV** efficiently inhibited tumor proliferation similar to that of CA-4 [20]. Moreover, compound **V** showed strong antiproliferative activity against four human cancer cell lines with IC_50_ values of 3–9 nm, as well as high selectivity to normal cells [21]. Additionally, the *bis*-hydrazide molecule **VI** displayed 83.1% in vitro tubulin polymerization inhibition activity at its IC_50_ dose level [22] (Figure 1).

A literature survey revealed that Schiff base derivatives are known for their various biological activities [23,24,25]. In addition, many Schiff bases exhibited promising antitumor potency against different cancer cell lines [26,27,28]. The Schiff base possessing TMP ring **VII** showed antiproliferative activity against the MCF-7 breast cancer cell line with an IC_50_ value of 0.87 μM [26]. Furthermore, Schiff base **VIII** possessing *N*-furylmethylidene moiety was identified as a potent antiproliferative agent against many cancer cell lines [28].

**Figure 2:**
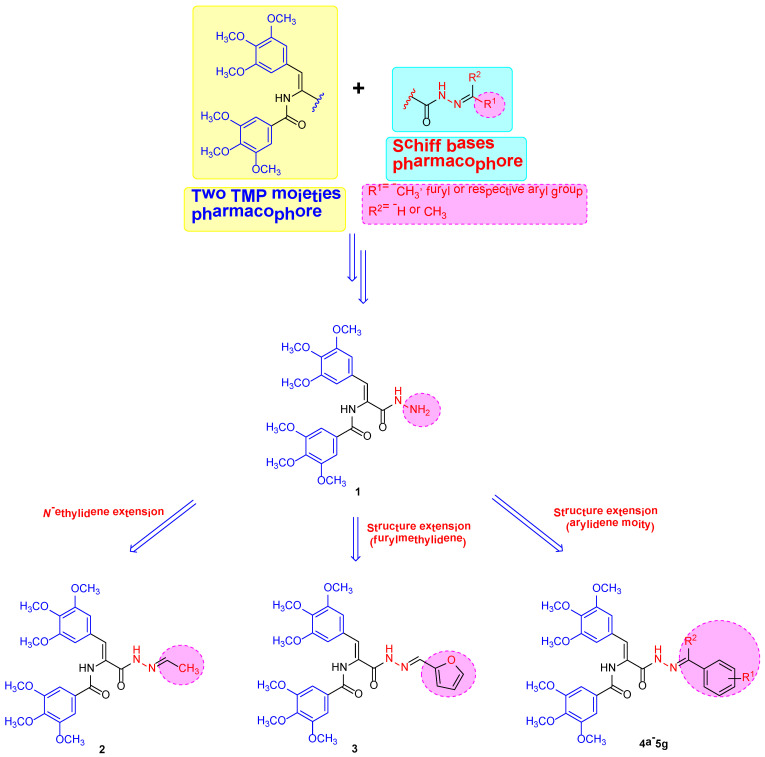
Design strategy of the prepared compounds **2–5g**.

On the basis of the aforementioned structural analysis, as well as in keeping with our general enthusiasm for the design and development of novel anticancer agents, [29,30,31,32], as potential anticancer medicines, the current work focused on the design and synthesis of novel Schiff bases-based TMP moieties. The basic structural skeleton of the lead compound is formed from two TMP rings connected through amide-acrylic acid hydrazide groups. The target molecules were intended to have different aliphatic and aromatic side chains at the hydrazide group to study the impact of these structural variations on antiproliferative activity. The hydrazide group in the lead compound was subjected to the replacement of the amino (NH_2_) group to give *N*-ethylidene hydrazinyl derivative **2**. In addition, further structure extension of hydrazide group with furan or substituted phenyl moieties afforded *N*-furylmethylidene hydrazinyl derivative **3** or *N*-arylidene hydrazinyl molecules **4a**–**5g**, respectively (Figure 2). The MDA-MB-231 cell line was used to test the cytotoxic activity of the target compounds. Additional studies were conducted on the most potent compound, **4h**, including DNA flow cytometry and tubulin polymerization inhibition, as well as apoptotic-related tests. In addition, the most potent and promising drug was incorporated in nanostructured lipid carriers (NLCs) as an attempt to improve its solubility and other kinetic parameters, thereby boosting its cytotoxicity. This was attained by the formulation of 8 NLCs formulae of different compositions obtained from 2^3^ full factorial design and the impact of the formulation variables on the EE%, PS and ZP was analyzed by Stat-Ease^®^ V.13 software (Design-Expert TM; Minneapolis, MN, USA). Then, the optimum formula was selected and involved in further characterization especially the comparative cytotoxicity study relative to the pure drug to demonstrate the influence of the NLCs of IC_50_ on the compound.

## 2. Results and Discussion

### 2.1. Chemistry

**Scheme 1:**
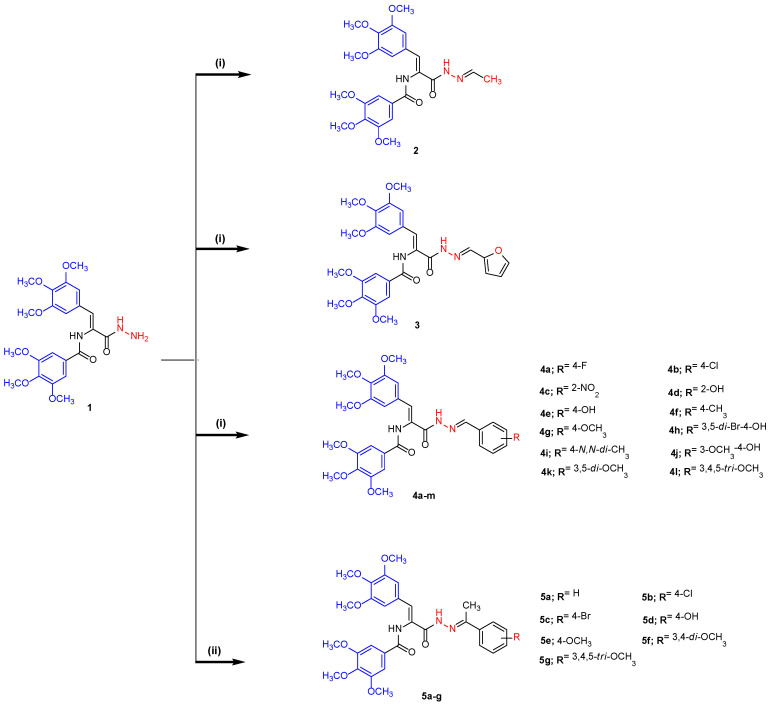
Synthesis of the target compounds **2**–**5g**. Reagent and reaction condition: (**i**) acetaldehyde, furfural or respective aryl aldehyde, EtOH, AcOH, reflux 6–8 h; (**ii**) respective aryl ketone, n-butanol, reflux 6–8 h.

The synthesis of target Schiff bases-based TMP compounds is outlined in Scheme 1. The reaction of hydrazide 1 with acetaldehyde, furfural or different aromatic aldehyde in absolute ethanol in the presence of catalytic amounts of glacial acetic acid produced the corresponding *N*-(3-((*Z*)-2-Ethylidenehydrazinyl)-3-oxo-1-(3,4,5-trimethoxyphenyl)prop-1-en-2-yl)-3,4,5-trimethoxybenzamide (**2**), *N*-(3-((*E*)-2-(Furan-2-ylmethylene)hydrazinyl)-3-oxo-1-(3,4,5-trimethoxyphenyl)prop-1-en-2-yl)-3,4,5-trimethoxybenzamide (**3**) or *N*-(3-((*E*)-2-(Aryl)hydrazinyl)-3-oxo-1-(3,4,5-trimethoxyphenyl)prop-1-en-2-yl)-3,4,5-trimethoxybenzamides **4a**–**l**, respectively. In the same manner, condensing the hydrazide **1** with convenient aryl ketone in n-butanol furnished the corresponding *N*-(3-((*E*)-2-(Arylethylidene)hydrazinyl)-3-oxo-1-(3,4,5-trimethoxyphenyl)prop-1-en-2-yl)-3,4,5-trimethoxybenzamides **5a**–**g**, respectively. The structure of the prepared Schiff bases was confirmed by elemental analysis and spectral data (^1^H-NMR and ^13^C-NMR spectra) and were consistent with the proposed structures. ^1^H-NMR spectra of compound **2** showed the presence of four proton peaks; at δ 10.00 and 9.89 ppm attributed to two amidic (NH) protons, at δ 9.85 ppm due to proton attached to imine carbon and at δ 1.89 ppm related to methyl protons attached to imine carbon (CH_3_C=N). ^13^C-NMR spectra of **2** showed the presence of two characteristic carbon signals; at δ 164.60 ppm corresponding to (C=N) carbon and at δ 21.08 ppm related to methyl carbon (CH_3_C=N). In addition, ^1^H-NMR spectra of compounds **3** and **4a**–**l** showed three singlet signals at δ 11.32–12.02 and 10.00–10.10 ppm related to two amidic (NH) protons and at δ 8.29–8.85 ppm corresponding to characteristic azomethine (N=CH) proton. In addition, ^1^H-NMR spectra of compounds **3** and **4a**–**l** showed additional signals at the aromatic region corresponding to the presence of furan or substituted phenyl moiety. ^13^C-NMR spectra of compounds **3** and **4a**–**l** showed a characteristic signal at δ 144.84–148.83 ppm related to azomethine (N=CH) carbon and two signals at δ 165.66–165.75 and 162.02–163.18 ppm related to two amide groups. Additionally, the formation of Schiff bases **5a**–**g** was confirmed by ^1^H-NMR and ^13^C-NMR spectral studies. ^1^H-NMR revealed the presence of three peaks at δ 10.43–10.63 and 10.05–10.10 ppm corresponding to two amidic (NH) protons and at δ 2.23–2.31 ppm related to methyl group attached to imine carbon. ^13^C-NMR spectra of **5a**–**g** confirmed the carbon skeleton due to the presence of two signals at δ 145.73–149.84 ppm related to imine (C=N) carbon and at δ 14.82–15.33 ppm corresponds to the methyl group attached to imine carbon (CH_3_C=N). Furthermore, ^13^C-NMR of **5a**–**g** revealed a set of signals that appeared in the aromatic region related to aromatic carbons.

### 2.2. Biology

#### 2.2.1. Cytotoxic Assay against MDA-MB-231 Breast Cancer Cell Line

The newly prepared Schiff bases **2**–**5g** were screened for their in vitro cytotoxic activity against the MDA-MB-231 breast cancer cell line taking CA-4 as a reference drug. From the obtained results presented in Table 1, it is obvious that *N*-ethylidene hydrazinyl derivative 2 revealed weak cytotoxic activity (IC_50_ = 31.59 ± 1.07 μM). Replacement of methyl group in compound **2** by furan ring produced a compound with improved cytotoxic activity (IC_50_ = 6.08 ± 0.42 μM). In addition, further structure extension to give *N*-aryl hydrazinyl molecules **4a**–**5g** produced compounds with considerable cytotoxic activity, which may give an indication that the aryl ring is important for cytotoxic activity. Compounds **4h**, **4j** and **5d** (IC_50_= 2.84 – 1.27 μM) are the most potent in this study and possess cytotoxic activity comparable to reference compound CA-4 (IC_50_ = 0.54 ± 0.04 μM). Furthermore, the 3,5-dibromo-4-hydroxybenzylidene hydrazinyl derivative **4h** has proven to be the most potent cytotoxic compound with IC_50_ = 1.27 ± 0.18 μM. Additionally, the most potent cytotoxic compound showed a higher IC_50_ value (IC_50_= 30.83 ± 2.32 μM) against normal breast cell line MCF-10A; therefore, Schiff base **4h** can be considered to be selective molecules with a safe mode of action toward normal breast cell lines.

#### 2.2.2. Tubulin Polymerization Inhibition Assays

**Figure 3:**
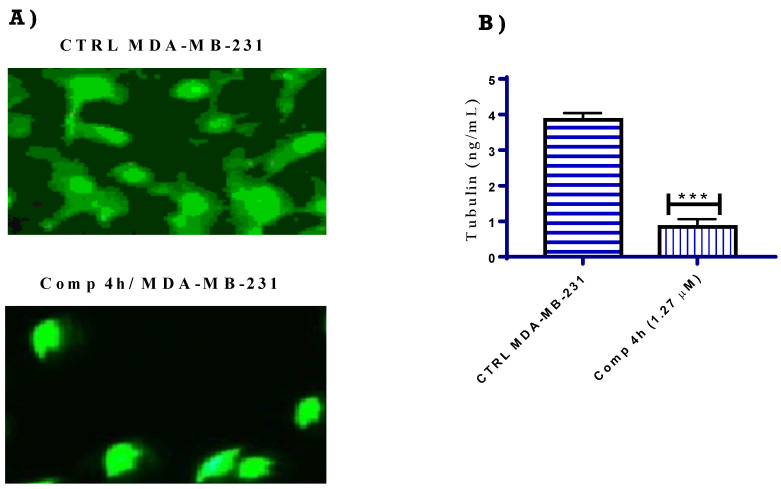
(**A**) Graphical representation of immunofluorescence analysis of tubulin localization in MDA-MB-231 cells after treatment with compound **4h** at its IC_50_ concentration compared with no treatment control. (**B**) Graphical representation of tubulin concentration (ng/mL) before and after treatment with compound **4h**. All values are presented as mean value ± SD and statistical analyses were carried out using one-way analysis of variance (ANOVA) followed by Tukey’s multiple comparison test (*** *p* < 0.001).

Several investigations have shown that TMP-containing compounds such as Col and CA-4 interact with tubulin at the colchicine binding site, inhibiting microtubule assembly [33]. The effects of representative Schiff bases on the inhibition of tubulin assembly were evaluated. Compound **4h** which demonstrated potent cytotoxic activity was assessed at its IC_50_ concentration. Tubulin polymerization inhibitory activity against the MDA-MB-231 cell line was investigated by immunofluorescence analysis using a confocal microscope. The results demonstrated that compound **4h** exhibited good tubulin polymerization inhibition activity compared with the untreated cells (Figure 3A).

In addition, compound **4h** was subjected to in vitro tubulin polymerization inhibitory activity assay using ELISA assay for β-tubulin. As anticipated, compound **4h** inhibited the polymerization of tubulin with a percent inhibition of 78.14% compared with no treatment control (Figure 3B). The results in this study suggested that the molecular target of the prepared Schiff base **4h** is indeed tubulin.

#### 2.2.3. Cell Cycle Effects

**Figure 4:**
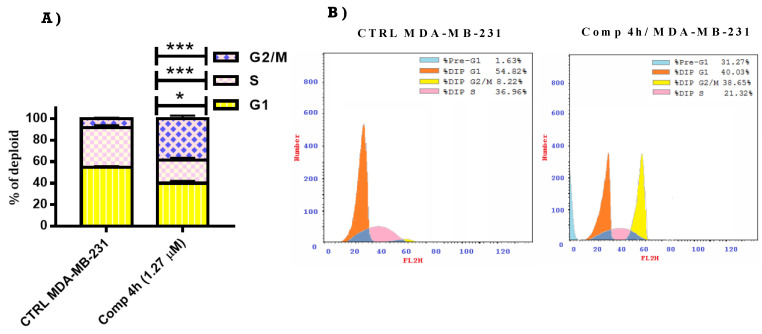
**(A)** Graphical representation for the effect of Schiff base **4h** on the cell cycle profile of MDA-MB-231 cells after 24 h. (**B)** Effect of Schiff base **4h** on the cell cycle profile of MDA-MB-231 cells after 24 h. All values are presented as mean value ± SD and statistical analyses were carried out using one-way analysis of variance (ANOVA) followed by Tukey’s multiple comparison test (* *p* < 0.05, *** *p* < 0.001).

For the purpose of studying the influence of these hydrazides on the cell cycle progression of MDA-MB-231 human malignant cell lines. A FACS analysis was used to determine the DNA proportion of cell nuclei. The treatment of MDA-MB-231 cells with compound **4h** at a concentration equal to its IC_50_ for 24 h induced apoptosis up to 31.27% of the pre-G1 phase and it was accompanied by a concomitant increase in the G2/M phase with a percentage value of 38.65% compared with value 8.22% of untreated control. Therefore, both increases in cells at pre-G1 and G2/M phases clearly show that Schiff base **4h** caused G2/M phase arrest and was effective in causing apoptosis in the case of MDA-MB-231 cancerous cells (Figure 4).

#### 2.2.4. Apoptosis Inducing Effect of Compound 4h

**Figure 5:**
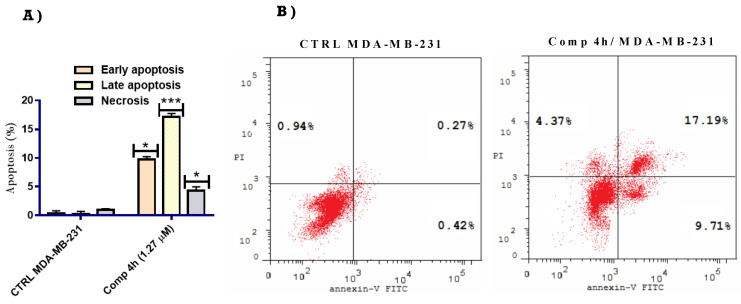
(**A**) Graphical representation for the effect of Schiff base **4h** on the induction of apoptosis in MDA-MB-231 cells after 24 h. (**B**) Effect of Schiff base **4h** on the induction of apoptosis in MDA-MB-231 cells after 24 h. All values are presented as mean value ± SD and statistical analyses were carried out using one-way analysis of variance (ANOVA) followed by Tukey’s multiple comparison test (* *p* < 0.05, *** *p* < 0.001).

A double staining examination of annexin V FITC/propidium iodide (PI) was used to evaluate compound **4h**’s influence on apoptosis and estimate the percentage of the total, early, and late apoptosis in MDA-MB-231 cells. Compound **4h** treatment resulted in a higher proportion of overall apoptosis in the MDA-MB-231 cell line (31.27%) as compared to no treatment controls (1.63%). Furthermore, the proportion of early and late apoptotic cells rose by 23.12- and 63.67-fold, respectively, as compared to the untreated control cells. As supported by cell cycle effects and apoptosis assays of Schiff base **4h** revealed cell growth arrest at the G2/M phase as well as apoptosis-inducing activity (Figure 5).

#### 2.2.5. Mitochondrial Membrane Potential

**Figure 6:**
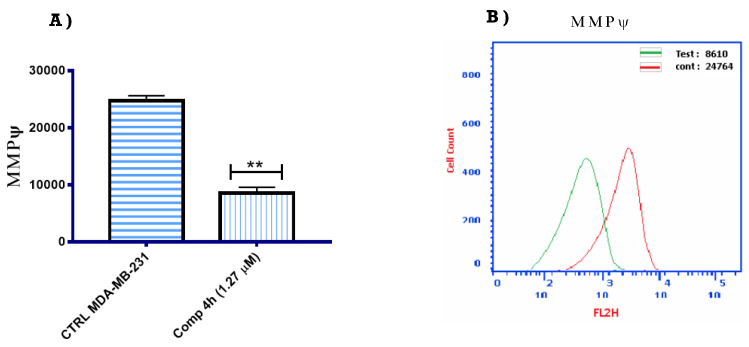
(**A**) Graphical representation for the effect of Schiff base **4h** on the mitochondrial membrane potential in MDA-MB-231 cells after 24 h. (**B**) Effect of Schiff base **4h** on the mitochondrial membrane potential in MDA-MB-231 cells after 24 h. All values are presented as mean value ± SD and statistical analyses were carried out using one-way analysis of variance (ANOVA) followed by Tukey’s multiple comparison test (** *p* < 0.01).

Most of the known anticancer molecules induce apoptotic cascade via activation of the intrinsic pathway via release of cytochrome-c after depolarization of mitochondrial membrane [34]. The potential status of the mitochondrial membrane is determined by the electrochemical gradient (Δψ). In order to investigate the intrinsic pathway of apoptosis, Δψ dissipation was measured by cytofluorometry by exposing the MDA-MB-231 cell line to compound for **4h** at a concentration equal to its IC_50_ for 24 h. The results in Figure 6 revealed a reduction in the level of MDA-MB-231 MMPψ by 65.23% in comparison with untreated cells as measured by flow cytometry. This indicates that compound **4h** induced mitochondrial dissipation in MDA-MB-231 cells, which in turn activated the intrinsic pathway of apoptosis and eventually triggered cell death via the apoptotic pathway.

#### 2.2.6. Effect of Compound 4h on the Expression of p53

**Figure 7:**
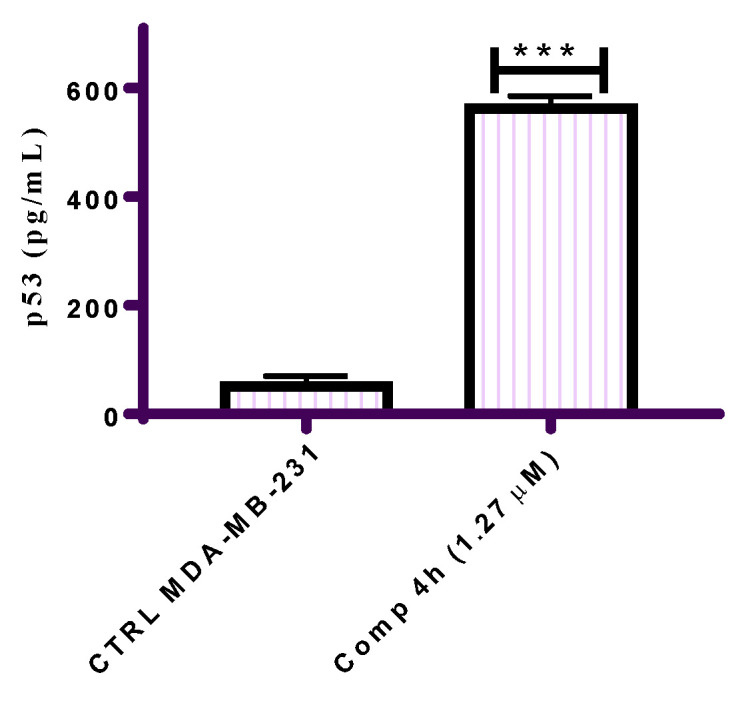
Graphical representation for the effect of Schiff base **4h** on the level of p53 in MDA-MB-231 cells for 24 h. All values are presented as mean value ± SD and statistical analyses were carried out using one-way analysis of variance (ANOVA) followed by Tukey’s multiple comparison test (*** *p* < 0.001).

p53, a tumor suppressor gene, stops the formation of tumors. The activation of such a gene is known to be important in the regulation of the apoptotic pathway induced by various stimuli [35]. In order to understand the effect of TMP-based Schiff base **4h** on p53 dependent apoptotic pathway, the MDA-MB-231 cells were treated with compound **4h** at its IC_50_ concentration for 24 h and the level of p53 was determined using ELISA assay. Figure 7 shows a rise in p53 levels of −10.97 fold when compared to the untreated control.

#### 2.2.7. Effect of Compound 4h on the Expression of Bax and Bcl-2

**Figure 8:**
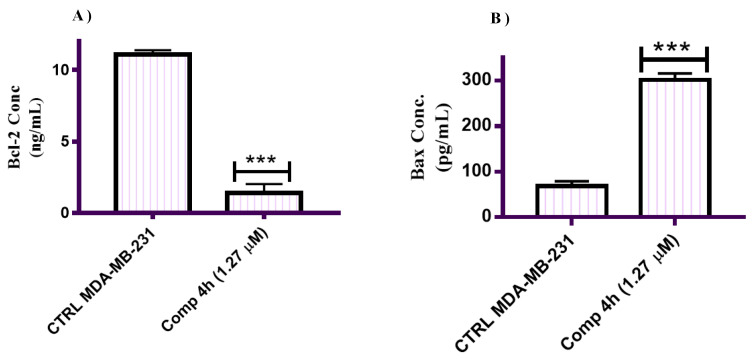
Graphical representation for the effect of Schiff base **4h** on the level of Bcl-2 and Bax in MDA-MB-231 cells for 24 h. (**A**) Effect on Bcl-2 level. (**B**) Effect on Bax level. All values are presented as mean value ± SD and statistical analyses were carried out using one-way analysis of variance (ANOVA) followed by Tukey’s multiple comparison test (*** *p* < 0.001).

To further corroborate apoptosis induction by Schiff base **4h**, the level of anti-apoptotic protein (Bcl-2) and pro-apoptotic protein (Bax) was determined in vitro by ELISA in MDA-MB-231 cells treated with Schiff base **4h**. MDA-MB-231 cells were treated with compound 4h at its IC50 concentration for twenty-four hours. Compound 4h decreased the amount of Bcl-2 in MDA-MB-231 cells by −7.97-fold compared to untreated cells (Figure 8). Furthermore, compound **4h** increased the level of Bax by −4.42-fold compared to untreated cells. As shown by apoptosis experiments, compound 4h induces the intrinsic route of apoptosis by downregulating the levels of Bcl-2 and upregulating the levels of Bax.

#### 2.2.8. Molecular Docking Study

**Figure 9:**
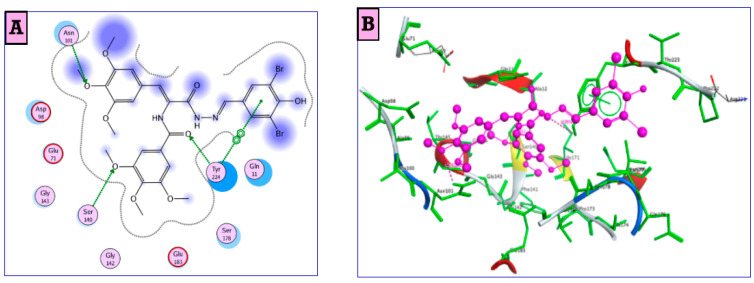
(**A**) show the 2D interactions of Schiff base **4h** into tubulin active site (PDB code: 1SA0). (**B**) show the 3D interactions of Schiff base **4h** into tubulin active site (PDB code: 1SA0).

A molecular docking study with tubulin was performed for *N*-3,5-dibromo-4-hydroxybenzylidene hydrazinyl molecule **4h** into the active site of tubulin crystal structure (PDB: 1SA0) in order to reveal insight into its binding mode. A molecular docking simulation of compound **4h** into the tubulin protein binding site was performed using molecular operating environment (MOE) software, 2015.10. The docking results revealed that *N*-3,5-dibromo-4-hydroxybenzylidene hydrazinyl molecule **4h** displayed good binding affinities (−25.49 kcal/mol). In addition, *N*-3,5-dibromo-4-hydroxybenzylidene hydrazinyl molecule **4h** interacts with the active site of 1SA0 by three hydrogen bonds; Asn 101 with the oxygen of trimethoxyphenyl ring, Ser 140 with the oxygen atom of trimethoxy benzamide ring and Tyr 224 with the carbonyl oxygen of the amide group. Additionally, 3,5-dibromo-4-hydroxyphenyl ring formed π- π interaction with Tyr 224 residue. These results reflect the higher activity and justify the experimental findings of compound **4h** (Figure 9).

### 2.3. Nanostructured Lipid Carrier Fabrication Studies

**Figure 10:**
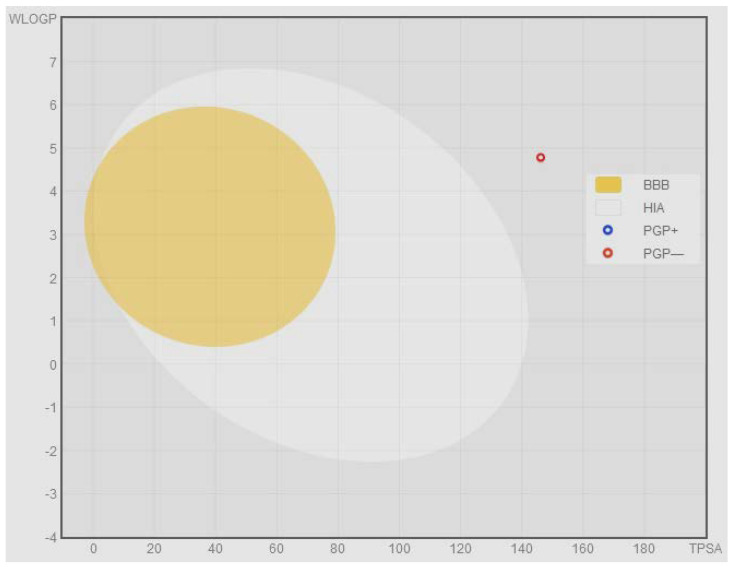
Human intestinal absorption (HIA) and blood-brain barrier (BBB) plot for **4h**.

Aiming to enhance the pharmacokinetic characteristics as very poor aqueous solubility estimated utilizing free web-based tool Swiss ADME (http://www.swissadme.ch/ accessed on 10 April 2022). Remarkably, the anticipated poor water-solubility of **4h** was (LogS_SILICOS-IT = 6.7 × 10^−7^ mg/mL; 9.27 × 10^−10^ mol/L) which will impede its cytotoxic activity and possible clinical outcomes of compound **4h** (Figure 10). Thus, Nanostructured lipid carriers (NLCs) fabrication was proposed as a simple and economic tactic for boosting the kinetic properties of compound **4h**. NLCs aspired to promote the absorption of the compound besides improving its therapeutic response and reduce its predicted side effects. The composition of the eNLC matrix encompasses a blend of lipid molecules of various configurations, originally a gathering of both solid and liquid lipid, which is predisposed to the creation of more crystalline imperfections which in turn grant a greater gap for drug molecules accommodation [36]. Moreover, those crystalline imperfections allow the promotion of drug charging capability, and the suppression of the expulsion of the drug on storage, besides the modulation of the drug release pattern which is correlated to the variation in the composition of the lipid matrix [37]. There are colloidal dispersions of solid lipid and hydrophilic surfactant in the SLN form. Owing to the exclusive characteristics of NLCs, such as minute size, huge surface area, and improved drug loading potentiality [37], thus, loading compound **4h** on NLCs was assumed to promote its cytotoxic activity via densifying its biological manifestations at the tumor site.

### 2.4. Compound 4h Loaded NLCs Design, Preparation and Optimization

Emulsification-ultrasonication technique was adopted for the fabrication of eight drug-loaded NKCs formulations. The design of the formulations was constructed via 2^3^-factorial design using Stat-Ease^®^- V.13 software (Design-Expert TM; Minneapolis, MN, USA) (Table 2). The picked independent variables were the type of lipid (A; X1), lipid concentration (B; X2), along with the concentration of surfactant (C; X3). The resulting 8 formulae were formulated, assessed for the pre-determined dependent responses: encapsulation efficiency (Y1: EE%), particle size (Y2: PS) and zeta potential (Y4: ZP) and analyzed using Stat-Ease^®^- V.13 software. The compositions and the results of the dependent responses of eight drug-NLCs formulations were displayed in Table 2. Drug estimation at different concentrations was performed using HPLC at λmax 254 nm [38].

### 2.5. Fabrication Variables Influence on the Selected Responses

The characterization of the pre-determined responses for each drug-loaded formulae of NLCs: Entrapment Efficiency (Y1: EE%), particle size (Y2: PS) and zeta potential (Y3: ZP). From Table 2, it can be depicted that there was a discrepancy in the outcomes of the measured responses which denoted the great impact of the fabrication variables on the preselected NLCs characters.

**Figure 11:**
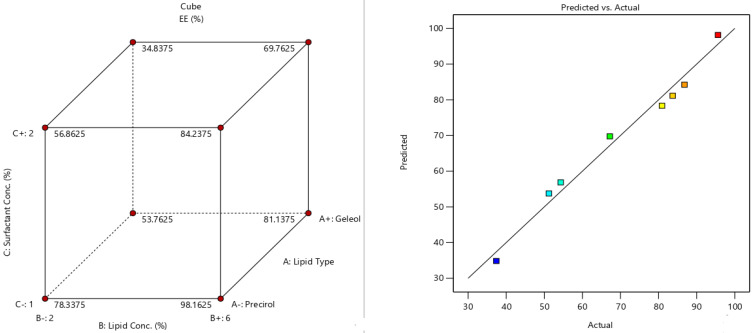
The impact of A: Lipid type, B: Lipid conc., C: surfactant conc. On EE percent of 4h-loaded NLC is represented in cubic plot and predicted versus actual values plot.

The entrapment efficiency of the fabricated NLCs formula ranged from 37.4 ± 1.8 for F6 to 95.6 ± 4.1 for F3 and the impact of the variable’s type of lipid (A; X1), lipid concentration (B; X2), along with the concentration of surfactant (C; X3) on EE% can be obviously displayed by the cubic plot and predicted versus actual values plots (Figure 11). Concerning the type of lipid (A), Precirol ATO-5 originated NLCs formulae exhibited significantly (*p* = 0.0065) greater EE% values than those prepared utilizing Geleol. This may be attributed to the nature of the incorporated lipids which greatly affect the extent of creation of a well-organized crystalline lattice, where lipids enclosing fatty acid acyl glycerol of various chain length grants a higher space for the drug accommodation based on that they were able to configure a less organized particle [39]. Thus, Precirol ATO-5 constituted to form a mixture of various fatty acids of different chains (palmitic C16 and stearic C18) predisposes to a higher possibility to configure less ordered particles than that of Geleol (mono acid acyl glycerol), hence a higher gap for drug loading. Moreover, lipids that possess a blend of diverse chain fatty acids have a greater capability of drug solubilization, and hence, higher drug loading efficiency [40]. Moreover, increasing the lipid concentration (B; X2) from 2% to 6% results in a significant (*p* = 0.0019) increase in EE%. This is plausible as a higher concentration of lipid means a greater amount of lipid was feasible to endorse drug molecules [41]. Meanwhile, increasing surfactant concentration (C; X3) resulted in subsequent significant (*p* = 0.0119) reduction in EE%. The existence of surfactant results in a diminished interfacial tension between the aqueous phase and lipid phase predisposing to the production of particles of diminished size which in turn reduces the NLCs capacity to endorse higher amount of the drug [36,39].

**Figure 12:**
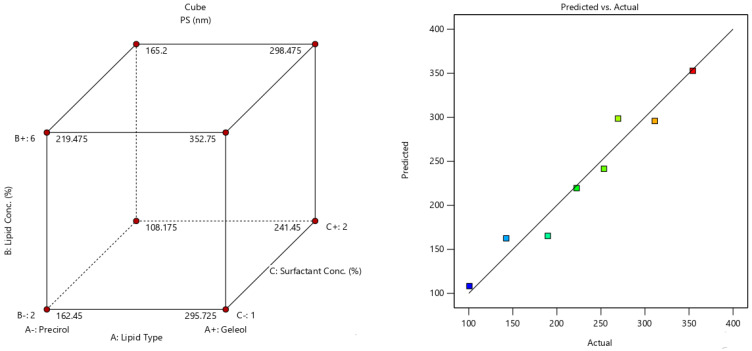
The impact of A: Lipid type, B: Lipid conc., C: surfactant conc. on PS of 4h-loaded NLC is represented in cubic plot and predicted versus actual values plot.

Concerning, PS (Y2) and PDI (Y3) of the formulated NLCs ranged from 100.7 ± 8.1 nm to 354.3 ± 17.3 and 0.28 ± 0.02 to 0.64 ± 0.17, respectively. Figure 12 exposed the 3D surface plots variables type of lipid (A; X1), lipid concentration (B; X2), along with the concentration of surfactant (C; X3) and predicted versus actual values plots of PS. The PDI values give a clue on the degree of homogeneity or heterogeneity of the size of the investigated samples, where the PDI values on getting close to 1 affirm the heterogeneity of the sample and vice versa.

ANOVA results revealed that changing the type of lipid (A; X1) from Precirol ATO-5 to Geleol predisposed to a significant (*p* = 0.0014) elevation in PS, owing to the fact that solid lipid possessing a higher melting point predisposed to gradual crystallization, and thus a higher opportunity to conform more organized crystals with larger particle size [42]. According to Das et al. who found that investigated particle size of formulated clotrimazole NLCs was highly affected by the alteration of type of lipids and following this descending order Compritol < Precirol < Geleol [36]. Moreover, increasing the lipid concentration (B; X2) from 2% to 6% results in significant (*p* = 0.028) enlargement in PS, this may be attributed to the availability of a higher amount of lipids to enclose more drugs, thus increasing the PS. Moreover, increasing the amount of lipids will elevate the system viscosity which in turn will increase the tendency of particle aggregation [43]. On another hand, increasing the surfactant concentration significantly (*p* = 0.0326) reduces the NLCs formulation PS. This can be justified owing to the fact that the increase in the concentration of surfactant will diminish lipid droplet surface tension, which will be able to be subdivided into a finer size. In addition, sufficient surfactant concentration will be able to wrap the minute lipid droplets and hinder their tendency to coalescence, thereby elevating the system stability [44].

**Figure 13:**
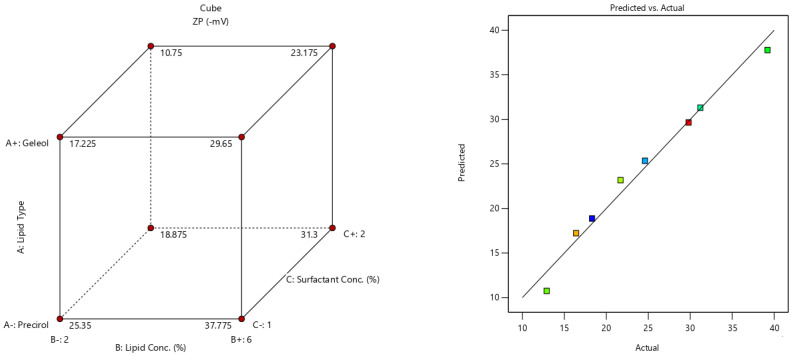
The impact of A: Lipid type, B: Lipid conc., C: surfactant conc. on ZP of 4h-loaded NLC is represented in cubic plot and predicted versus actual values plot.

Turning to ZP (Y4), its values ranged from −12.5 ± 1.8 to −39.2 ± 3.4 Mv denoting the higher stability of produced NLCs as the increase in ZP will subsequently positively affect the system stability. Figure 13 revealed the 3D surface plots variables type of lipid (A; X1), lipid concentration (B; X2), along with the concentration of surfactant (C; X3) and predicted versus actual values plots of ZP. From ANOVA results, it can be depicted that changing the type of lipid (A; X1) from Precirol ATO-5 to Geleol predisposed to a significant (*p* = 0.0021) suppression in ZP and this came in accordance with Das et al., who revealed that the ZP values of the prepared SLN will increase in the following order: Geleol < Precirol < Compritol (1). This may be due to the higher negative charged groups associated with the blend of fatty acid incorporated in Precirol relative to the mono acid acyl in case of Geleol. However, increasing the lipid concentration led to a significant (*p* = 0.0004) elevation in ZP values owing to the higher entrapment of drug-bearing anionic group within the particles, in addition to the increase in anionic groups of the fatty acids on increasing the lipid concentration [39]. Meanwhile, increasing the surfactant concentration significantly (*p* = 0.0048) reduces the ZP values. the virtue of utilizing nonionic surfactant (Cremophore RH 40), which will not impart any additional charge besides the surfactant bulkiness, will aid in disguising the particles charge on surrounding it [39].

### 2.6. Statistical Optimization of Fabrication Variables for Election the Optimum Formula

**Figure 14:**
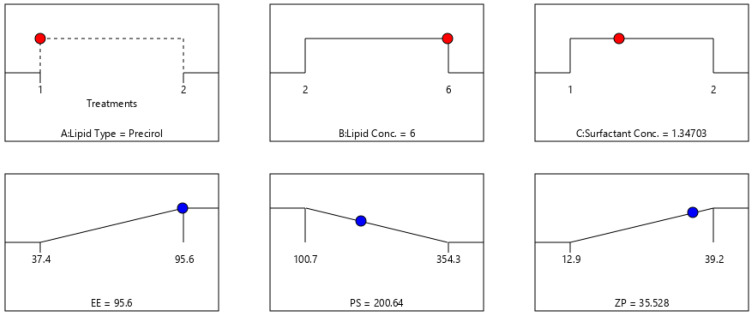
Optimization ramps for the examined independent variables utilize the optimal criteria of the formulation variables for 4h-loaded NLC formulas with the projected value of each measured formulation parameter.

The optimization aimed to pick the best formula that possesses the predetermined criteria as maximum EE%, ZP and diminished PS. This was attained via analysis of the influence of the independent variables on the determined dependent criteria using The Design-Expert V.13 software. Based on 2^3^ factorial design and its subsequent analysis F3 constituted of Precirol ATO 5 as lipid used in concentration 6% *w/v* along with 1% *w/v* Cremophore RH40 was chosen to be the optimum formula possessing a desirability value of 0.805. Moreover, as shown in Table 3 the predicted and actual values of EE%, PS and ZP were not gapped by more than 10% assuring the appropriateness of the design and the liability of its outcomes. (Figure 14) arise the optimum criteria for **4h**-loaded NLCs.

### 2.7. In Vitro Assessment of the Optimum 4h-Loaded NLC

#### 2.7.1. Particle Morphology Analysis via TEM

**Figure 15:**
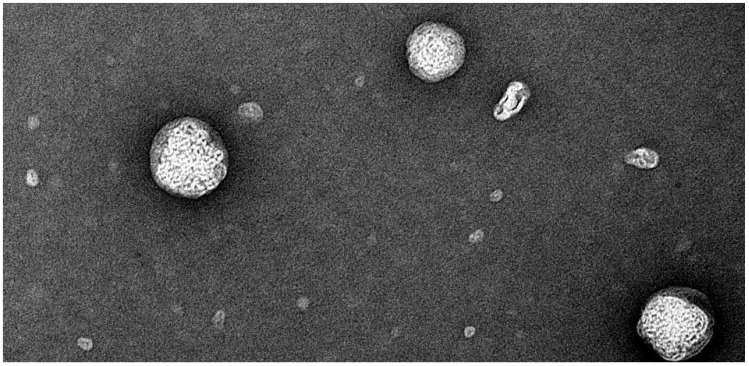
TEM of the picked optimized NLC formula.

(Figure 15) displayed the attained image of the optimum **4h**-loaded NLC formula (F3) using the TEM technique. It can be depicted as the spherical soft-shaped lipid nanoparticles devoid from any crystals for the drug.

#### 2.7.2. In Vitro Drug Release Experiment

**Figure 16:**
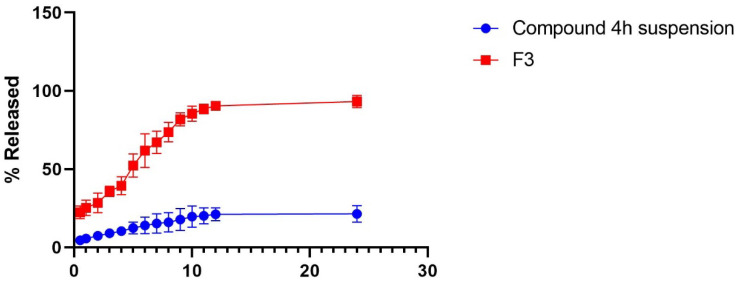
% of compound 4h released ± S.D. from the optimized NLC (F3) compared to that of 4h suspensions.

The release attitude of the drug from drug-loaded NLC relative to drug suspension was conducted by adopting in vitro drug release experiment. The cumulative amount of drug released over 24 h was computed and found to be 92.45% ± 3.37 compared to 20.8% ± 1.2 for **4h** suspension (Figure 16). Thus, NLCs as a carrier for the drug will aid in the extended release of the drug and its densifying in the tumor location in a more solubilized form [38].

#### 2.7.3. Comparative Cytotoxicity Study of Optimized Formula versus Pure 4h

**Figure 17:**
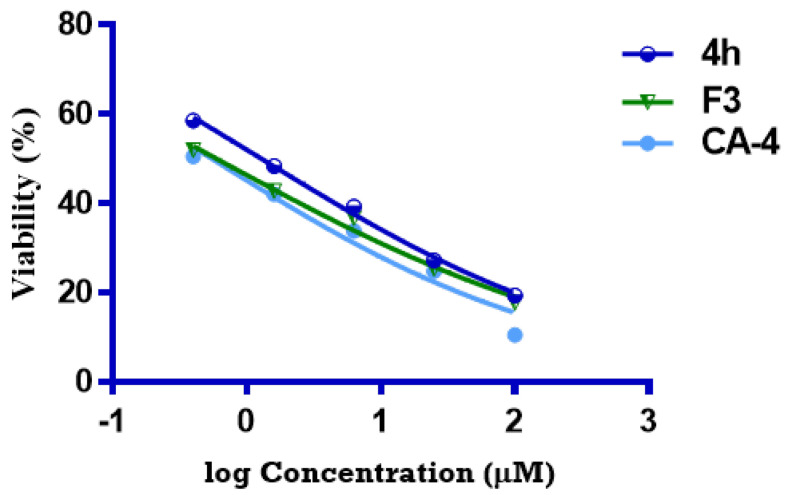
The graphical pattern showing the results of % cell viability of F3 versus 4h and CA-4 as reference drug at multiple concentrations (µM) on MDA-MB-231 cancer cells.

In order to highlight the significance of the drug lodging on NLCs, the cytotoxicity study was conducted for optimized NLCs (F3) relative to the pure drug. (Figure 17) revealed the % viability of MDA-MB-231 cancer cells post-exposure to both preparations and reference drugs. The IC_50_ values of the results were displayed in F3, pure 4h and CA-4 were (0.59 ± 0.07, 1.27 ± 0.18 and 0.54 ± 0.04 μM), respectively. The magnitude of cytotoxicity was found to increase on the following trend: compound **4h** < F3 < CA-4. F3 exhibited the chief cytotoxicity over **4h** and this may be attributed to the large surface area of NLCs, promoted drug solubility and release along with the promotion of the drug cellular uptake, all of these lead to a boost in the anticancer activity.

## 3. Materials and Methods

### 3.1. General

See Supplementary Materials—Appendix SA.

### 3.2. Chemistry

#### 3.2.1. General Procedure for the Preparation of N-((Z)-3-((E)-2-(Ethylidene, furo or aryl)hydrazinyl)-3-oxo-1-(3,4,5-trimethoxyphenyl)prop-1-en-2-yl)-3,4,5-trimethoxybenzamides (2–4l)

Hydrazide **1** (0.46 g, 1 mmol) was added to a solution of an appropriate aldehyde; namely acetaldehyde, furfural or respective aryl aldehyde (1 mmol) in absolute ethanol (20 mL) containing a catalytic amount of acetic acid glacial (1 mL). The reaction mixture was refluxed for 6–8 h and then filtered. The residue obtained was dried, and crystallized from absolute ethanol to yield pure compounds **2**, **3** and **4a**–**l**.

##### N-((Z)-3-((E)-2-Ethylidenehydrazinyl)-3-oxo-1-(3,4,5-trimethoxyphenyl)prop-1-en-2-yl)-3,4,5-trimethoxybenzamide (**2**)

White powder (0.31 g, 63.92%), m.p. 224–226 °C. ^1^H-NMR (400 MHz, DMSO-*d*_6_, δ ppm): 1.89 (s, 3H, CH_3_), 3.64 (s, 6H, 2OCH_3_), 3.66 (s, 3H, OCH_3_), 3.73 (s, 3H, OCH_3_), 3.83 (s, 6H, 2OCH_3_), 6.98 (s, 2H, arom.CH), 7.28 (s, 1H, olefinic CH), 7.40 (s, 2H, arom.CH), 9.85 (s, 1H, CH=N), 9.89 (s, 1H, NH), 10.00 (s, 1H, NH). ^13^C-NMR (100 MHz, DMSO-*d*_6_, δ ppm): 21.08 (CH_3_), 56.07 (2OCH_3_), 56.54 (2OCH_3_), 60.52 (OCH_3_), 60.64 (OCH_3_), 105.99 (C2,6 trimethoxyphenyl), 107.64 (C2,6 trimethoxyphenyl), 128.43 (C olefinic), 129.17 (C1 trimethoxyphenyl), 129.71 (C olefinic), 130.86 (C1 trimethoxybenzamide), 138.58 (C4 trimethoxyphenyl), 140.87 (C4 trimethoxybenzamide), 152.95 (C3,5 trimethoxyphenyl), 153.04 (C3,5 trimethoxybenzamide), 164.60 (C=N), 165.73 (C=O trimethoxybenzamide), 168.55 (C=O hydrazide). Anal. Calcd. for C_24_H_29_N_3_O_8_ (487.50): C, 59.13; H, 6.00; N, 8.62. Found: C, 59.24; H, 6.06; N, 8.67.

##### N-((Z)-3-((E)-2-(Furan-2-ylmethylene)hydrazinyl)-3-oxo-1-(3,4,5-trimethoxyphenyl)prop-1-en-2-yl)-3,4,5-trimethoxybenzamide (**3**)

White powder (0.39 g, 71.82%), m.p. 212–214 °C. ^1^H-NMR (400 MHz, DMSO-*d*_6_, δ ppm): 3.66 (s, 6H, 2OCH_3_), 3.68 (s, 3H, OCH_3_), 3.74 (s, 3H, OCH_3_), 3.84 (s, 6H, 2OCH_3_), 6.64 (s, 1H, furan CH), 6.90 (d, *J* = 3.0 Hz, 1H, furan CH), 7.03 (s, 2H, arom.CH), 7.21 (s, 1H, olefinic CH), 7.44 (s, 2H, arom.CH), 7.85 (s, 1H, furan CH), 8.35 (s, 1H, CH=N), 10.03 (s, 1H, NH), 11.58 (s, 1H, NH). ^13^C-NMR (100 MHz, DMSO-*d*_6_, δ ppm): 56.10 (2OCH_3_), 56.56 (2OCH_3_), 60.56 (OCH_3_), 60.66 (OCH_3_), 105.97 (C2,6 trimethoxyphenyl), 107.79 (C2,6 trimethoxybenzamide), 112.65 (C4 furan), 113.74 (C3 furan), 128.81 (C olefinic), 129.14 (C1 trimethoxyphenyl), 129.79 (C olefinic), 129.93 (C1 trimethoxybenzamide), 137.75 (C=N), 138.64 (C4 trimethoxyphenyl), 141.06 (C4 trimethoxybenzamide), 145.60 (C5 furan), 150.03 (C2 furan), 153.07 (C3,5 trimethoxyphenyl and C3,5 trimethoxybenzamide), 162.48 (C=O trimethoxybenzamide), 165.68 (C=O hydrazide). Anal. Calcd. for C_27_H_29_N_3_O_9_ (539.53): C, 60.11; H, 5.42; N, 7.79. Found: C, 60.03; H, 5.45; N, 7.86.

##### N-((Z)-3-((E)-2-(4-Fluorobenzylidene)hydrazinyl)-3-oxo-1-(3,4,5-trimethoxyphenyl)prop-1-en-2-yl)-3,4,5-trimethoxybenzamide (**4a**)

White powder (0.43 g, 75.03%), m.p. 201–203 °C. ^1^H-NMR (400 MHz, DMSO-*d*_6_, δ ppm): 3.67 (s, 6H, 2OCH_3_), 3.68 (s, 3H, OCH_3_), 3.74 (s, 3H, OCH_3_), 3.84 (s, 6H, 2OCH_3_), 7.03 (s, 2H, arom.CH), 7.21 (s, 1H, olefinic CH), 7.30 (t, *J* = 8.6 Hz, 2H, arom.CH), 7.45 (s, 2H, arom.CH), 7.73–7.83 (m, 2H, arom.CH), 8.44 (s, 1H, CH=N), 10.05 (s, 1H, NH), 11.66 (s, 1H, NH). ^13^C-NMR (400 MHz, DMSO-*d*_6_, δ ppm): 56.11 (2OCH_3_), 56.56 (2OCH_3_), 60.56 (OCH_3_), 60.65 (OCH_3_), 105.97 (C2,6 trimethoxyphenyl), 107.80 (C2,6 trimethoxybenzamide), 116.27 (C3 fluorophenyl), 116.49 (C5 fluorophenyl), 128.79 (C olefinic), 129.19 (C1 trimethoxyphenyl), 129.57 (C olefinic), 129.66 (C1 trimethoxybenzamide), 129.78 (C1 fluorophenyl), 129.87 (C2 fluorophenyl), 131.49 (C6 fluorophenyl), 138.65 (C4 trimethoxyphenyl), 141.06 (C4 trimethoxybenzamide), 146.80 (C=N), 153.08 (C3,5 trimethoxyphenyl and C3,5 trimethoxybenzamide), 162.30 (C4 fluorophenyl), 162.59 (C=O trimethoxybenzamide), 165.72 (C=O hydrazide). Anal. Calcd. for C_29_H_30_FN_3_O_8_ (567.56): C, 61.37; H, 5.33; N, 7.40. Found: C, 61.24; H, 5.36; N, 7.35.

##### N-((Z)-3-((E)-2-(4-Chlorobenzylidene)hydrazinyl)-3-oxo-1-(3,4,5-trimethoxyphenyl)prop-1-en-2-yl)-3,4,5-trimethoxybenzamide (**4b**)

White powder (0.40 g, 69.14%), m.p. 233–235 °C. ^1^H-NMR (400 MHz, DMSO-*d*_6_, δ ppm): 3.66 (s, 6H, 2OCH_3_), 3.67 (s, 3H, OCH_3_), 3.73 (s, 3H, OCH_3_), 3.83 (s, 6H, 2OCH_3_), 7.03 (s, 2H, arom.CH), 7.20 (s, 1H, olefinic CH), 7.44 (s, 2H, arom.CH), 7.52 (d, *J* = 8.1 Hz, 2H, arom.CH), 7.73 (d, *J* = 8.2 Hz, 2H, arom.CH), 8.43 (s, 1H, CH=N), 10.05 (s, 1H, NH), 11.70 (s, 1H, NH). ^13^C-NMR (400 MHz, DMSO-*d*_6_, δ ppm): 56.12 (2OCH_3_), 56.57 (2OCH_3_), 60.57 (OCH_3_), 60.65 (OCH_3_), 105.98 (C2,6 trimethoxyphenyl), 107.82 (C2,6 trimethoxybenzamide), 128.78 (C olefinic), 129.07 (C3,5 chlorophenyl), 129.42 (C2,6 chlorophenyl), 129.76 (C1 trimethoxyphenyl), 129.92 (C olefinic), 133.87 (C1 trimethoxybenzamide), 134.86 (C1 chlorophenyl), 138.66 (C4 chlorophenyl), 141.07 (C4 trimethoxyphenyl), 143.81 (C4 trimethoxybenzamide), 146.54 (C=N), 153.08 (C3,5 trimethoxyphenyl and C3,5 trimethoxybenzamide), 162.63 (C=O trimethoxybenzamide), 165.70 (C=O hydrazide). Anal. Calcd. for C_29_H_30_ClN_3_O_8_ (584.02): C, 59.64; H, 5.18; N, 7.20. Found: C, 59.86; H, 5.30; N, 7.44.

##### 3,4,5-Trimethoxy-N-((Z)-3-((E)-2-(2-nitrobenzylidene)hydrazinyl)-3-oxo-1-(3,4,5-trimethoxyphenyl)prop-1-en-2-yl)benzamide (**4c**)

White powder (0.35 g, 58.92%), m.p. 239–241 °C. ^1^H-NMR (400 MHz, DMSO-*d*_6_, δ ppm): 3.69 (s, 9H, 3OCH_3_), 3.75 (s, 3H, OCH_3_), 3.85 (s, 6H, 2OCH_3_), 7.06 (s, 2H, arom.CH), 7.23 (s, 1H, olefinic CH), 7.46 (s, 2H, arom.CH), 7.69 (t, *J* = 7.5 Hz, 1H, arom.CH), 7.82 (d, *J* = 7.0 Hz, 1H, arom.CH), 8.08 (d, *J* = 7.9 Hz, 1H, arom.CH), 8.13 (d, *J* = 7.5 Hz, 1H, arom.CH), 8.85 (s, 1H, CH=N), 10.10 (s, 1H, NH), 12.02 (s, 1H, NH). ^13^C-NMR (400 MHz, DMSO-*d*_6_, δ ppm): 56.14 (2OCH_3_), 56.57 (2OCH_3_), 60.57 (OCH_3_), 60.66 (OCH_3_), 105.97 (C2,6 trimethoxyphenyl), 107.88 (C2,6 trimethoxybenzamide), 125.08 (C3 nitrophenyl), 128.35 (C olefinic), 128.69 (C1 trimethoxyphenyl), 129.00 (C olefinic), 129.31 (C1 nitrophenyl), 129.70 (C1 trimethoxybenzamide), 130.19 (C6 nitrophenyl), 131.02 (C4 nitrophenyl), 134.16 (C5 nitrophenyl), 141.11 (C4 trimethoxyphenyl), 142.78 (C4 trimethoxybenzamide), 148.71 (C=N), 153.10 (C3,5 trimethoxyphenyl), 153.11 (C3,5 trimethoxybenzamide), 162.89 (C=O trimethoxybenzamide), 164.69 (C2 nitrophenyl), 165.76 (C=O hydrazide). Anal. Calcd. for C_29_H_30_N_4_O_10_ (594.57): C, 58.58; H, 5.09; N, 9.42. Found: C, 58.39; H, 5.12; N, 9.37.

##### N-((Z)-3-((E)-2-(2-Hydroxybenzylidene)hydrazinyl)-3-oxo-1-(3,4,5-trimethoxyphenyl)prop-1-en-2-yl)-3,4,5-trimethoxybenzamide (**4d**)

White powder (0.35 g, 61.17%), m.p. 220–222 °C. ^1^H-NMR (400 MHz, DMSO-*d*_6_, δ ppm): 3.67 (s, 6H, 2OCH_3_), 3.68 (s, 3H, OCH_3_), 3.75 (s, 3H, OCH_3_), 3.85 (s, 6H, 2OCH_3_), 6.93 (t, *J* = 8.2 Hz, 2H, arom.CH), 7.04 (s, 2H, arom.CH), 7.27 (s, 1H, olefinic CH), 7.28–7.34 (m, 1H, arom.CH), 7.46 (s, 2H, arom.CH), 7.51 (d, *J* = 7.4 Hz, 1H, arom.CH), 8.63 (s, 1H, CH=N), 10.07 (s, 1H, NH), 11.32 (s, 1H, OH), 11.89 (s, 1H, NH). ^13^C-NMR (100 MHz, DMSO-*d*_6_, δ ppm): 56.12 (2OCH_3_), 56.58 (2OCH_3_), 60.57 (OCH_3_), 60.67 (OCH_3_), 106.00 (C2,6 trimethoxyphenyl), 107.85 (C2,6 trimethoxybenzamide), 116.87 (C3 hydroxyphenyl), 119.19 (C1 hydroxyphenyl), 119.81 (C5 hydroxyphenyl), 128.70 (C olefinic), 128.75 (C1 trimethoxyphenyl), 129.68 (C6 hydroxyphenyl), 129.95 (C olefinic), 130.39 (C1 trimethoxybenzamide), 131.76 (C4 hydroxyphenyl), 138.76 (C4 trimethoxyphenyl), 141.10 (C4 trimethoxybenzamide), 148.57 (C=N), 153.09 (C3,5 trimethoxyphenyl and C3,5 trimethoxybenzamide), 157.90 (C2 hydroxyphenyl), 162.31 (C=O trimethoxybenzamide), 165.75 (C=O hydrazide). Anal. Calcd. for C_29_H_31_N_3_O_9_ (565.57): C, 61.59; H, 5.52; N, 7.43. Found: C, 61.87; H, 5.54; N, 7.47.

##### N-((Z)-3-((E)-2-(4-Hydroxybenzylidene)hydrazinyl)-3-oxo-1-(3,4,5-trimethoxyphenyl)prop-1-en-2-yl)-3,4,5-trimethoxybenzamide (**4e**)

White powder (0.31 g, 54.27%), m.p. 255–257 °C. ^1^H-NMR (400 MHz, DMSO-*d*_6_, δ ppm): 3.68 (s, 3H, OCH_3_). 3.68 (s, 6H, 2OCH_3_), 3.74 (s, 3H, OCH_3_), 3.84 (s, 6H, 2OCH_3_), 7.05 (s, 2H, arom.CH), 7.23 (s, 1H, olefinic CH), 7.45 (s, 2H), 7.97 (d, *J* = 7.9 Hz, 2H, arom.CH), 8.31 (d, *J* = 7.9 Hz, 2H, arom.CH), 8.55 (s, 1H, CH=N), 10.10 (s, 1H, NH), 11.95 (s, 1H, NH). ^13^C-NMR (100 MHz, DMSO-*d*_6_, δ ppm): 56.13 (2OCH_3_), 56.57 (2OCH_3_), 60.58 (OCH_3_), 60.66 (OCH_3_), 105.97 (C2,6 trimethoxyphenyl), 107.88 (C2,6 trimethoxybenzamide), 124.57 (C olefinic), 128.35 (C3,5 hydroxyphenyl), 128.69 (C1 trimethoxyphenyl and C1 trimethoxybenzamide), 129.68 (C olefinic), 133.69 (C1 hydroxyphenyl), 138.75 (C2,6 hydroxyphenyl), 138.89 (C4 trimethoxyphenyl), 141.24 (C4 trimethoxybenzamide), 145.33 (C=N), 151.73 (C4 hydroxyphenyl), 153.10 (C3,5 trimethoxyphenyl and C3,5 trimethoxybenzamide), 163.18 (C=O trimethoxybenzamide), 165.74 (C=O hydrazide). Anal. Calcd. for C_29_H_31_N_3_O_9_ (565.57): C, 61.59; H, 5.52; N, 7.43. Found: C, 61.43; H, 5.56; N, 7.58.

##### 3,4,5-Trimethoxy-N-((Z)-3-((E)-2-(4-methylbenzylidene)hydrazinyl)-3-oxo-1-(3,4,5-trimethoxyphenyl)prop-1-en-2-yl)benzamide (**4f**)

White powder (0.40 g, 70.38%), m.p. 208–210 °C. ^1^H-NMR (400 MHz, DMSO-*d*_6_, δ ppm): 2.35 (s, 3H, CH_3_), 3.67 (s, 6H, 2OCH_3_), 3.68 (s, 3H, OCH_3_), 3.74 (s, 3H, OCH_3_), 3.84 (s, 6H, 2OCH_3_), 7.03 (s, 2H, arom.CH), 7.21 (s, 1H, olefinic CH), 7.28 (d, *J* = 7.8 Hz, 2H, arom.CH), 7.45 (s, 2H, arom.CH), 7.61 (d, *J* = 7.8 Hz, 2H, arom.CH), 8.41 (s, 1H, CH=N), 10.04 (s, 1H, NH), 11.57 (s, 1H, NH). ^13^C-NMR (100 MHz, DMSO-*d*_6_, δ ppm): 21.50 (CH_3_), 56.11 (2OCH_3_), 56.56 (2OCH_3_), 60.56 (OCH_3_), 60.65 (OCH_3_), 105.98 (C2,6 trimethoxyphenyl), 107.79 (C2,6 trimethoxybenzamide), 127.44 (C2,6 methylphenyl), 128.83 (C olefinic), 129.28 (C1 trimethoxyphenyl), 129.74 (C olefinic), 129.82 (C1 trimethoxybenzamide), 129.92 (C3,5 methylphenyl), 132.20 (C1 methylphenyl), 138.61 (C4 trimethoxyphenyl), 140.27 (C4 methylphenyl), 141.05 (C4 trimethoxybenzamide), 147.97 (C=N), 153.07 (C3,5 trimethoxyphenyl and C3,5 trimethoxybenzamide), 162.47 (C=O trimethoxybenzamide), 165.69 (C=O hydrazide).Anal. Calcd. for C_30_H_33_N_3_O_8_ (563.60): C, 63.93; H, 5.90; N, 7.46. Found: C, 63.72; H, 5.78; N, 7.57.

##### 3,4,5-Trimethoxy-N-((Z)-3-((E)-2-(4-methoxybenzylidene)hydrazinyl)-3-oxo-1-(3,4,5-trimethoxyphenyl)prop-1-en-2-yl)benzamide (**4g**)

White powder (0.37 g, 64.51%), m.p. 217–219 °C. ^1^H-NMR (400 MHz, DMSO-*d*_6_, δ ppm): 3.66 (s, 6H, 2OCH_3_), 3.68 (s, 3H, OCH_3_), 3.74 (s, 3H, OCH_3_), 3.82 (s, 3H, OCH_3_), 3.84 (s, 6H, 2OCH_3_), 7.03 (d, *J* = 5.5 Hz, 4H, arom.CH), 7.20 (s, 1H, olefinic CH), 7.45 (s, 2H, arom.CH), 7.65 (d, *J* = 8.5 Hz, 2H, arom.CH), 8.38 (s, 1H, CH=N), 10.02 (s, 1H, NH), 11.50 (s, 1H, NH). ^13^C-NMR (100 MHz, DMSO-*d*_6_, δ ppm): 55.76 (OCH_3_), 56.10 (2OCH_3_), 56.56 (2OCH_3_), 60.56 (OCH_3_), 60.65 (OCH_3_), 105.98 (C2,6 trimethoxyphenyl), 107.77 (C2,6 trimethoxybenzamide), 114.82 (C3,5 methoxyphenyl), 127.46 (C1 methoxyphenyl), 128.86 (C olefinic), 129.05 (C2,6 methoxyphenyl), 129.33 (C olefinic), 129.67 (C1 trimethoxyphenyl), 129.86 (C1 trimethoxybenzamide), 138.59 (C4 trimethoxyphenyl), 141.04 (C4 trimethoxybenzamide), 147.85 (C=N), 153.07 (C3,5 trimethoxyphenyl and C3,5 trimethoxybenzamide), 161.24 (C4 methoxyphenyl), 162.35 (C=O trimethoxybenzamide), 165.68 (C=O hydrazide). Anal. Calcd. for C_30_H_33_N_3_O_9_ (579.60): C, 62.17; H, 5.74; N, 7.25. Found: C, 61.99; H, 5.77; N, 7.28.

##### N-((Z)-3-((E)-2-(3,5-Dibromo-4-hydroxybenzylidene)hydrazinyl)-3-oxo-1-(3,4,5-trimethoxyphenyl)prop-1-en-2-yl)-3,4,5-trimethoxybenzamide (**4h**)

White powder (0.48 g, 66.03%), m.p. 198–200 °C. ^1^H-NMR (400 MHz, DMSO-*d*_6_, δ ppm): 3.67 (s, 3H, OCH_3_), 3.68 (s, 6H, 2OCH_3_), 3.74 (s, 3H, OCH_3_), 3.84 (s, 6H, 2OCH_3_), 7.03 (s, 2H, arom.CH), 7.19 (s, 1H, olefinic CH), 7.44 (s, 2H, arom.CH), 7.88 (s, 2H, arom.CH), 8.29 (s, 1H, CH=N), 10.04 (s, 1H, NH), 10.43 (s, 1H, OH), 11.73 (s, 1H, NH). ^13^C-NMR (100 MHz, DMSO-*d*_6_, δ ppm): 56.12 (2OCH_3_), 56.56 (2OCH_3_), 60.57 (2OCH_3_), 60.66 (OCH_3_), 105.96 (C2,6 trimethoxyphenyl), 107.81 (C2,6 trimethoxybenzamide), 112.70 (C3,5 dibromophenyl), 128.79 (C olefinic), 129.16 (C1 trimethoxyphenyl), 129.51 (C olefinic), 129.77 (C1 trimethoxybenzamide), 129.83 (C1 dibromophenyl), 130.97 (C2,6 dibromophenyl), 138.64 (C4 trimethoxyphenyl), 141.06 (C4 trimethoxybenzamide), 144.84 (C=N), 152.56 (C4 dibromophenyl), 153.08 (C3,5 trimethoxyphenyl and C3,5 trimethoxybenzamide), 162.63 (C=O trimethoxybenzamide), 165.69 (C=O hydrazide). Anal. Calcd. for C_29_H_29_Br_2_N_3_O_9_ (723.36): C, 48.15; H, 4.04; N, 5.81. Found: C, 48.31; H, 4.01; N, 5.78.

##### N-((Z)-3-((E)-2-(4-(Dimethylamino)benzylidene)hydrazinyl)-3-oxo-1-(3,4,5-trimethoxyphenyl)prop-1-en-2-yl)-3,4,5-trimethoxybenzamide (**4i**)

White powder (0.37 g, 61.82%), m.p. 243–245 °C. ^1^H-NMR (400 MHz, DMSO-*d*_6_, δ ppm): 2.98 (s, 6H, 2CH_3_), 3.66 (s, 6H, 2OCH_3_), 3.67 (s, 3H, OCH_3_), 3.74 (s, 3H, OCH_3_), 3.84 (s, 6H, 2OCH_3_), 6.76 (d, *J* = 8.7 Hz, 2H, arom.CH), 7.02 (s, 2H, arom.CH), 7.20 (s, 1H, olefinic CH), 7.44 (s, 2H, arom.CH), 7.52 (d, *J* = 8.7 Hz, 2H, arom.CH), 8.29 (s, 1H, CH=N), 10.00 (s, 1H, NH), 11.32 (s, 1H, NH). ^13^C-NMR (100 MHz, DMSO-*d*_6_, δ ppm): 40.25 (2CH_3_), 56.09 (2OCH_3_), 56.56 (2OCH_3_), 60.55 (OCH_3_), 60.65 (OCH_3_), 105.99 (C2,6 trimethoxyphenyl), 107.72 (C2,6 trimethoxybenzamide), 112.31 (C3,5 dimethylaminophenyl), 122.20 (C1 dimethylaminophenyl), 128.79 (C2,6 dimethylaminophenyl), 128.95 (C olefinic), 129.46 (C1 trimethoxyphenyl), 129.48 (C olefinic), 129.94 (C1 trimethoxybenzamide), 138.52 (C4 trimethoxyphenyl), 141.01 (C4 trimethoxybenzamide), 148.83 (C=N), 151.94 (C4 dimethylaminophenyl), 153.05 (C3,5 trimethoxyphenyl and C3,5 trimethoxybenzamide), 162.02 (C=O trimethoxybenzamide), 165.66 (C=O hydrazide). Anal. Calcd. for C_31_H_36_N_4_O_8_ (592.64): C, 62.83; H, 6.12; N, 9.45. Found: C, 62.64; H, 6.04; N, 9.41.

##### N-((Z)-3-((E)-2-(4-Hydroxy-3-methoxybenzylidene)hydrazinyl)-3-oxo-1-(3,4,5-trimethoxyphenyl)prop-1-en-2-yl)-3,4,5-trimethoxybenzamide (**4j**)

White powder (0.32 g, 54.08%), m.p. 226–228 °C. ^1^H-NMR (400 MHz, DMSO-*d*_6_, δ ppm): 3.66 (s, 6H, 2OCH_3_), 3.68 (s, 3H, OCH_3_), 3.74 (s, 3H, OCH_3_), 3.81 (s, 3H, OCH_3_), 3.85 (s, 6H, 2OCH_3_), 6.98 (d, *J* = 8.3 Hz, 1H, arom.CH), 7.03 (d, *J* = 7.0 Hz, 3H, arom.CH), 7.21 (s, 1H, olefinic CH), 7.26 (s, 1H, arom.CH), 7.45 (s, 2H, arom.CH), 8.29 (s, 1H, CH=N), 9.31 (s, 1H, OH), 10.01 (s, 1H, NH), 11.45 (s, 1H, NH). ^13^C-NMR (100 MHz, DMSO-*d*_6_, δ ppm): 56.04 (OCH_3_), 56.10 (2OCH_3_), 56.56 (2OCH_3_), 60.56 (OCH_3_), 60.66 (OCH_3_), 105.98 (C2,6 trimethoxyphenyl), 107.76 (C2,6 trimethoxybenzamide), 112.37 (C2 hydroxymethoxyphenyl), 112.71 (C5 hydroxymethoxyphenyl), 120.61 (C6 hydroxymethoxyphenyl), 127.76 (C olefinic), 128.87 (C1 trimethoxyphenyl), 129.32 (C olefinic), 129.69 (C1 trimethoxybenzamide), 129.87 (C1 hydroxymethoxyphenyl), 138.58 (C4 trimethoxyphenyl), 141.04 (C4 trimethoxybenzamide), 147.35 (C=N), 148.12 (C3 hydroxymethoxyphenyl), 150.19 (C4 hydroxymethoxyphenyl), 153.07 (C3,5 trimethoxyphenyl and C3,5 trimethoxybenzamide), 162.28 (C=O trimethoxybenzamide), 165.68 (C=O hydrazide). Anal. Calcd. for C_30_H_33_N_3_O_10_ (595.60): C, 60.50; H, 5.58; N, 7.06. Found: C, 60.36; H, 5.56; N, 7.09.

##### N-((Z)-3-((E)-2-(3,5-Dimethoxybenzylidene)hydrazinyl)-3-oxo-1-(3,4,5-trimethoxyphenyl)prop-1-en-2-yl)-3,4,5-trimethoxybenzamide (**4k**)

White powder (0.35 g, 57.12%), m.p. 214–216 °C. ^1^H-NMR (400 MHz, DMSO-*d*_6_, δ ppm): 3.68 (s, 3H, OCH_3_), 3.68 (s, 6H, 2OCH_3_), 3.75 (s, 3H, OCH_3_), 3.80 (s, 6H, 2OCH_3_), 3.85 (s, 6H, 2OCH_3_), 6.58 (s, 1H, arom.CH), 6.86 (s, 2H, arom.CH), 7.04 (s, 2H, arom.CH), 7.19 (s, 1H, olefinic CH), 7.45 (s, 2H, arom.CH), 8.37 (s, 1H, CH=N), 10.07 (s, 1H, NH), 11.67 (s, 1H, NH). ^13^C-NMR (100 MHz, DMSO-*d*_6_, δ ppm): 55.80 (2OCH_3_), 56.12 (2OCH_3_), 56.55 (2OCH_3_), 60.57 (OCH_3_), 60.65 (OCH_3_), 102.64 (C4 dimethoxyphenyl), 105.24 (C2,6 dimethoxyphenyl), 105.96 (C2,6 trimethoxyphenyl), 107.81 (C2,6 trimethoxybenzamide), 128.80 (C olefinic), 129.27 (C1 trimethoxyphenyl), 129.73 (C olefinic), 129.78 (C1 trimethoxybenzamide), 136.93 (C1 dimethoxyphenyl), 138.64 (C4 trimethoxyphenyl), 141.06 (C4 trimethoxybenzamide), 147.89 (C=N), 153.08 (C3,5 trimethoxyphenyl and C3,5 trimethoxybenzamide), 161.16 (C3,5 dimethoxyphenyl), 162.62 (C=O trimethoxybenzamide), 165.72 (C=O hydrazide). Anal. Calcd. for C_31_H_35_N_3_O_10_ (609.62): C, 61.08; H, 5.79; N, 6.89. Found: C, 61.24; H, 5.80; N, 6.94.

##### 3,4,5-Trimethoxy-N-((Z)-3-oxo-3-((E)-2-(3,4,5-trimethoxybenzylidene)hydrazinyl)-1-(3,4,5-trimethoxyphenyl)prop-1-en-2-yl)benzamide (**4l**)

White powder (0.32 g, 49.74%), m.p. 192–194 °C. ^1^H-NMR (400 MHz, DMSO-*d*_6_, δ ppm): 3.68 (s, 9H, 3OCH_3_), 3.72 (s, 3H, OCH_3_), 3.74 (s, H, OCH_3_), 3.84 (s, 12H, 4OCH_3_), 7.01 (s, 2H, arom.CH), 7.03 (s, 2H, arom.CH), 7.44 (s, 2H, arom.CH), 8.38 (s, 1H, olefinic CH), 10.06 (s, 1H, NH), 11.63 (s, 1H, NH). ^13^C-NMR (100 MHz, DMSO-*d*_6_, δ ppm): 56.12 (2OCH_3_), 56.41 (2OCH_3_), 56.55 (2OCH_3_), 60.57 (2OCH_3_), 60.65 (OCH_3_), 104.68 (C2,6 trimethoxyphenyl), 105.96 (C2,6 trimethoxybenzylidene), 107.79 (C2,6 trimethoxybenzamide), 128.82 (C olefinic), 129.36 (C1 trimethoxyphenyl), 129.57 (C olefinic), 129.80 (C1 trimethoxybenzylidene), 130.39 (C1 trimethoxybenzamide), 138.62 (C4 trimethoxyphenyl), 139.62 (C4 trimethoxybenzylidene), 141.04 (C4 trimethoxybenzamide), 148.07 (C=N), 153.08 (C3,5 trimethoxyphenyl and C3,5 trimethoxybenzamide), 153.66 (C3,5 trimethoxybenzylidene), 162.53 (C=O trimethoxybenzamide), 165.73 (C=O hydrazide). Anal. Calcd. for C_32_H_37_N_3_O_11_ (639.65): C, 60.09; H, 5.83; N, 6.57. Found: C, 59.97; H, 5.86; N, 6.48.

#### 3.2.2. General Procedure for the Preparation of N-((Z)-3-((E)-2-(Arylethylidene)hydrazinyl)-3-oxo-1-(3,4,5-trimethoxyphenyl)prop-1-en-2-yl)-3,4,5-trimethoxybenzamides **5a**–**g**

A mixture of hydrazide **1** (0.004 mol) and respective aryl halide (0.004 mol) in n-butanol (15 mL) was refluxed for 6–8 h. The obtained solid product was filtered off, dried and crystallized from ethanol/water (3:1) to give pure hydrazinyl compound **5a**–**g**.

##### 3,4,5-Trimethoxy-N-((Z)-3-oxo-3-((E)-2-(1-phenylethylidene)hydrazinyl)-1-(3,4,5-trimethoxyphenyl)prop-1-en-2-yl)benzamide (**5a**)

White powder (0.37 g, 66.19%), m.p. 260–262 °C. ^1^H-NMR (400 MHz, DMSO-*d*_6_, δ ppm): ^1^H-NMR (400 MHz, DMSO-*d*_6_, δ ppm): 2.31 (s, 3H, CH_3_), 3.69 (s, 9H, 3OCH_3_), 3.74 (s, 3H, OCH_3_), 3.84 (s, 6H, 2OCH_3_), 7.05 (s, 2H, arom.CH), 7.22 (s, 1H, olefinic CH), 7.42 (s, 5H, arom.CH), 7.84 (s, 2H, arom.CH), 10.09 (s, 1H, NH), 10.58 (s, 1H, NH). ^13^C-NMR (100 MHz, DMSO-*d*_6_, δ ppm): 14.97 (CH_3_), 56.15 (2OCH_3_), 56.54 (2OCH_3_), 60.57 (OCH_3_), 60.62 (OCH_3_), 105.84 (C2,6 trimethoxyphenyl), 107.84 (C2,6 trimethoxybenzamide), 126.86 (C2,6 phenyl), 128.78 (C3,5 phenyl), 128.86 (C olefinic and C1 trimethoxyphenyl), 129.28 (C olefinic), 129.94 (C1 trimethoxybenzamide), 132.29 (C4 phenyl), 138.51 (C5 phenyl), 138.58 (C4 trimethoxyphenyl), 140.98 (C4 trimethoxybenzamide), 149.84 (C=N), 153.06 (C3,5 trimethoxyphenyl), 153.09 (C3,5 trimethoxybenzamide), 162.50 (C=O trimethoxybenzamide), 165.93 (C=O hydrazide). Anal. Calcd. for C_30_H_33_N_3_O_8_ (563.60): C, 63.93; H, 5.90; N, 7.46. Found: C, 64.18; H, 6.08; N, 7.57.

##### N-((Z)-3-((E)-2-(1-(4-Chlorophenyl)ethylidene)hydrazinyl)-3-oxo-1-(3,4,5-trimethoxyphenyl)prop-1-en-2-yl)-3,4,5-trimethoxybenzamide (**5b**)

White powder (0.36 g, 59.73%), m.p. 249–251 °C. ^1^H-NMR (400 MHz, DMSO-*d*_6_, δ ppm): 2.30 (s, 3H, CH_3_), 3.69 (s, 6H, 2OCH_3_), 3.69 (s, 3H, OCH_3_), 3.74 (s, 3H, OCH_3_), 3.83 (s, 6H, 2OCH_3_), 7.05 (s, 2H, arom.CH), 7.19 (s, 1H, olefinic CH), 7.41 (s, 2H, arom.CH), 7.50 (s, 2H, arom.CH), 7.85 (s, 2H, arom.CH), 10.10 (s, 1H, NH), 10.63 (s, 1H, NH). ^13^C-NMR (100 MHz, DMSO-*d*_6_, δ ppm): 14.87 (CH_3_), 56.16 (2OCH_3_), 56.54 (2OCH_3_), 60.57 (OCH_3_), 60.62 (OCH_3_), 105.83 (C2,6 trimethoxyphenyl), 107.85 (C2,6 trimethoxybenzamide), 128.60 (C2,6 chlorophenyl), 128.83 (C3,5 chlorophenyl), 129.34 (C olefinic), 129.75 (C1 trimethoxyphenyl), 129.91 (C olefinic), 130.72 (C1 trimethoxybenzamide), 134.53 (C1 chlorophenyl), 137.34 (C4 chlorophenyl), 138.60 (C4 trimethoxyphenyl), 140.98 (C4 trimethoxybenzamide), 148.64 (C=N), 153.07 (C3,5 trimethoxyphenyl), 153.09 (C3,5 trimethoxybenzamide), 162.59 (C=O trimethoxybenzamide), 165.95 (C=O hydrazide). Anal. Calcd. for C_30_H_32_ClN_3_O_8_ (598.04): C, 60.25; H, 5.39; N, 7.03. Found: C, 59.98; H, 5.17; N, 7.22.

##### N-((Z)-3-((E)-2-(1-(4-Bromophenyl)ethylidene)hydrazinyl)-3-oxo-1-(3,4,5-trimethoxyphenyl)prop-1-en-2-yl)-3,4,5-trimethoxybenzamide (**5c**)

White powder (0.39 g, 61.10%), m.p. 244–246 °C. ^1^H-NMR (400 MHz, DMSO-*d*_6_, δ ppm): 2.29 (s, 3H, CH_3_), 3.69 (s, 3H, OCH_3_), 3.69 (s, 6H, 2OCH_3_), 3.74 (s, 3H, OCH_3_), 3.83 (s, 6H, 2OCH_3_), 7.05 (s, 2H, arom.CH), 7.19 (s, 1H, olefinic CH), 7.41 (s, 2H, arom.CH), 7.64 (s, 2H, arom.CH), 7.78 (s, 2H, arom.CH), 10.10 (s, 1H, NH), 10.63 (s, 1H, NH). ^13^C-NMR (100 MHz, DMSO-*d*_6_, δ ppm): 14.82 (CH_3_), 56.16 (2OCH_3_), 56.54 (2OCH_3_), 60.58 (OCH_3_), 60.62 (OCH_3_), 105.83 (C2,6 trimethoxyphenyl), 107.86 (C2,6 trimethoxybenzamide), 125.78 (C4 bromophenyl), 128.82 (C2,6 bromophenyl), 128.87 (C3,5 bromophenyl), 129.84 (C olefinic), 129.90 (C1 trimethoxyphenyl), 131.09 (C olefinic), 131.74 (C1 trimethoxybenzamide), 137.70 (C1 bromophenyl), 138.60 (C4 trimethoxyphenyl), 140.99 (C4 trimethoxybenzamide), 145.56 (C=N), 153.07 (C3,5 trimethoxyphenyl), 153.09 (C3,5 trimethoxybenzamide), 161.06 (C=O trimethoxybenzamide), 165.89 (C=O hydrazide). Anal. Calcd. for C_30_H_32_BrN_3_O_8_ (642.49): C, 56.08; H, 5.02; N, 6.54. Found: C, 55.86; H, 4.92; N, 6.79.

##### N-((Z)-3-((E)-2-(1-(4-Hydroxyphenyl)ethylidene)hydrazinyl)-3-oxo-1-(3,4,5-trimethoxyphenyl)prop-1-en-2-yl)-3,4,5-trimethoxybenzamide (**5d**)

White powder (0.32 g, 55.42%), m.p. 258–260 °C. ^1^H-NMR (400 MHz, DMSO-*d*_6_, δ ppm): 2.23 (s, 3H, CH_3_), 3.68 (s, 9H, 3OCH_3_), 3.74 (s, 3H, OCH_3_), 3.84 (s, 6H, 2OCH_3_), 6.77–6.88 (m, 2H, arom.CH), 7.03 (s, 2H, arom.CH), 7.22 (s, 1H, olefinic CH), 7.42 (s, 2H, arom.CH), 7.70 (d, *J* = 6.2 Hz, 2H, arom.CH), 9.80 (s, 1H, OH), 10.05 (s, 1H, NH), 10.43 (s, 1H, NH). ^13^C-NMR (100 MHz, DMSO-*d*_6_, δ ppm): 14.85 (CH_3_), 56.13 (2OCH_3_), 56.54 (2OCH_3_), 60.56 (OCH_3_), 60.62 (OCH_3_), 105.86 (C2,6 trimethoxyphenyl), 107.79 (C2,6 trimethoxybenzamide), 115.51 (C3,5 hydroxyphenyl), 115.60 (C1 hydroxyphenyl), 128.50 (C2,6 hydroxyphenyl), 128.94 (C olefinic), 129.28 (C1 trimethoxyphenyl), 129.96 (C olefinic), 131.16 (C1 trimethoxybenzamide), 138.54 (C4 trimethoxyphenyl), 140.96 (C4 trimethoxybenzamide), 146.58 (C=N), 153.05 (C3,5 trimethoxyphenyl and C3,5 trimethoxybenzamide), 159.38 (C4 hydroxyphenyl), 162.12 (C=O trimethoxybenzamide), 166.02 (C=O hydrazide). Anal. Calcd. for C_30_H_33_N_3_O_9_ (579.60): C, 62.17; H, 5.74; N, 7.25. Found: C, 62.31; H, 5.96; N, 6.99.

##### 3,4,5-Trimethoxy-N-((Z)-3-((E)-2-(1-(4-methoxyphenyl)ethylidene)hydrazinyl)-3-oxo-1-(3,4,5-trimethoxyphenyl)prop-1-en-2-yl)benzamide (**5e**)

White powder (0.37 g, 62.58%), m.p. 241–243 °C. ^1^H-NMR (400 MHz, DMSO-*d*_6_, δ ppm): 2.26 (s, 3H, CH_3_), 3.68 (s, 9H, 3OCH_3_), 3.74 (s, 3H, OCH_3_), 3.81 (s, 3H, OCH_3_), 3.84 (s, 6H, 2OCH_3_), 6.91–7.09 (m, 4H, arom.CH), 7.22 (s, 1H, olefinic CH), 7.42 (s, 2H, arom.CH), 7.83 (d, *J* = 10.4 Hz, 2H, arom.CH), 10.06 (s, 1H, OH), 10.49 (s, 1H, NH). ^13^C-NMR (100 MHz, DMSO-*d*_6_, δ ppm): 14.95 (CH_3_), 55.71 (OCH_3_), 56.14 (2OCH_3_), 56.54 (2OCH_3_), 60.57 (OCH_3_), 60.62 (OCH_3_), 105.86 (C2,6 trimethoxyphenyl), 107.81 (C2,6 trimethoxybenzamide), 114.13 (C3,5 methoxyphenyl), 127.95 (C2,6 methoxyphenyl), 128.40 (C olefinic), 128.53 (C1 trimethoxyphenyl), 128.92 (C olefinic), 129.96 (C1 trimethoxybenzamide), 130.86 (C1 methoxyphenyl), 138.55 (C4 trimethoxyphenyl), 140.96 (C4 trimethoxybenzamide), 146.20 (C=N), 153.06 (C3,5 trimethoxyphenyl), 153.08 (C3,5 trimethoxybenzamide), 158.92 (C4 methoxyphenyl), 162.33 (C=O trimethoxybenzamide), 169.79 (C=O hydrazide). Anal. Calcd. for C_31_H_35_N_3_O_9_ (593.62): C, 62.72; H, 5.94; N, 7.08. Found: C, 62.79; H, 5.97; N, 7.02.

##### N-((Z)-3-((E)-2-(1-(3,4-Dimethoxyphenyl)ethylidene)hydrazinyl)-3-oxo-1-(3,4,5-trimethoxyphenyl)prop-1-en-2-yl)-3,4,5-trimethoxybenzamide (**5f**)

White powder (0.44 g, 71.06%), m.p. 234–236 °C. ^1^H-NMR (400 MHz, DMSO-*d*_6_, δ ppm): 2.28 (s, 3H, CH_3_), 3.68 (s, 9H, 3OCH_3_), 3.74 (s, 3H, OCH_3_), 3.81 (s, 3H, OCH_3_), 3.84 (s, 9H, 3OCH_3_), 7.03 (s, 2H, arom.CH), 7.21 (s, 1H, olefinic CH), 7.32–7.54 (m, 4H, arom.CH), 7.92 (d, *J* = 7.4 Hz, 1H, arom.CH), 10.07 (s, 1H, NH), 10.53 (s, 1H, NH). ^13^C-NMR (100 MHz, DMSO-*d*_6_, δ ppm): 15.04 (CH_3_), 56.00 (2OCH_3_), 56.15 (2OCH_3_), 56.54 (2OCH_3_), 60.57 (OCH_3_), 60.63 (OCH_3_), 105.87 (C2,6 trimethoxyphenyl), 107.79 (C2,6 trimethoxybenzamide), 109.75 (C5 dimethoxyphenyl), 111.54 (C2 dimethoxyphenyl), 120.35 (C6 dimethoxyphenyl), 126.89 (C1 dimethoxyphenyl), 128.93 (C olefinic), 129.37 (C1 trimethoxyphenyl), 129.97 (C olefinic), 130.26 (C1 trimethoxybenzamide), 131.02 (), 138.55 (C4 trimethoxyphenyl), 140.96 (C4 trimethoxybenzamide), 148.90 (C=N), 150.81 (C3 dimethoxyphenyl), 153.06 (C3,5 trimethoxyphenyl), 153.07 (C3,5 trimethoxybenzamide), 156.90 (C4 dimethoxyphenyl), 162.31 (C=O trimethoxybenzamide), 166.02 (C=O hydrazide). Anal. Calcd. for C_32_H_37_N_3_O_10_ (623.65): C, 61.63; H, 5.98; N, 6.74. Found: C, 61.74; H, 6.11; N, 6.62.

##### 3,4,5-Trimethoxy-N-((Z)-3-oxo-1-(3,4,5-trimethoxyphenyl)-3-((E)-2-(1-(3,4,5-trimethoxyphenyl)ethylidene)hydrazinyl)prop-1-en-2-yl)benzamide (**5g**)

White powder (0.36 g, 55.19%), m.p. 211–213 °C. ^1^H-NMR (400 MHz, DMSO-*d*_6_, δ ppm): 2.31 (s, 3H, CH_3_), 3.69 (s, 12H, 4OCH_3_), 3.74 (s, 3H, OCH_3_), 3.84 (s, 12H, 4OCH_3_), 7.04 (s, 2H, arom.CH), 7.13 (s, 2H, arom.CH), 7.20 (s, 1H, olefinic CH), 7.42 (s, 2H, arom.CH), 10.08 (s, 1H, NH), 10.60 (s, 1H, NH). ^13^C-NMR (100 MHz, DMSO-*d*_6_, δ ppm): 15.31 (CH_3_), 56.16 (2OCH_3_), 56.30 (OCH_3_), 56.42 (OCH_3_), 56.53 (2OCH_3_), 60.58 (2OCH_3_), 60.62 (OCH_3_), 104.54 (C2,6 trimethoxyphenyl), 105.86 (C2,6 trimethoxyphenylethylidene), 107.80 (C2,6 trimethoxyphenyl), 123.94 (C1 trimethoxyphenylethylidene), 128.90 (C olefinic), 129.29 (C1 trimethoxyphenyl), 129.94 (C olefinic), 130.26 (C1 trimethoxybenzamide), 136.66 (C4 trimethoxyphenylethylidene), 138.55 (C4 trimethoxyphenyl), 140.97 (C4 trimethoxybenzamide), 145.37 (C=N), 153.07 (C3,5 trimethoxyphenylethylidene), 153.08 (C3,5 trimethoxyphenyl and C3,5 trimethoxybenzamide), 165.92 (C=O trimethoxybenzamide), 170.63 (C=O hydrazide). Anal. Calcd. for C_33_H_39_N_3_O_11_ (653.68): C, 60.63; H, 6.01; N, 6.43. Found: C, 60.47; H, 5.86; N, 6.61.

### 3.3. Biological Assays

#### 3.3.1. Cytotoxic Activity against MDA-MB-231 Cell Line

Cytotoxic activity of the prepared schiff bases against MDA-MB-231 breast cell Line was determined according previously reported methods [45]. (See Supplementary Materials—Appendix SB).

#### 3.3.2. Tubulin Inhibition Assays

Compound **4h** was evaluated for their Tubulin inhibitory activity according to manufacturer’s instructions [38].

#### 3.3.3. Cell Cycle Analysis of Compound 4h

See Supplementary Materials—Appendix SB.

#### 3.3.4. Annexin V FITC/PI Staining Assay for Compound 4h

See Supplementary Materials—Appendix SB.

#### 3.3.5. Mitochondrial Membrane Potential

See Supplementary Materials—Appendix SB.

#### 3.3.6. ELISA Measurements of p53, Bax and Bcl-2

See Supplementary Materials—Appendix SB.

#### 3.3.7. Molecular Docking Study

See Supplementary Materials—Appendix SB.

### 3.4. Fabrication of Compound 4h Loaded NLCs

Briefly, the organic phase composed of lipid either Precirol ATO-5 or Geleol in either 2% or 6% along with Capryol 90 were dispersed in THF along with **4h** which was dripped slowly to an aqueous phase composing either 1 or 2% of a surfactant (Cremophor RH40) under stirring. The attained primary O/W emulsion was introduced to ultrasonication using a probe sonicator for 10 min at 60% amplitude (Model 275 T Crest Ultrasonics Corp., Trenton, NJ, USA). Finally, the resulting emulsion was kept under continuous stirring (MS-300HS, Misung Scientific Co., Korea) to permit the complete evaporation of the organic solvent and the precipitation of **4h**-NLCs [39].

## 4. Conclusions

**Finally:** new Schiff base-based TMP compounds were synthesized and evaluated for their cytotoxic activity against the breast MDA-MB-231 cell line and the normal breast MCF-10A to conclude this research. The tested Schiff bases revealed good activity over the MDA-MB-231 cell line, especially compounds **4h**, **4j** and **5d** with IC_50_ values 1.27 ± 0.18 μM, 2.84 ± 0.18 μM and 1.98 ± 0.19 μM, respectively, compared to CA-4 (IC_50_ = 0.54 ± 0.04). The cytotoxic activity of Schiff base **4h** is correlated to tubulin polymerization inhibitory activity as revealed by immunofluorescence analysis and β-tubulin polymerization inhibition percentage on MDA-MB-231 cells (78.14% polymerization inhibition at 1.27 μM). In addition, compound **4h** caused cell cycle arrest at the G2/M phase (fivefold more than control MDA-MB-231 cells) and cellular apoptosis as ascertained by an increase in the percentage of the pre-G1 phase by almost 19-fold compared with negative control cells and Annexin V FITC/PI staining assay. Moreover, compound **4h** was found to be an apoptotic inducer via a decrease in the level of MMP and Bcl-2 by 3- and 8-fold, respectively, compared to negative control cells and an increase in the level of p53 and Bax by 11- and 5-fold, respectively, compared to the negative control cells. Additionally, the most potent compound **4h** was lodged on nanostructured lipid carriers (NLCs). Eight formulae resulted from 2^3^ full factorial design which was conducted to govern the influence of the fabrication variables on the in vitro characters of the casted NLCs. Based on the outcomes of the experimental design and factorial analysis, F3 was picked as the optimum formula exhibiting a dominant desirability value of 0.805, EE% 95.6 ± 2.4, PS 222.4 ± 18.7, PDI 0.23 ± 0.05 and ZP −39.2 ± 3.9 Mv. Moreover, F3 exhibited an improved release and solubility profile over that of the drug suspension. In the comparative cytotoxic activity, F3 was capable of diminishing the IC_50_ by around 2.15 times for pure **4h**, while nearly close to the IC_50_ of the reference drug. Thus, NLCs could be a potential platform for boosted antitumor activity of compound **4h** against MDA-MB-231 cancer cells.

## Figures and Tables

**Table 1:** Cytotoxic screening of the tested Schiff bases **2**–**5g**. Values are the mean of three independent replicates ± SE.

Compound No.	IC_50_ Value (μM)	
	MDA-MB-231	MCF-10A
**2**	31.59 ± 1.07	NT
**3**	6.08 ± 0.42	NT
**4a**	23.70 ± 1.13	NT
**4b**	16.10 ± 0.83	NT
**4c**	38.82 ± 0.93	NT
**4d**	5.98 ± 0.28	NT
**4e**	7.10 ± 1.02	NT
**4f**	4.52 ± 0.36	NT
**4g**	3.43 ± 0.22	NT
**4h**	1.27 ± 0.18	30.83 ± 2.32
**4i**	3.46 ± 0.51	NT
**4j**	2.84 ± 0.18	NT
**4k**	4.80 ± 0.17	NT
**4l**	7.30 ± 0.64	NT
**5a**	20.15 ± 0.89	NT
**5b**	13.98 ± 1.19	NT
**5c**	20.73 ± 1.84	NT
**5d**	1.98 ± 0.19	NT
**5e**	9.52 ± 1.18	NT
**5f**	8.34 ± 1.22	NT
**5g**	3.39 ± 0.26	NT
CA-4	0.54 ± 0.04	8.86 ± 0.67

**Table 2:** Experimental runs, independent variables, and estimated responses of **4h**-loaded NLCs via 2^3^ full factorial experimental.

Formula	A (Lipid Type)	B (Lipid Conc. %*w*/*v*)	C (Surfactant Conc. %*w*/*v*)	Y1 (EE%)	Y2 (PS)	Y3 (PDI)	Y4 (ZP)
**F1**	Precirol	2	1	80.9 ± 3.5	142.5 ± 12.2	0.34 ± 0.04	−24.6 ± 4.1
**F2**	Geleol	2	1	51.2 ± 2.8	311.2 ± 19.1	0.31 ± 0.05	−16.4 ± 1.2
**F3**	Precirol	6	1	95.6 ± 4.1	222.4 ± 11.7	0.23 ± 0.06	−39.2 ± 3.4
**F4**	Geleol	6	1	83.7 ± 4.6	354.3 ± 17.3	0.27 ± 0.05	−29.8 ± 2.2
**F5**	Precirol	2	2	54.3 ± 1.9	100.7 ± 8.1	0.54 ± 0.08	−18.3 ± 1.9
**F6**	Geleol	2	2	37.4 ± 1.8	253.4 ± 9.5	0.64 ± 0.17	−12.9 ± 1.8
**F7**	Precirol	6	2	86.8 ± 3.3	189.7 ± 12.6	0.36 ± 0.05	−31.2 ± 3.3
**F8**	Geleol	6	2	67.2 ± 2.4	269.5 ± 23.2	0.28 ± 0.02	21.7 ± 2.5

**Table 3:** 2^3^ factorial investigation outcome of **4h**-loaded NLC and the predicted, observed responses and deviation percent of the Optimum formula (F3).

Responses	EE(%)	PS (nm)	ZP (Mv)
**R^2^**	0.961	0.954	0.981
**Adjusted R^2^**	0.93	0.92	0.966
**Predicted R^2^**	0.845	0.818	0.92
**Adequate precision**	16.9	14.5	23.6
**Significant factors**	A, B, C	A, B, C	A, B, C
**Observed value of the optimal formula (F3)**	95.6	222.4	−39.2
**Predicted value of the optimal formula (F3)**	95.7	200.6	−35.5
**Absolute deviation %**	0.1	9.8	9.4

## Data Availability

Data is contained within the article and supplementary material.

## Supplementary Materials

The following supporting information can be downloaded at: www.mdpi.com/article/10.3390/ph15060679/s1, Figure S1: ^1^H-NMR spectrum of compound **2**, Figure S2: ^13^C-NMR spectrum of compound **2**, Figure S3: ^1^H-NMR spectrum of compound **3**, Figure S4: ^13^C-NMR spectrum of compound **3,** Figure S5: ^1^H-NMR spectrum of compound **4a**, Figure S6: ^13^C-NMR spectrum of compound **4a,** Figure S7: ^1^H-NMR spectrum of compound **4b**, Figure S8: ^13^C-NMR spectrum of compound **4b**, Figure S9: ^1^H-NMR spectrum of compound **4c**, Figure S10: ^13^C-NMR spectrum of compound **4c**, Figure S11: ^1^H-NMR spectrum of compound **4d**, Figure S12: ^13^C-NMR spectrum of compound **4d**, Figure S13: ^1^H-NMR spectrum of compound **4e**, Figure S14: ^13^C-NMR spectrum of compound **4e**, Figure S15: ^1^H-NMR spectrum of compound **4f**, Figure S16: ^13^C-NMR spectrum of compound **4f**, Figure S17: ^1^H-NMR spectrum of compound **4g**, Figure S18: ^13^C-NMR spectrum of compound **4g,** Figure S19: ^1^H-NMR spectrum of compound **4h**, Figure S20: ^13^C-NMR spectrum of compound **4h**, Figure S21: ^1^H-NMR spectrum of compound **4i**, Figure S22: ^13^C-NMR spectrum of compound **4i**, Figure S23: ^1^H-NMR spectrum of compound **4j**, Figure S24: ^13^C-NMR spectrum of compound **4j**, Figure S25: ^1^H-NMR spectrum of compound **4k**, Figure S26: ^13^C-NMR spectrum of compound **4k**, Figure S27: ^1^H-NMR spectrum of compound **4l**, Figure S28: ^13^C-NMR spectrum of compound **4l**, Figure S29: ^1^H-NMR spectrum of compound **5a**, Figure S30: ^13^C-NMR spectrum of compound **5a**, Figure S31: ^1^H-NMR spectrum of compound **5b,** Figure S32: ^13^C-NMR spectrum of compound **5b**, Figure S33: ^1^H-NMR spectrum of compound **5c**, Figure S34: ^13^C-NMR spectrum of compound **5c**, Figure S35: ^1^H-NMR spectrum of compound **5d**, Figure S36: ^13^C-NMR spectrum of compound **5d**, Figure S37: ^1^H-NMR spectrum of compound **5e**, Figure S38: ^13^C-NMR spectrum of compound **5e**, Figure S39: ^1^H-NMR spectrum of compound **5f**, Figure S40: ^13^C-NMR spectrum of compound **5f**, Figure S41: ^1^H-NMR spectrum of compound **5g**, Figure S42: ^13^C-NMR spectrum of compound **5g**, Appendix SA and Appenidx SB.
