# Supplementary material for: Design, Synthesis, In Vitro Biological Activity Evaluation and Stabilized Nanostructured Lipid Carrier Formulation of Newly Synthesized Schiff Bases-Based TMP Moieties
Source: Pharmaceuticals (Basel). 2022 May 28;15(6):679. doi: 10.3390/ph15060679 (PMC9230623; doi:10.3390/ph15060679)
Supplement: Supplementary file 1 [file pharmaceuticals-15-00679-s001.zip › pharmaceuticals-1710368-supplementary.pdf]

Supporting Information

Design, Synthesis, *In Vitro* Biological Activity Evaluation and Stabilized Nanostructured Lipid Carrier Formulation of Newly Synthesized Schiff Bases Based TMP Moieties

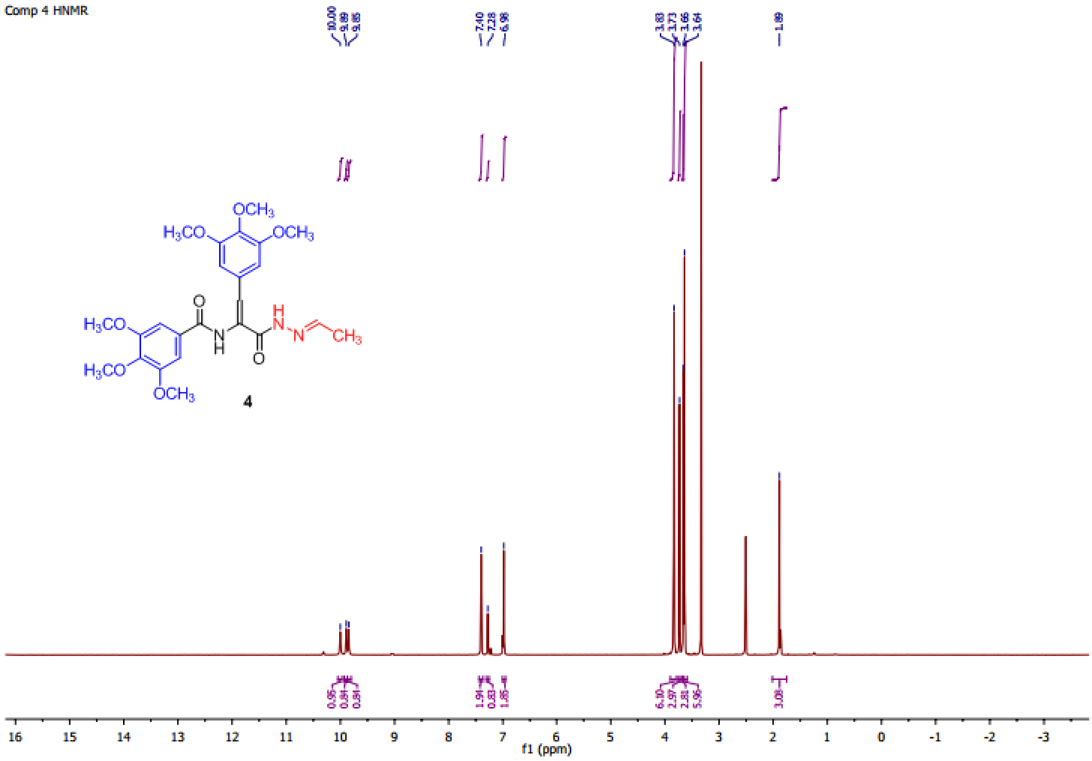

Figure S1: ^{1}H-NMR spectrum of compound **2**

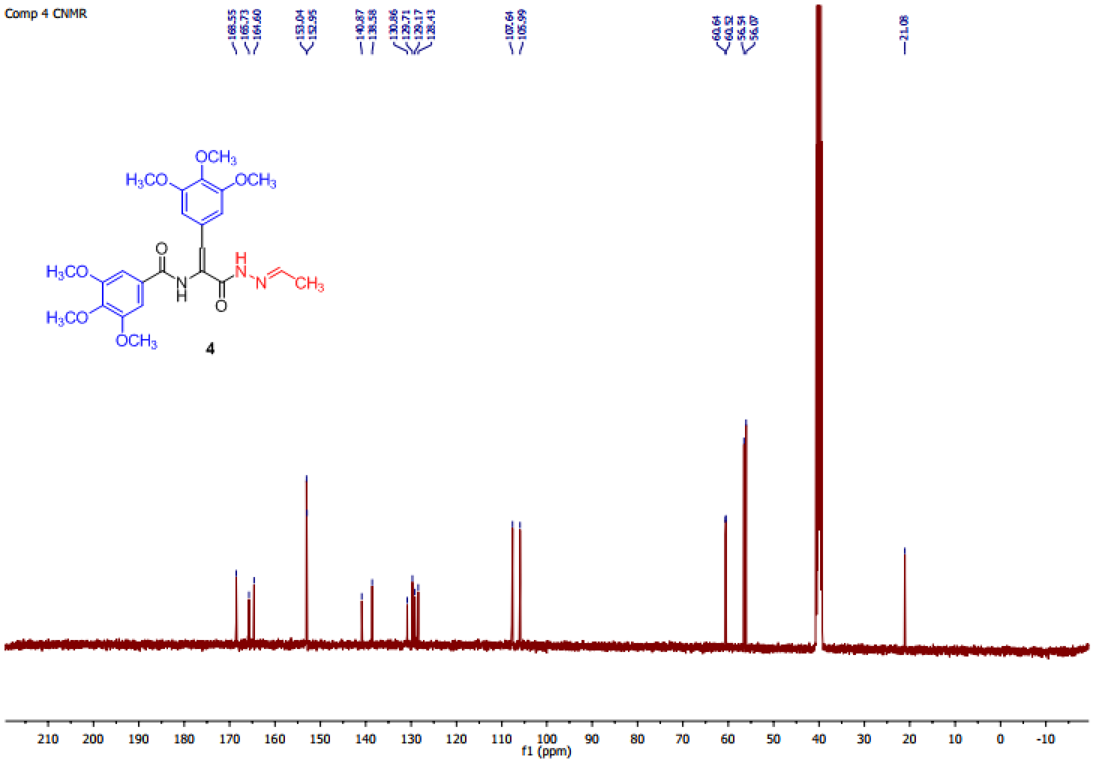

Figure S2: ^{13}C-NMR spectrum of compound **2**

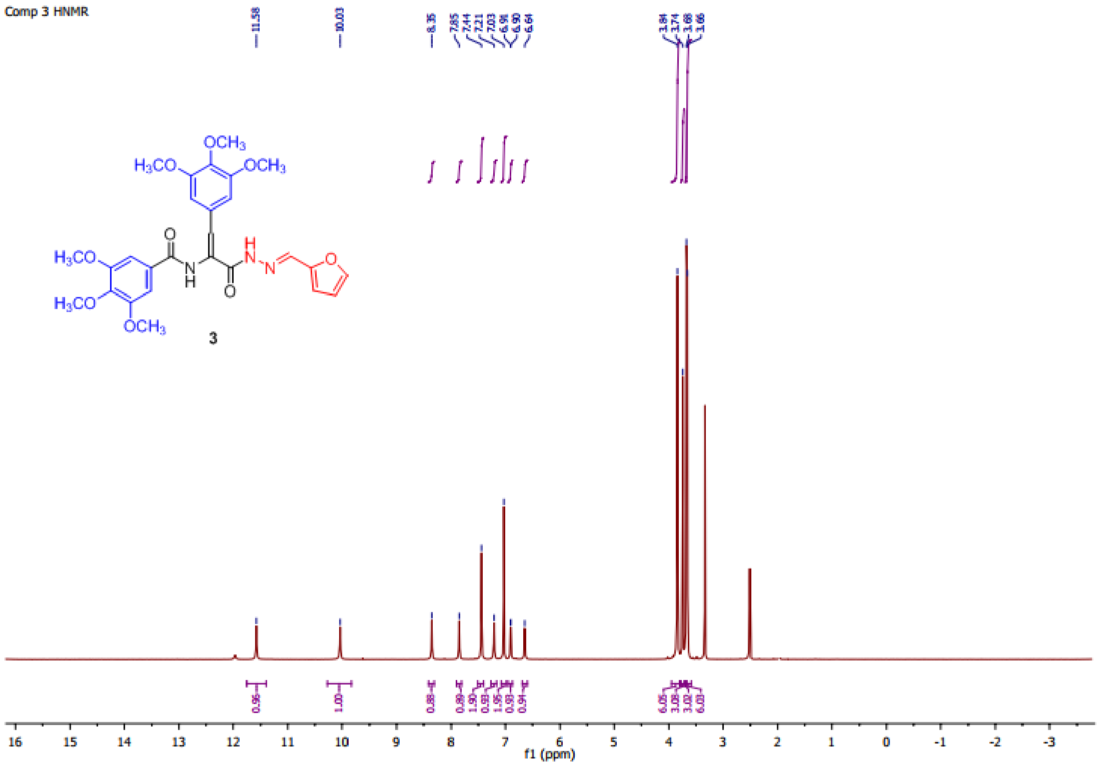

Figure S3: ^{1}H-NMR spectrum of compound **3**

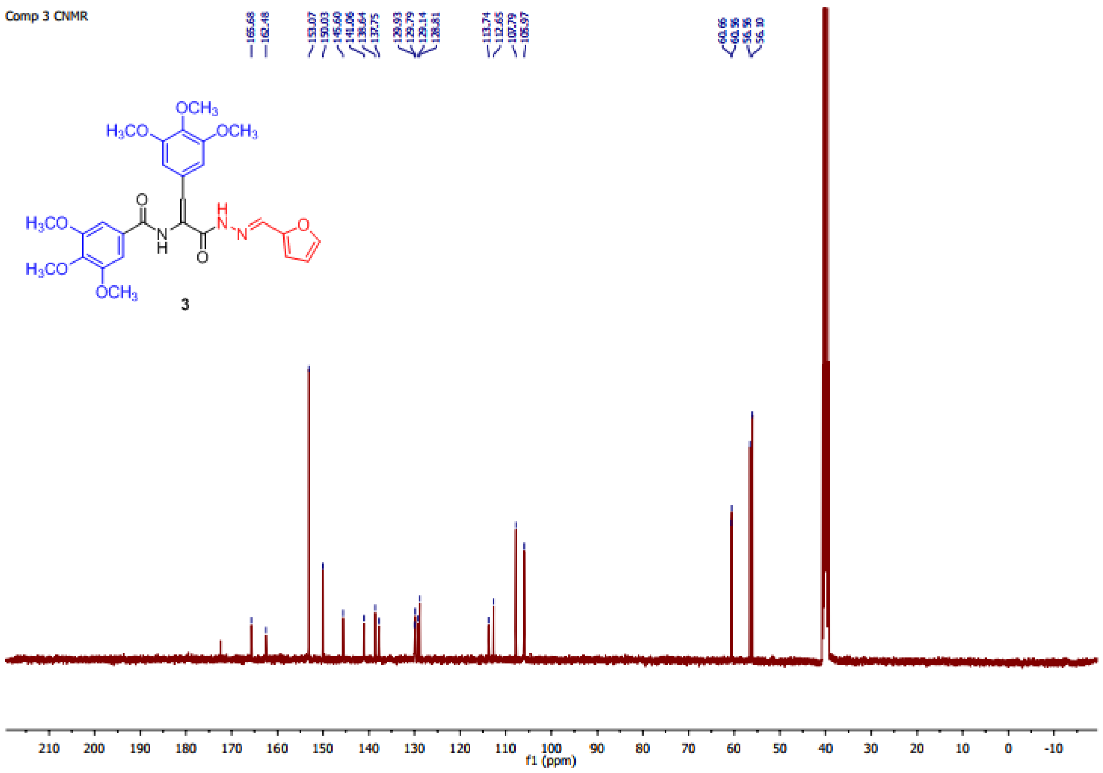

Figure S4: ^{13}C-NMR spectrum of compound **3**

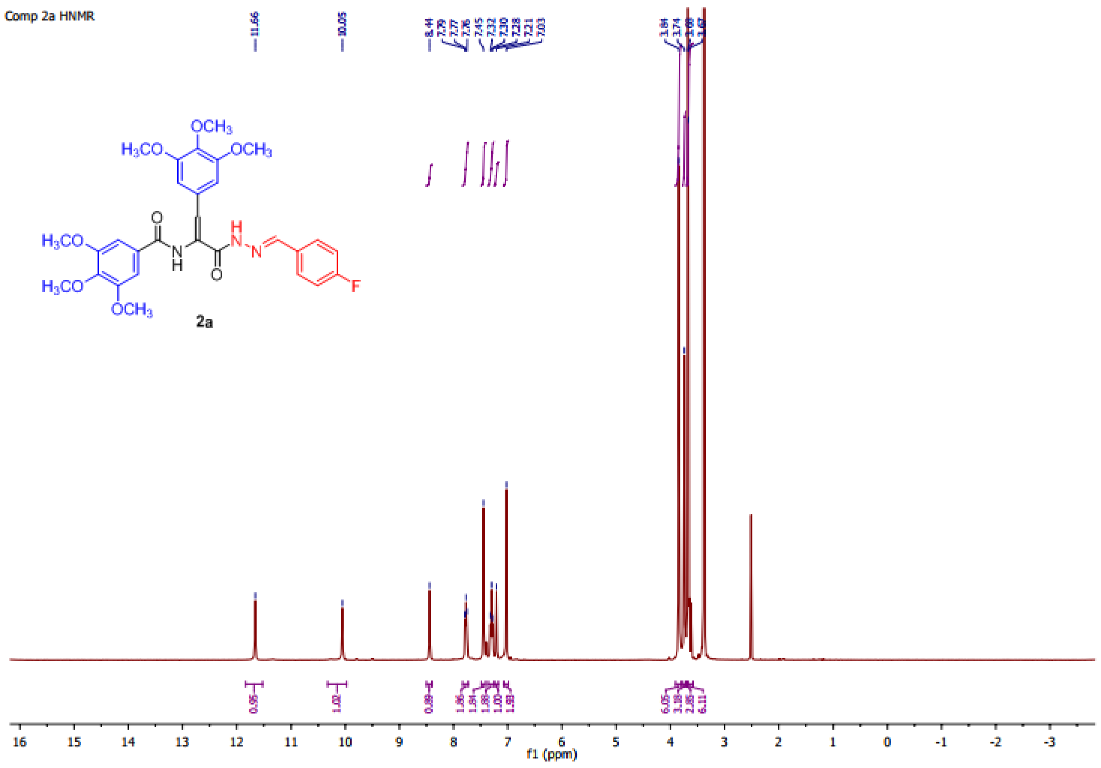

Figure S5: ^{1}H-NMR spectrum of compound **4a**

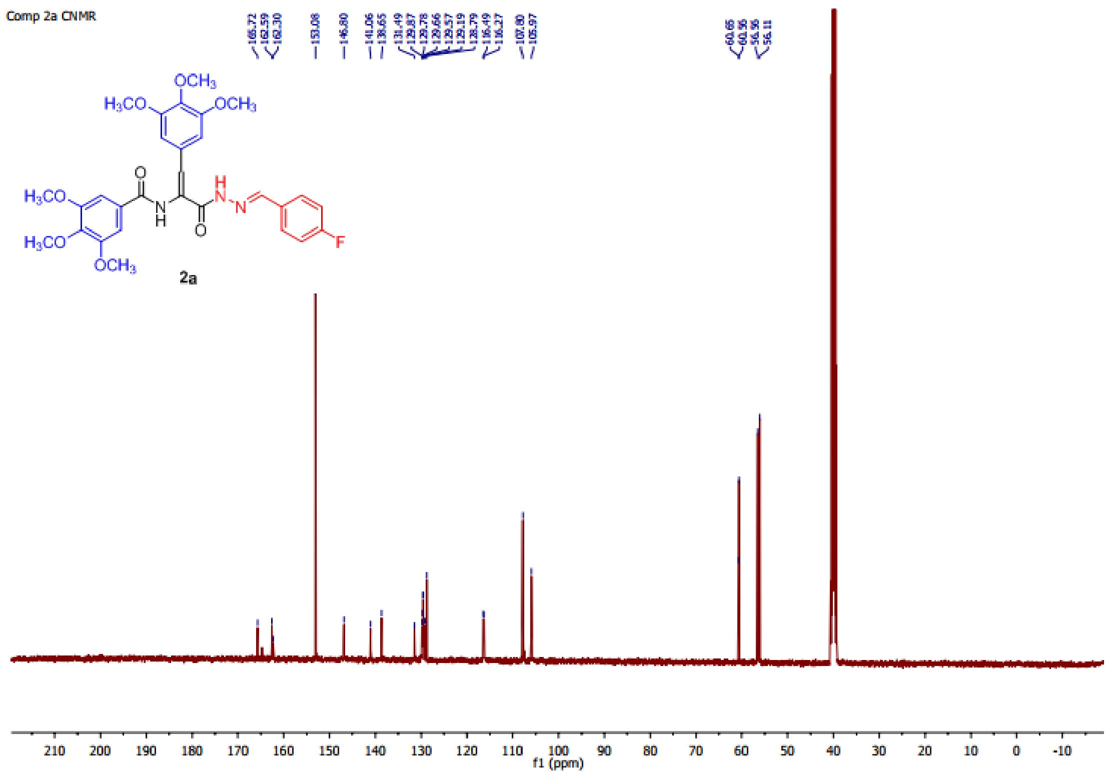

Figure S6: ^{13}C-NMR spectrum of compound **4a**

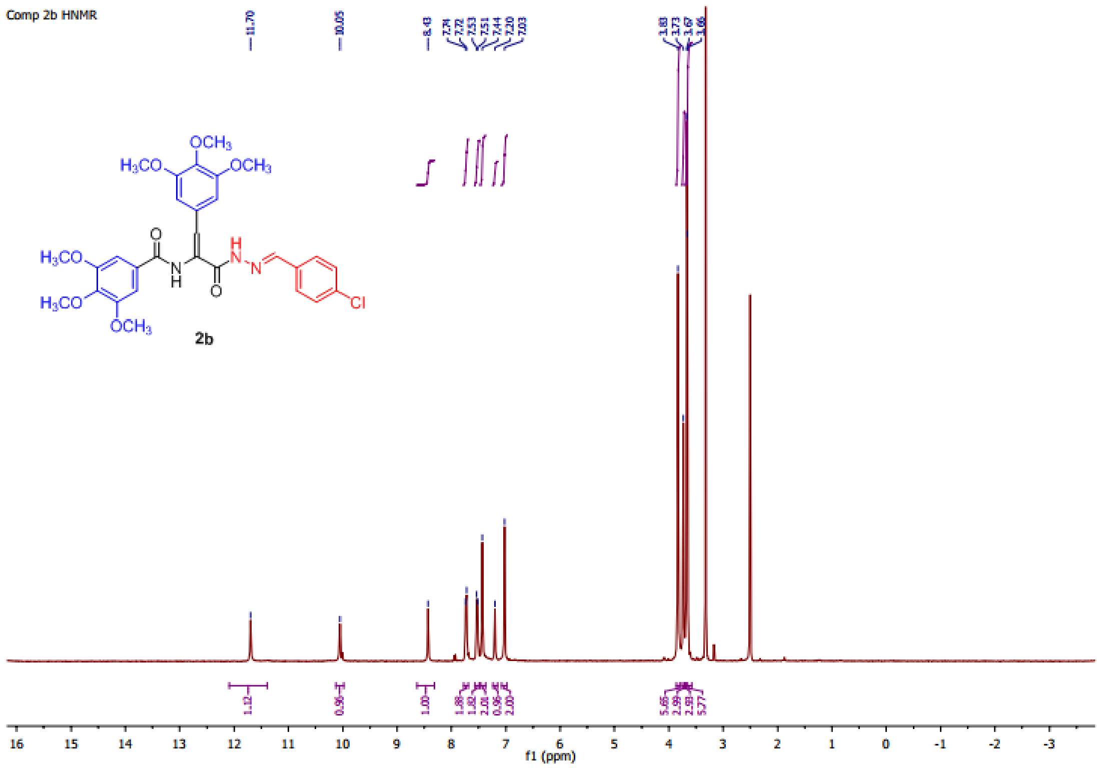

Figure S7: ^{1}H-NMR spectrum of compound **4b**

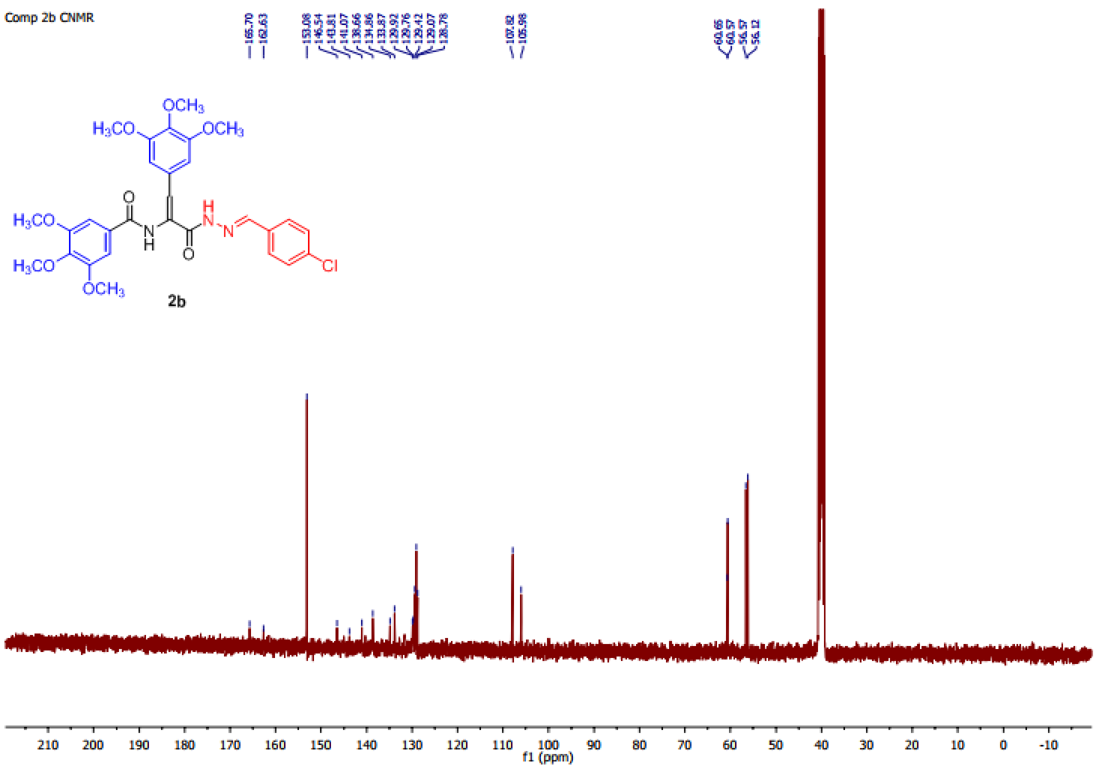

Figure S8: ^{13}C-NMR spectrum of compound **4b**

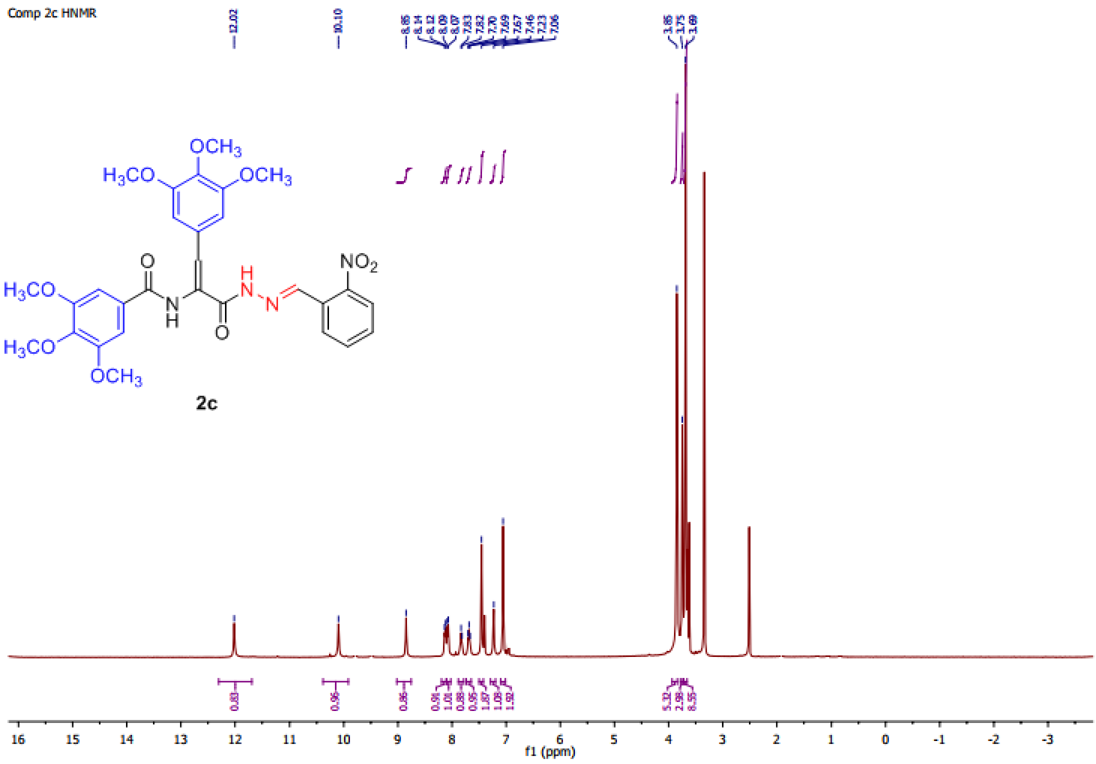

Figure S9: ^{1}H-NMR spectrum of compound **4c**

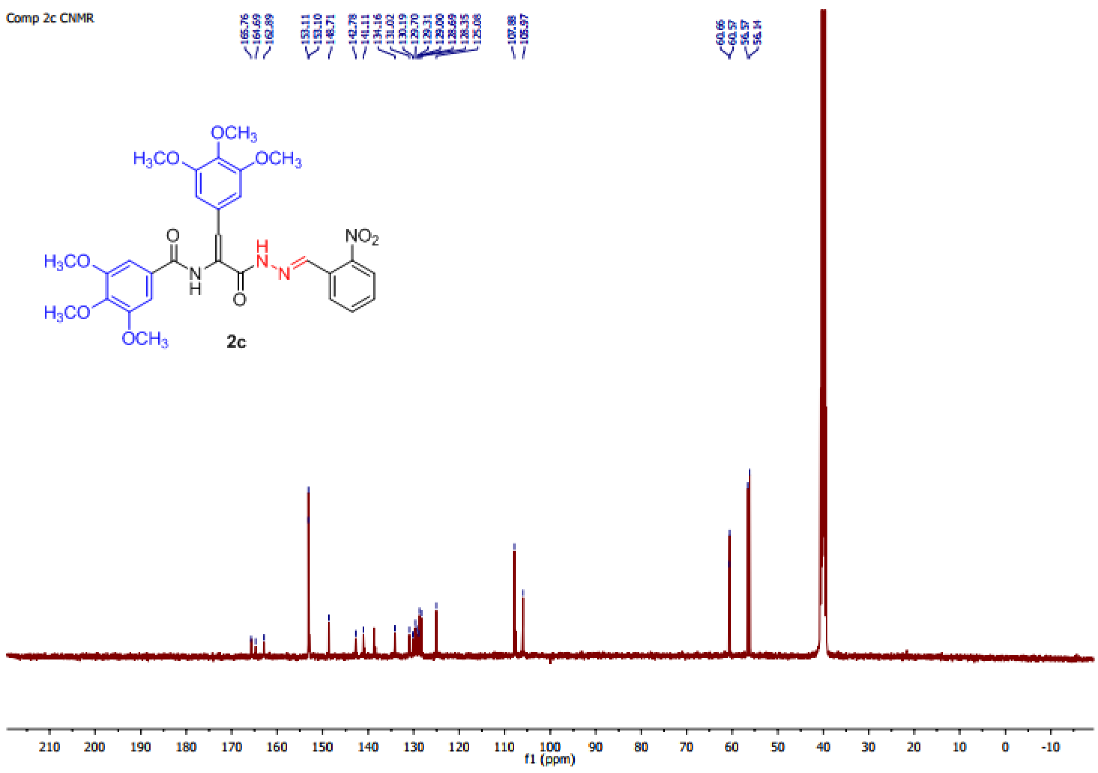

Figure S10: ^{13}C-NMR spectrum of compound **4c**

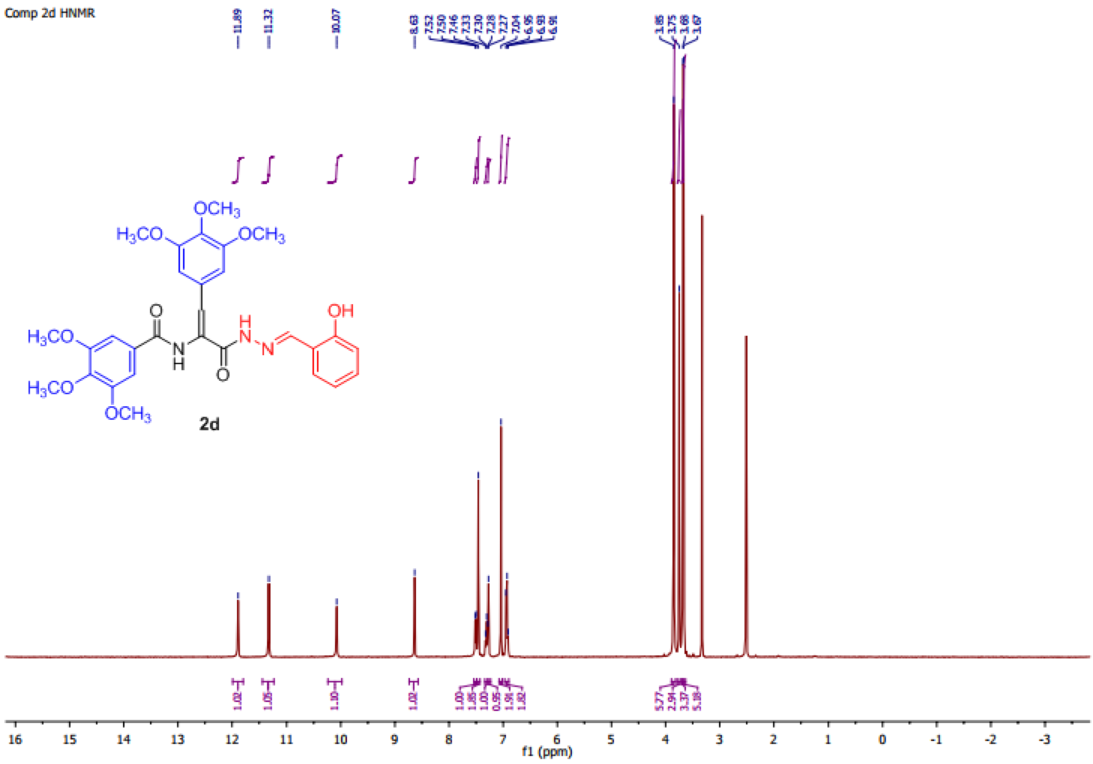

Figure S11: ^{1}H-NMR spectrum of compound **4d**

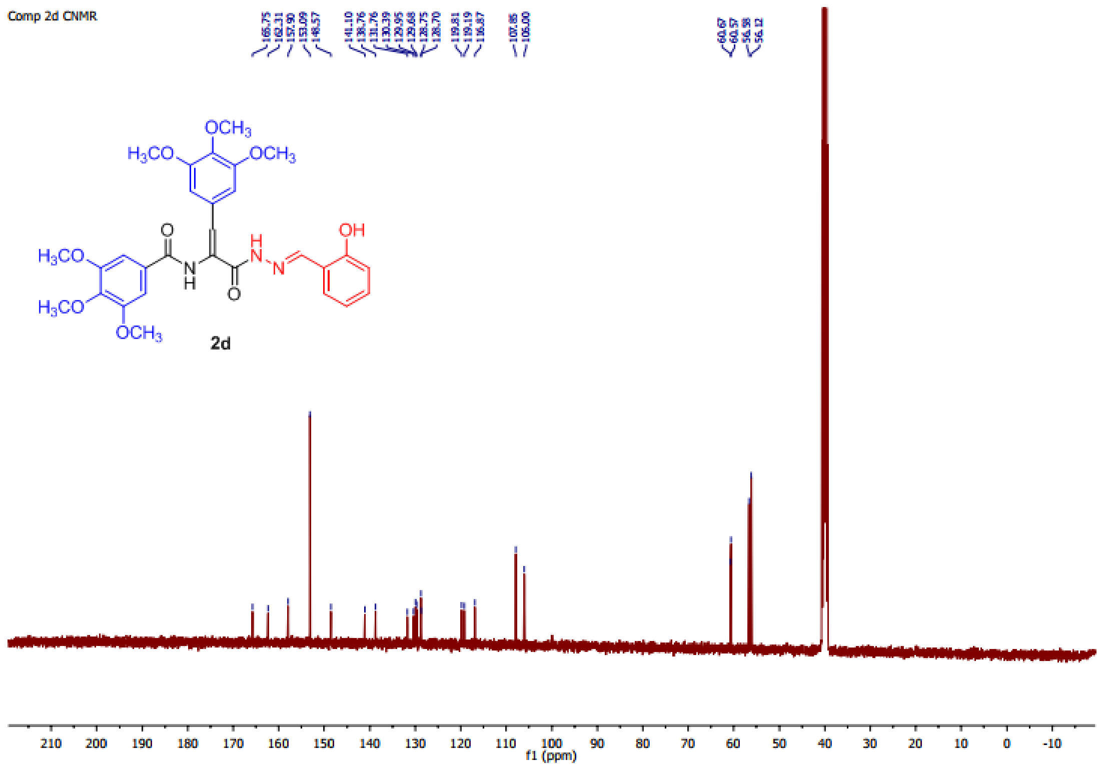

Figure S12: ^{13}C-NMR spectrum of compound **4d**

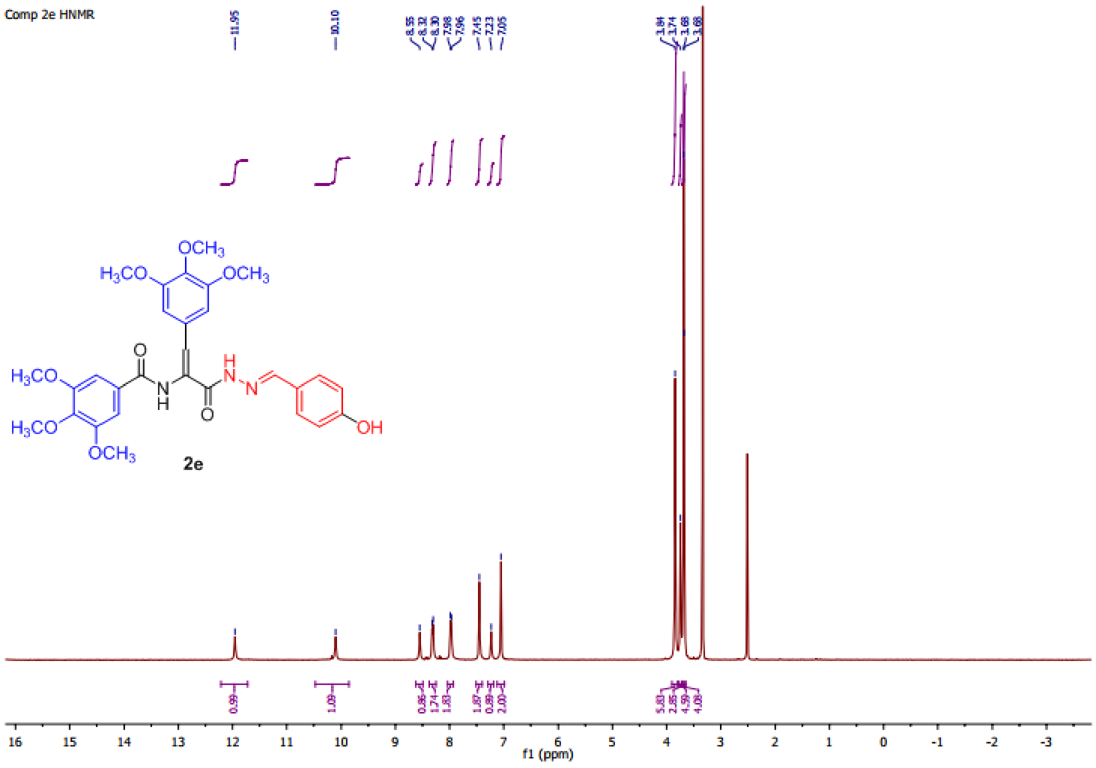

Figure S13: ^{1}H-NMR spectrum of compound **4e**

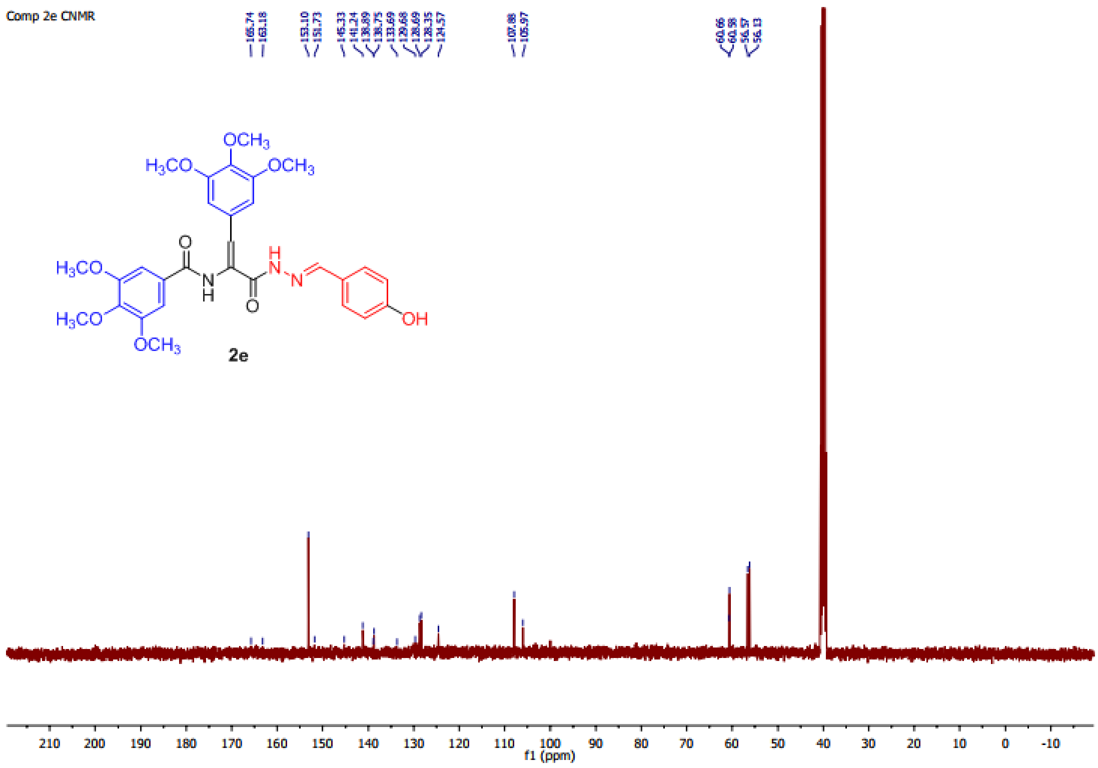

Figure S14: ^{13}C-NMR spectrum of compound **4e**

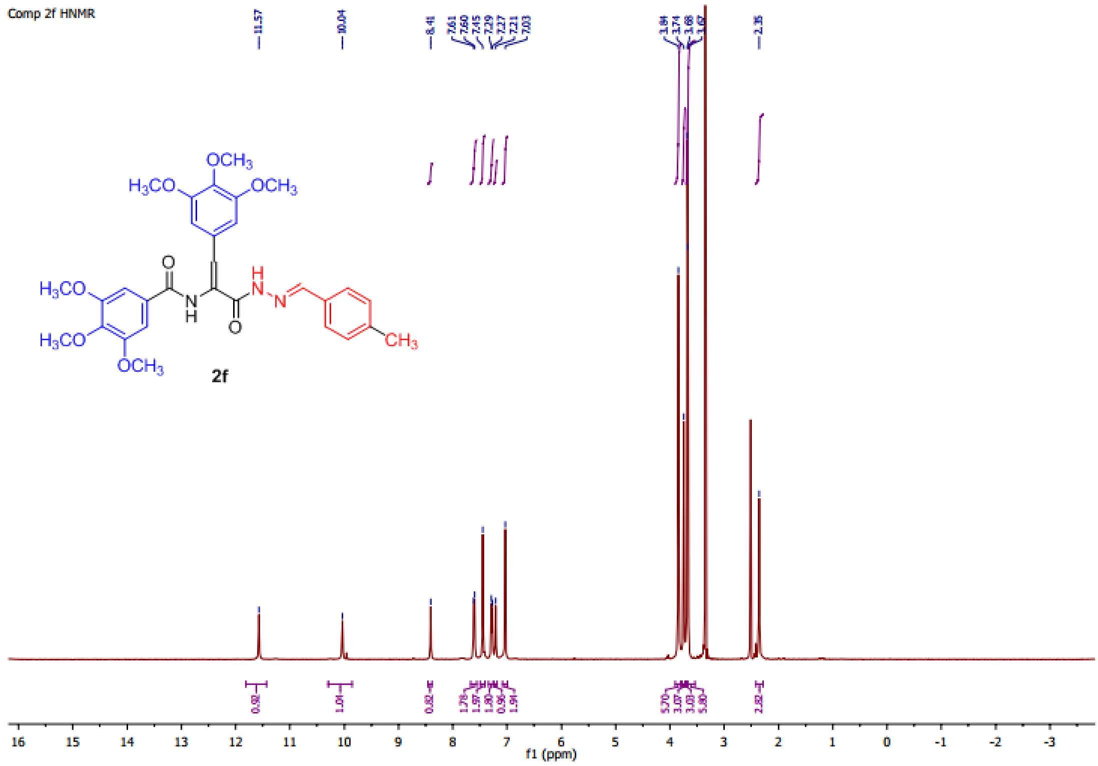

Figure S15: ^{1}H-NMR spectrum of compound **4f**

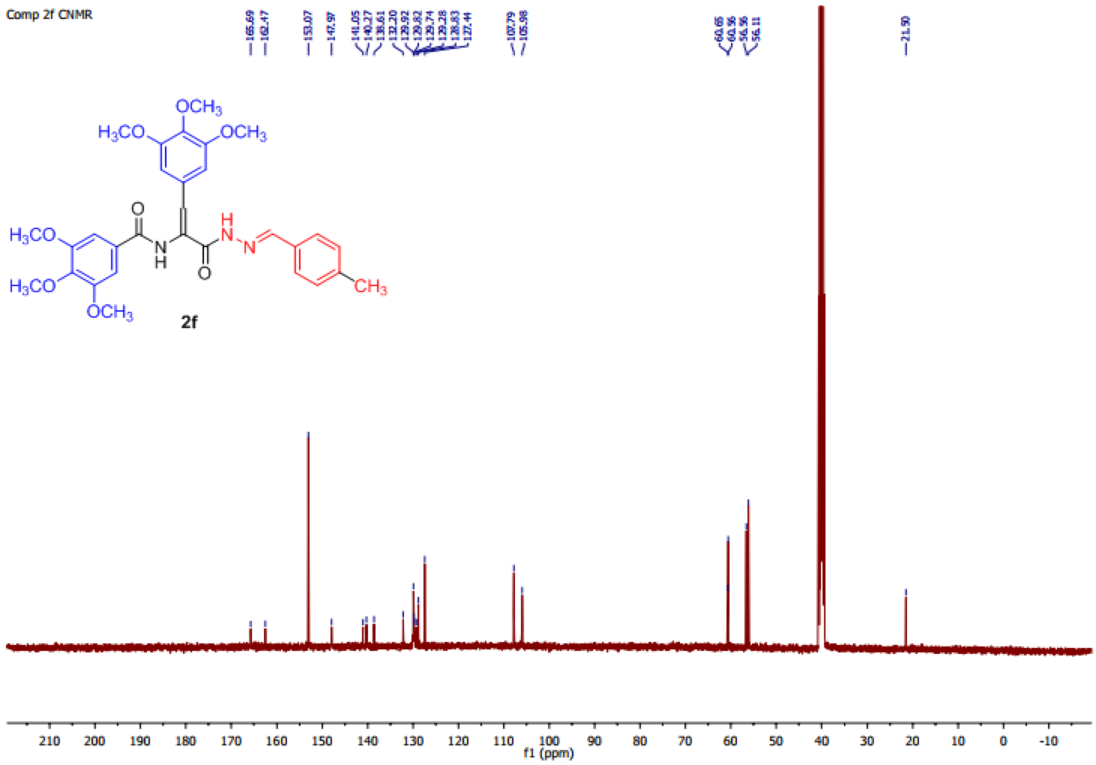

Figure S16: ^{13}C-NMR spectrum of compound **4f**

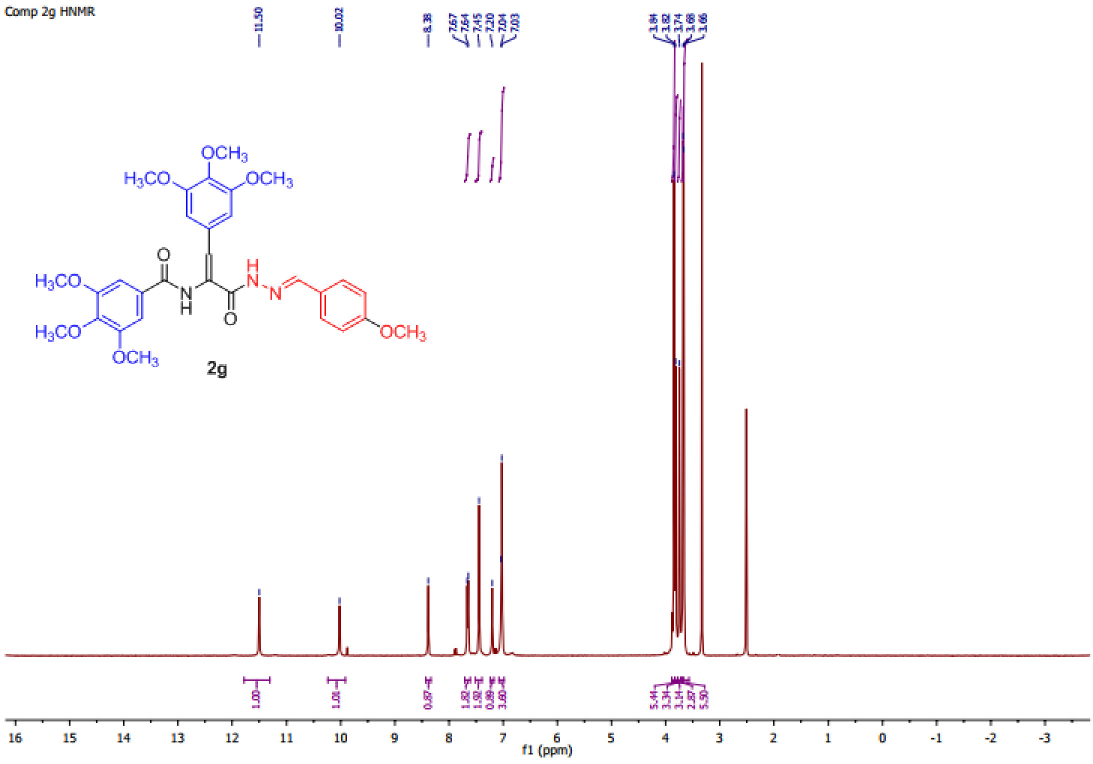

Figure S17: ^{1}H-NMR spectrum of compound **4g**

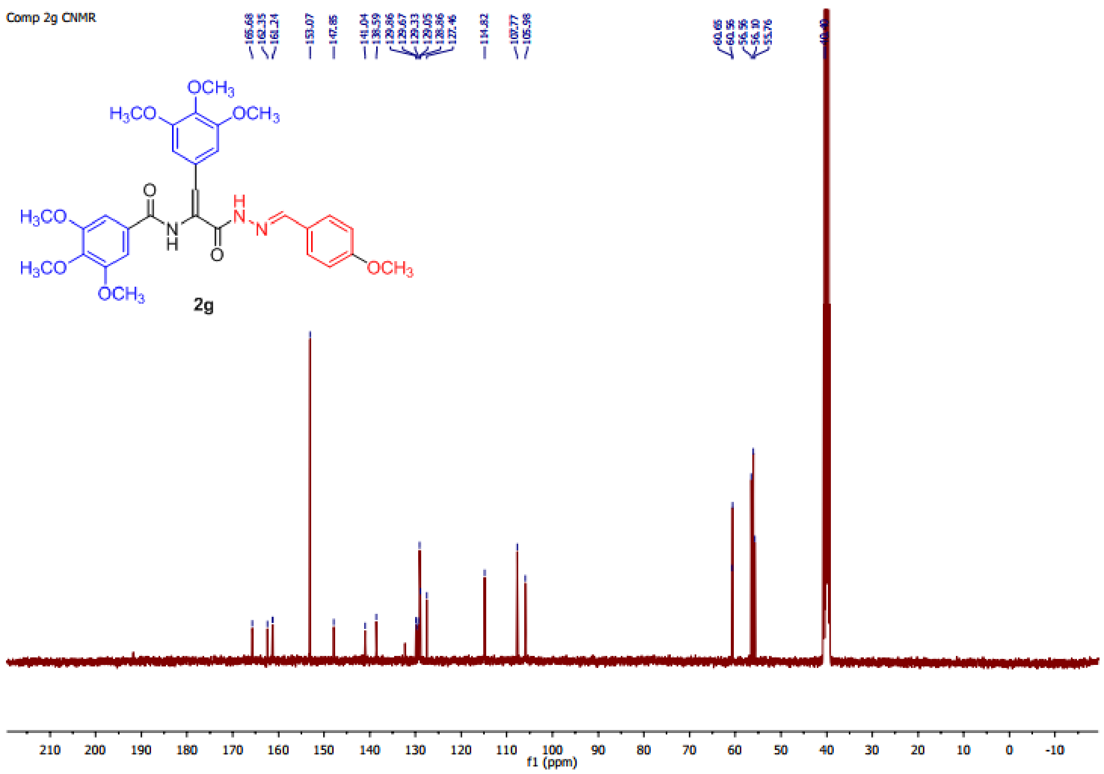

Figure S18: ^{13}C-NMR spectrum of compound **4g**

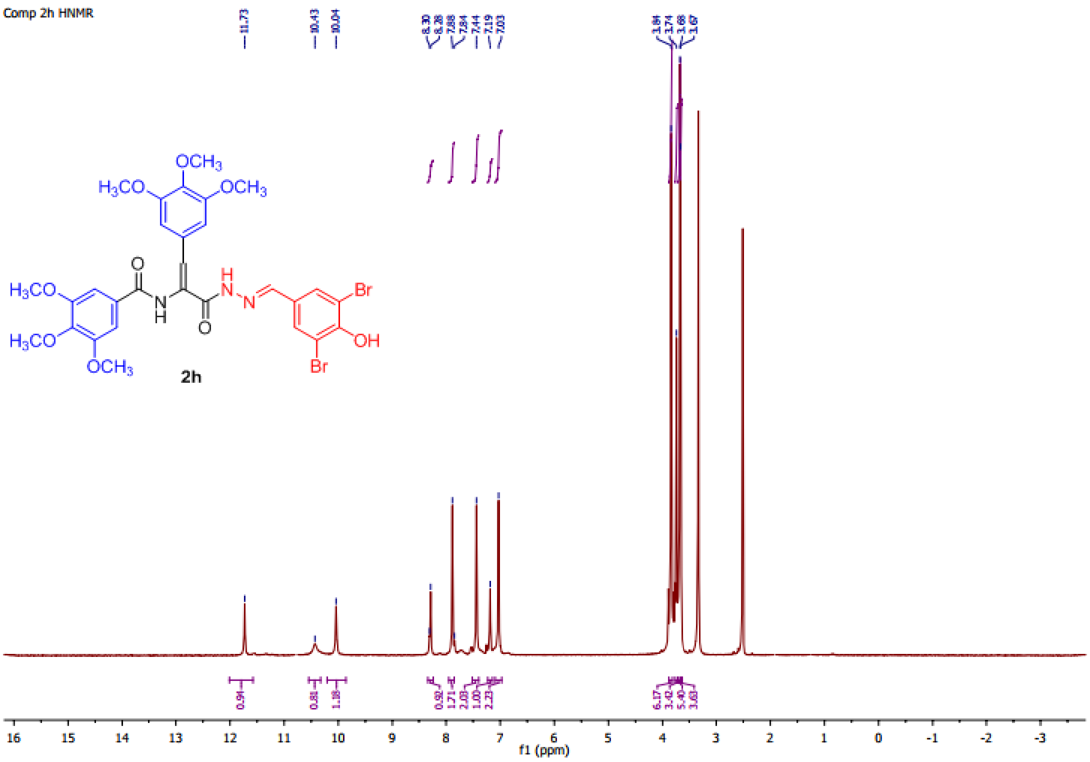

Figure S19: ^{1}H-NMR spectrum of compound **4h**

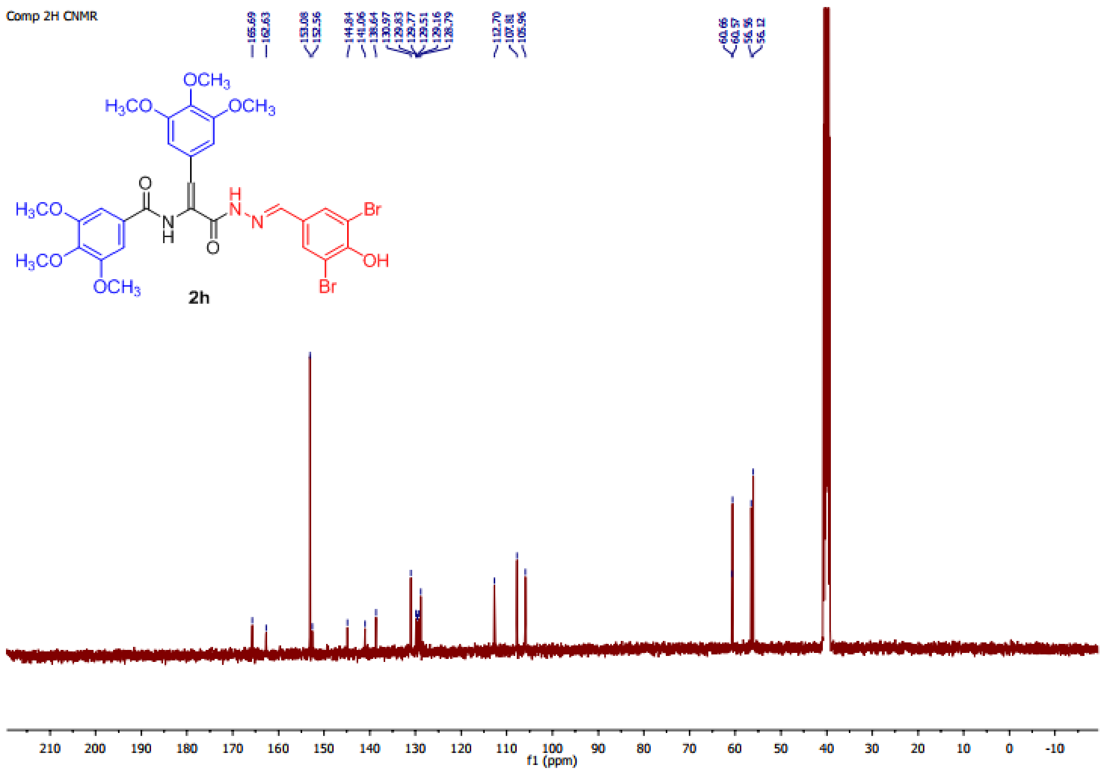

Figure S20: ^{13}C-NMR spectrum of compound **4h**

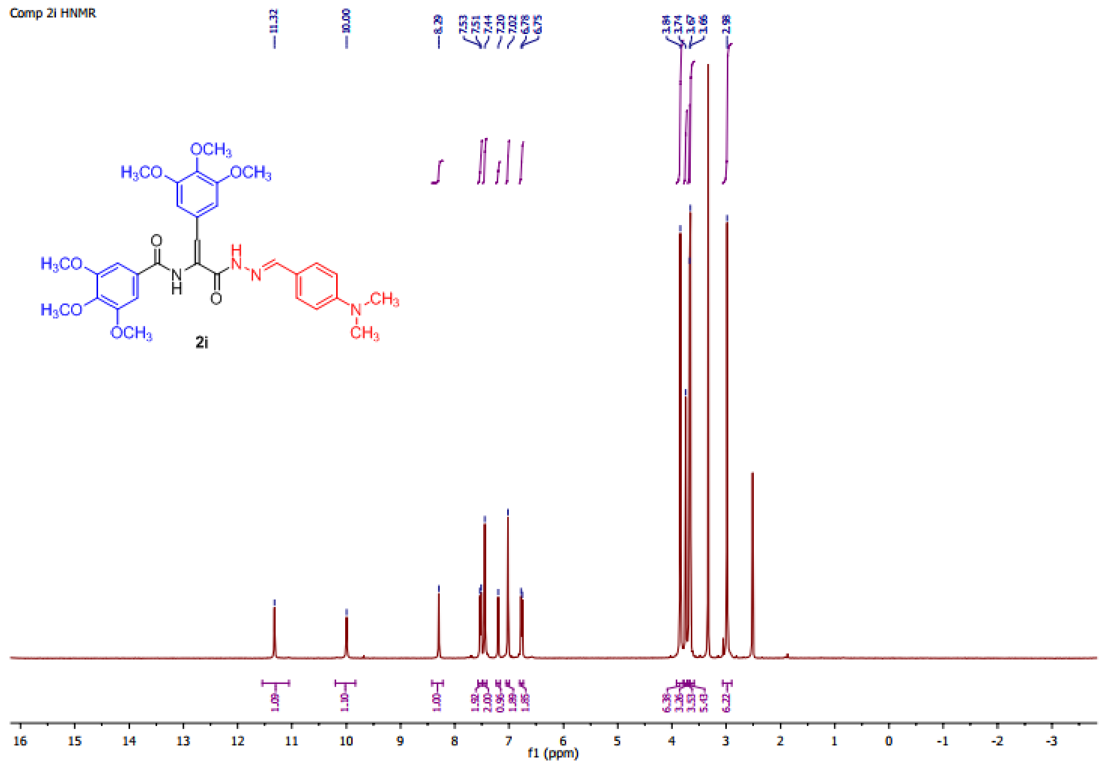

Figure S21: ^{1}H-NMR spectrum of compound **4i**

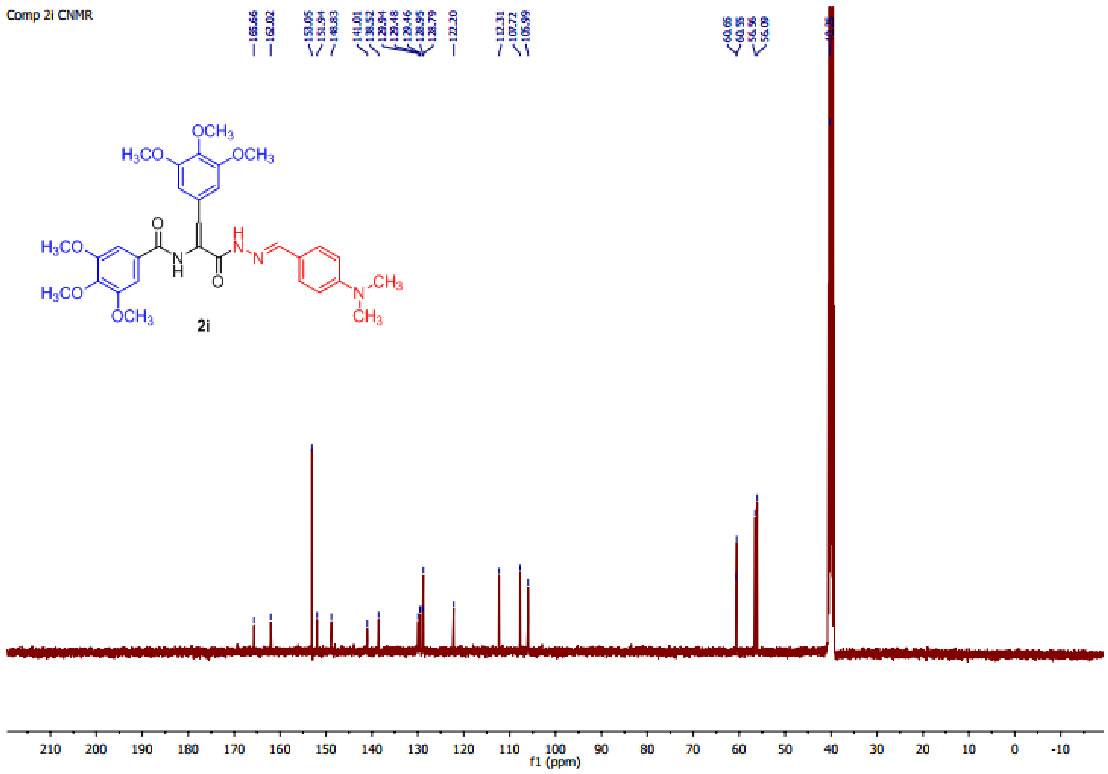

Figure S22: ^{13}C-NMR spectrum of compound **4i**

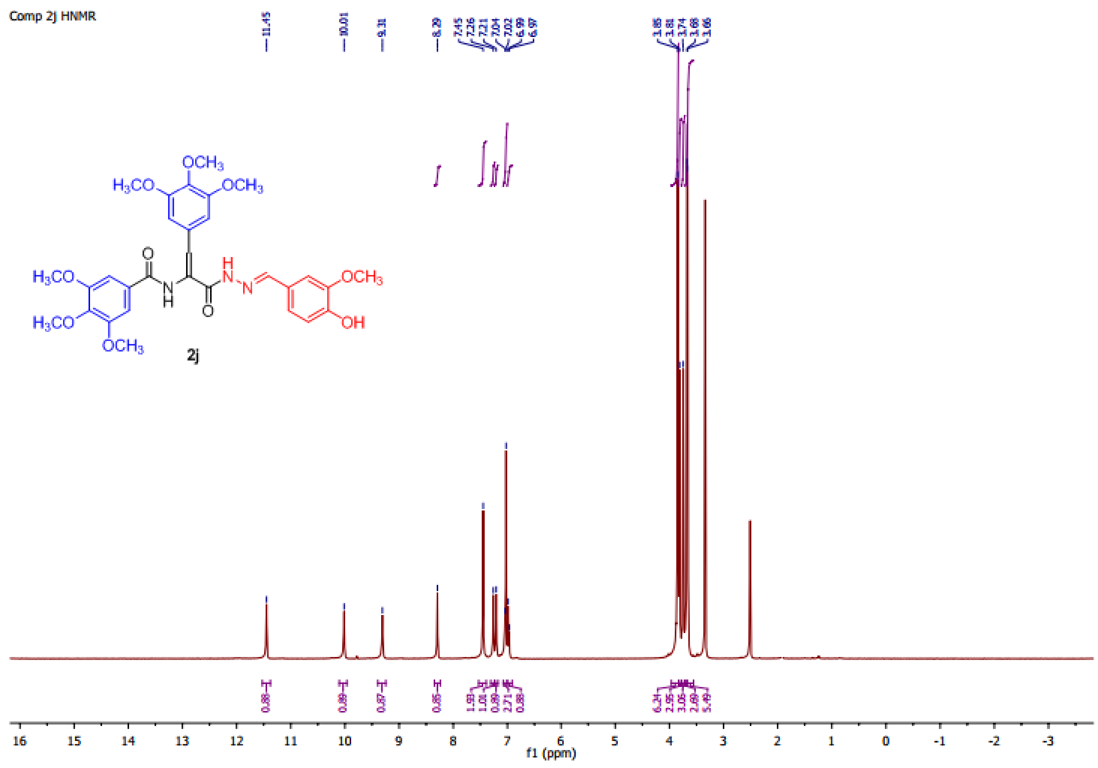

Figure S23: ^{1}H-NMR spectrum of compound **4j**

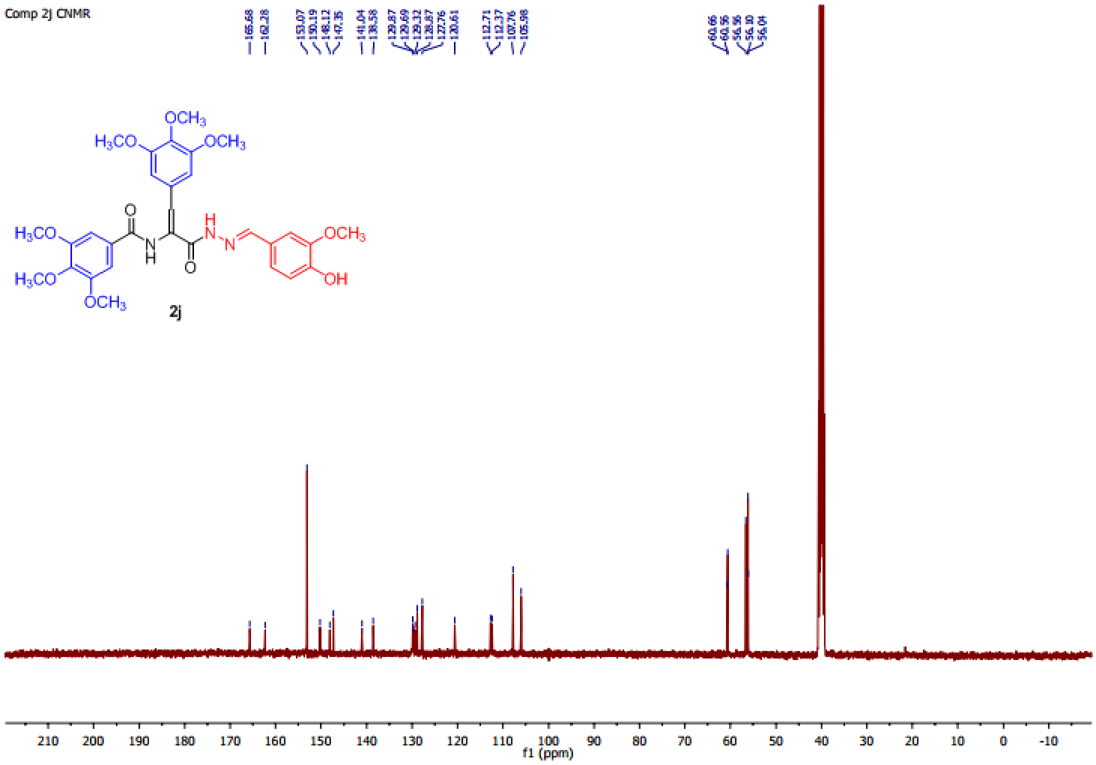

Figure S24: ^{13}C-NMR spectrum of compound **4j**

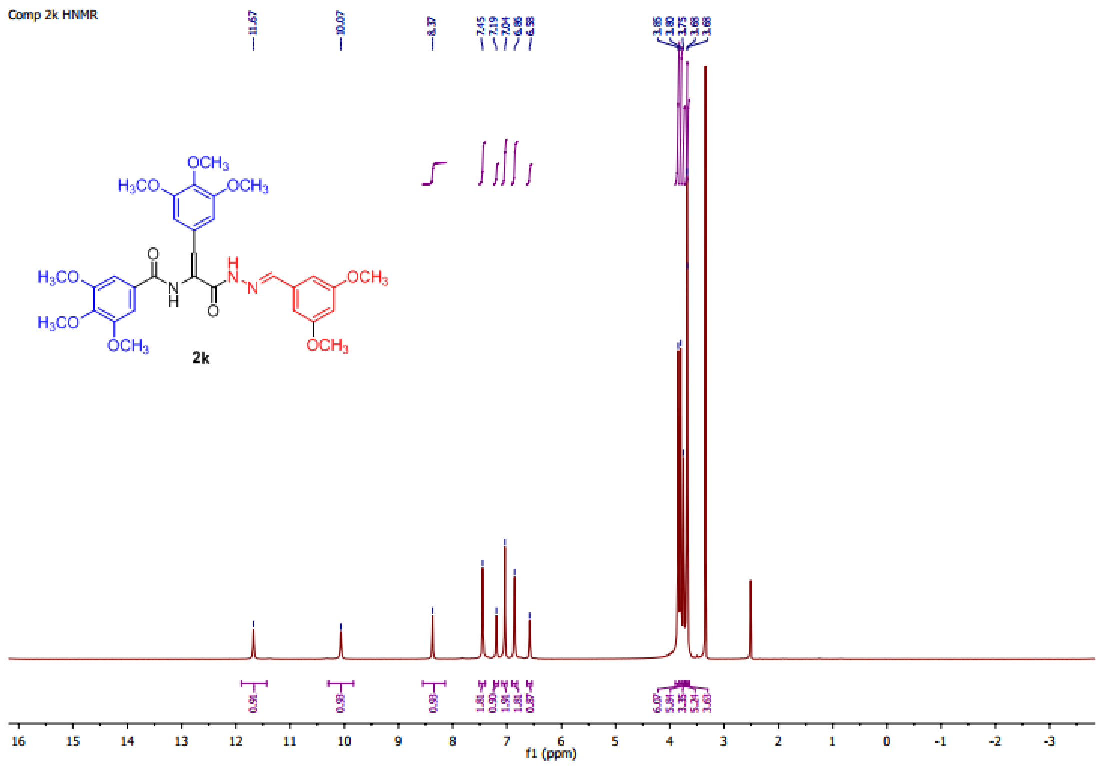

Figure S25: ^{1}H-NMR spectrum of compound **4k**

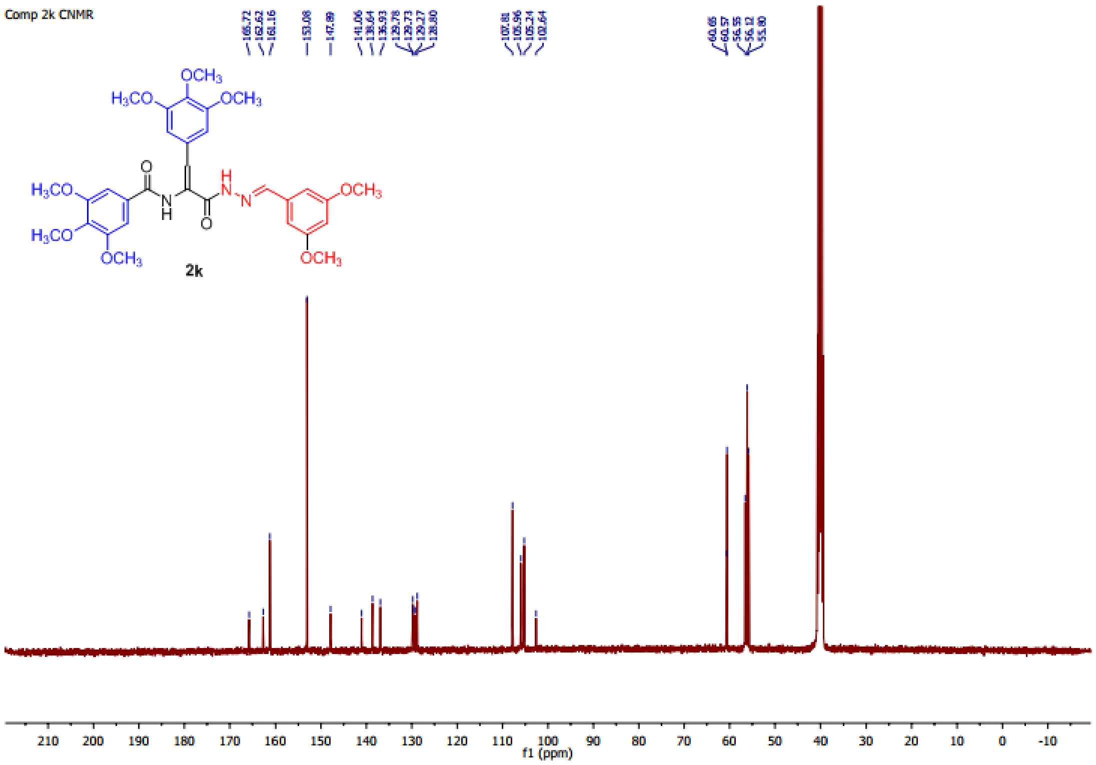

Figure S26: ^{13}C-NMR spectrum of compound **4k**

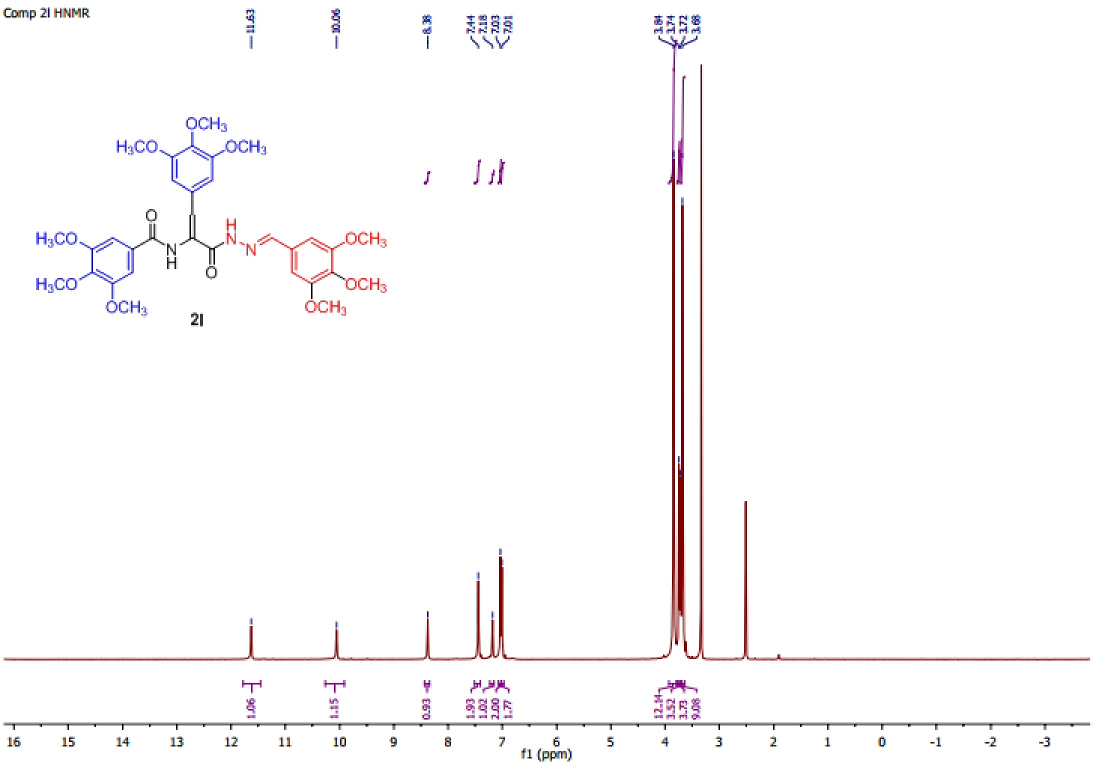

Figure S27: ^{1}H-NMR spectrum of compound **4l**

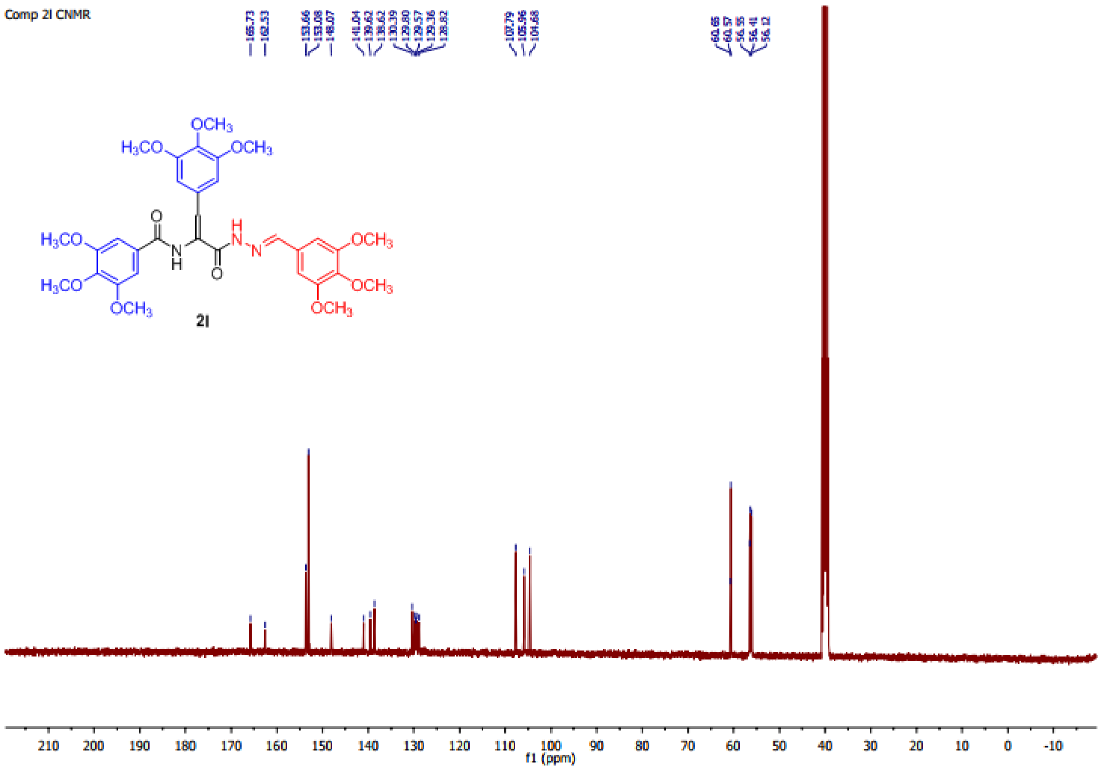

Figure S28: ^{13}C-NMR spectrum of compound **4l**

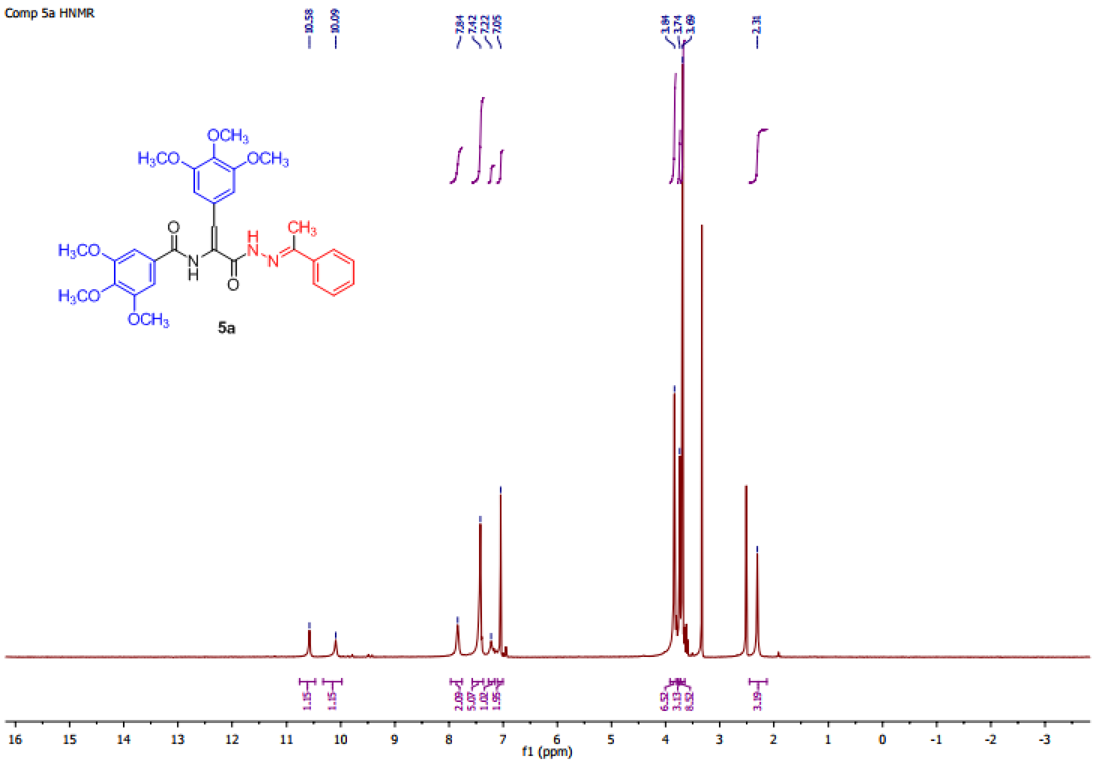

Figure S29: ^{1}H-NMR spectrum of compound **5a**

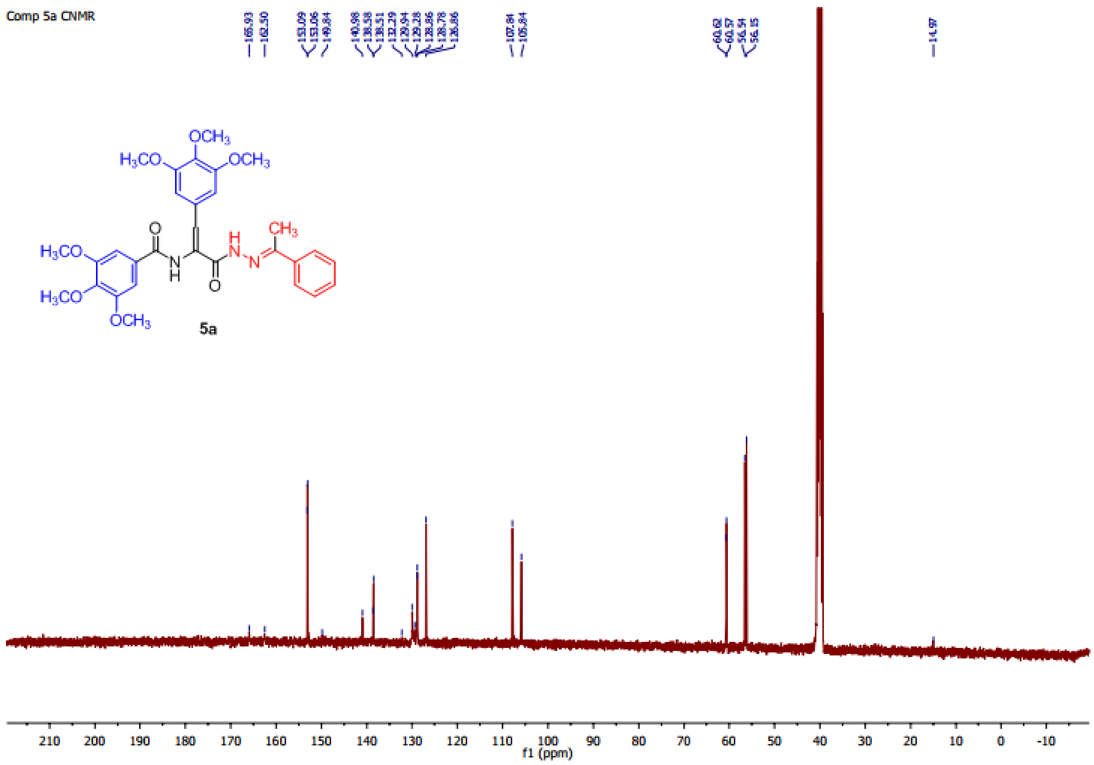

Figure S30: ^{13}C-NMR spectrum of compound **5a**

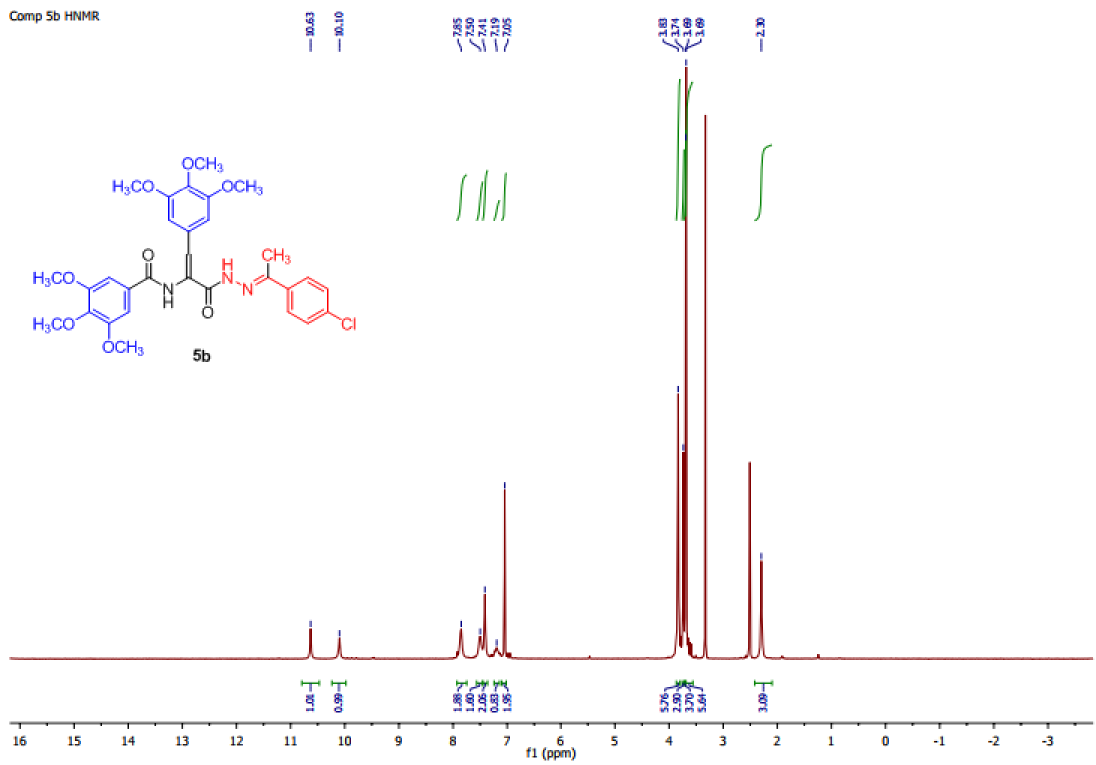

Figure S31: ^{1}H-NMR spectrum of compound **5b**

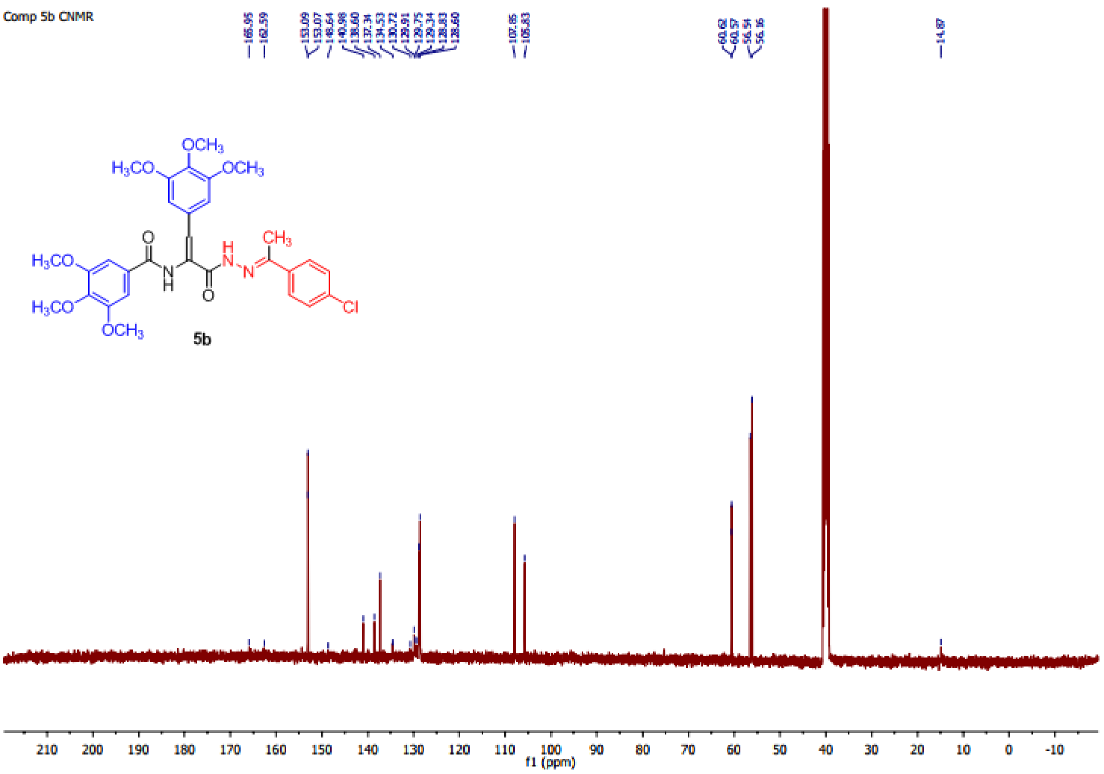

Figure S32: ^{13}C-NMR spectrum of compound **5b**

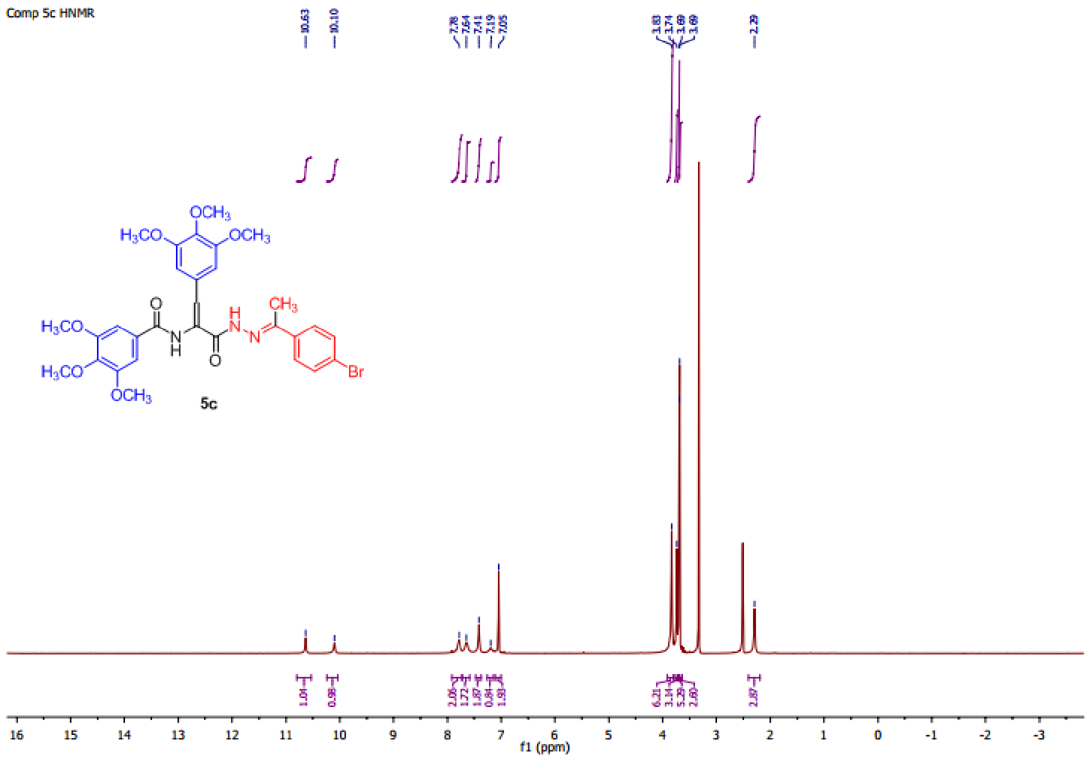

Figure S33: ^{1}H-NMR spectrum of compound **5c**

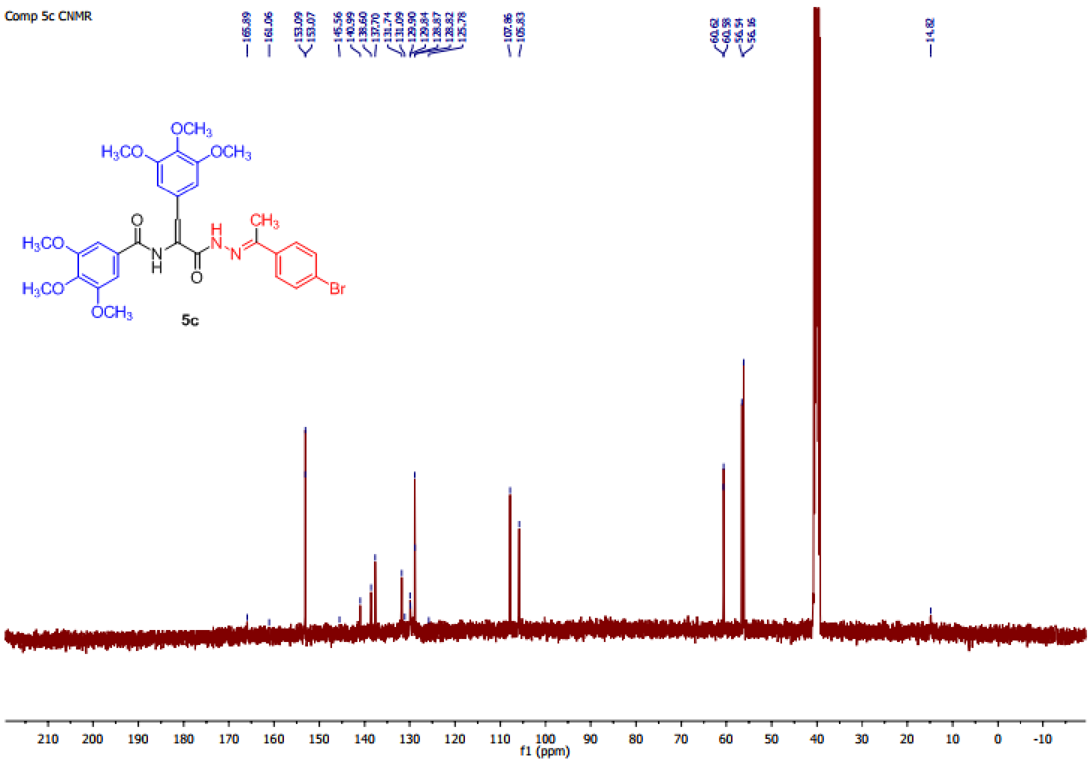

Figure S34: ^{13}C-NMR spectrum of compound **5c**

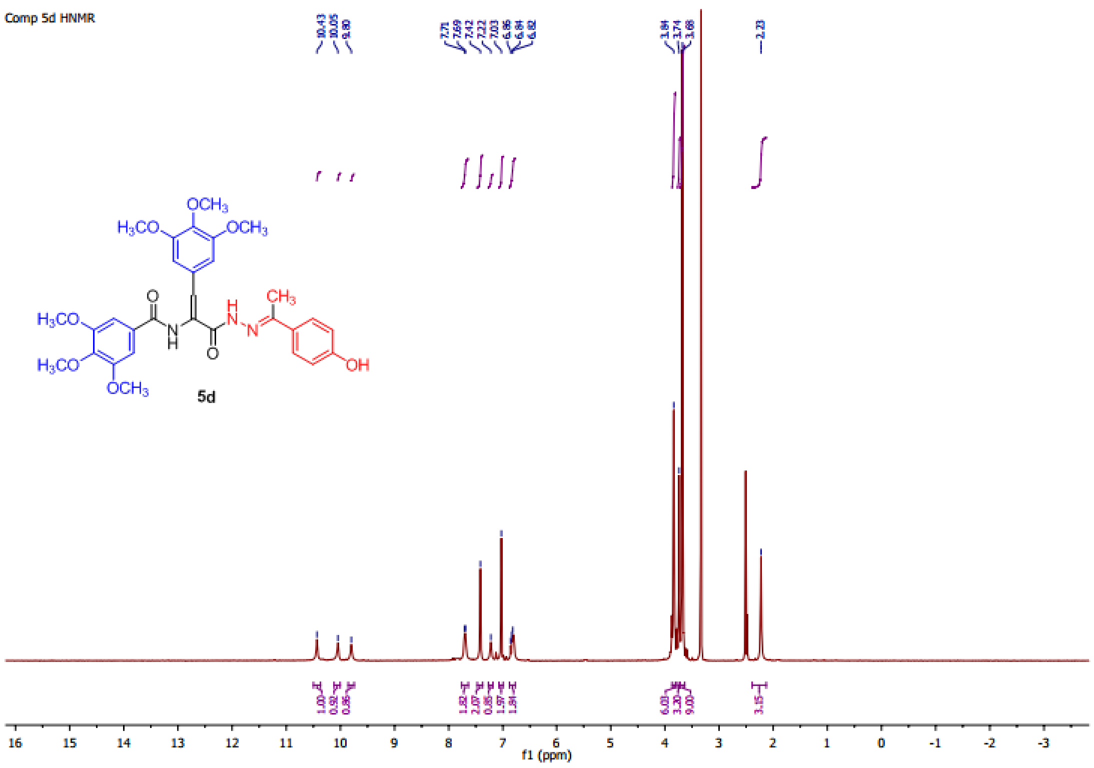

Figure S35: ^{1}H-NMR spectrum of compound **5d**

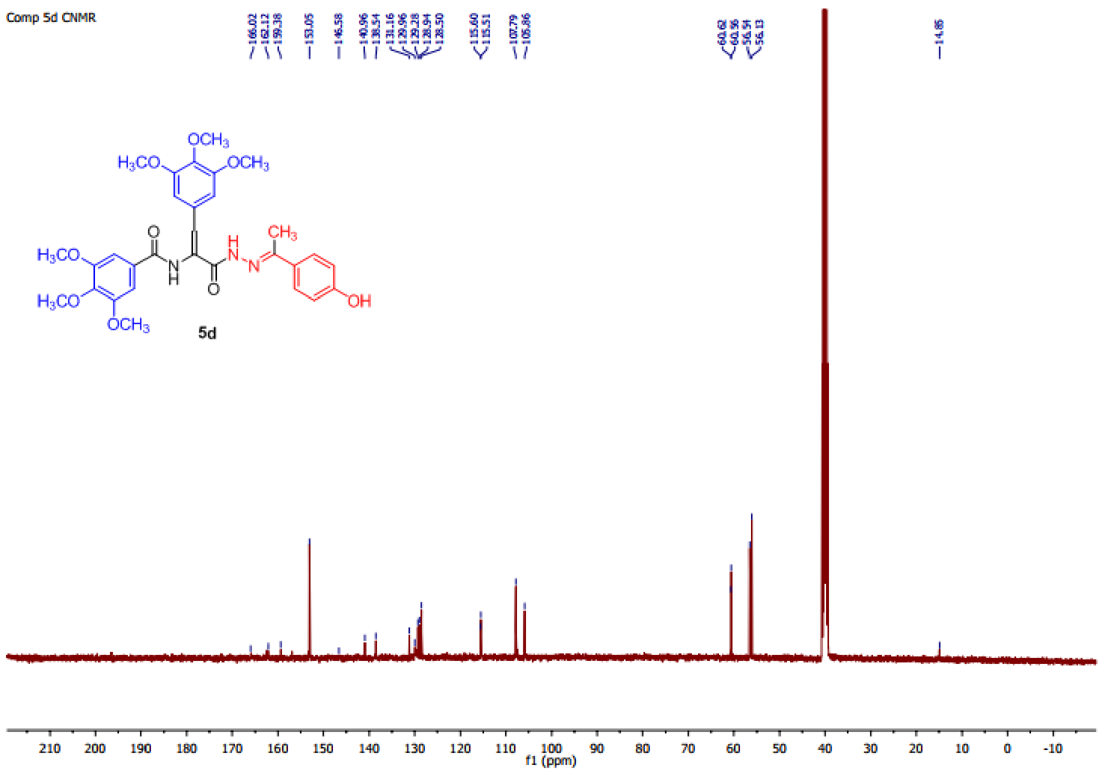

Figure S36: ^{13}C-NMR spectrum of compound **5d**

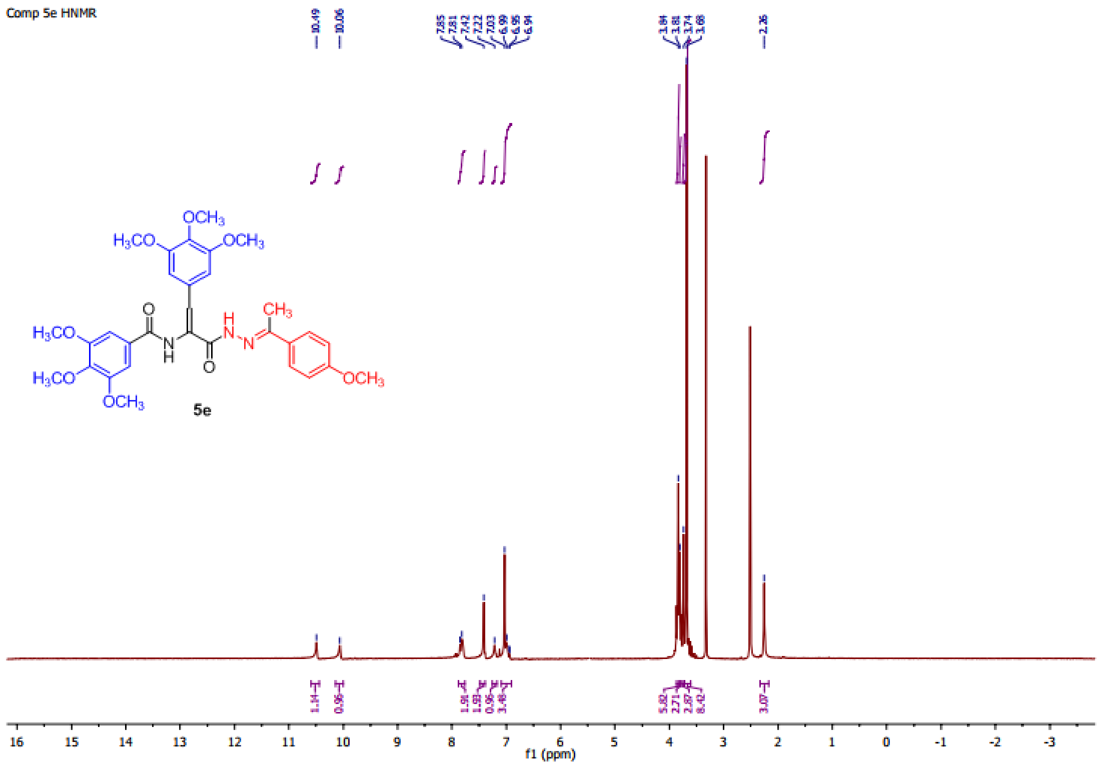

Figure S37: ^{1}H-NMR spectrum of compound **5e**

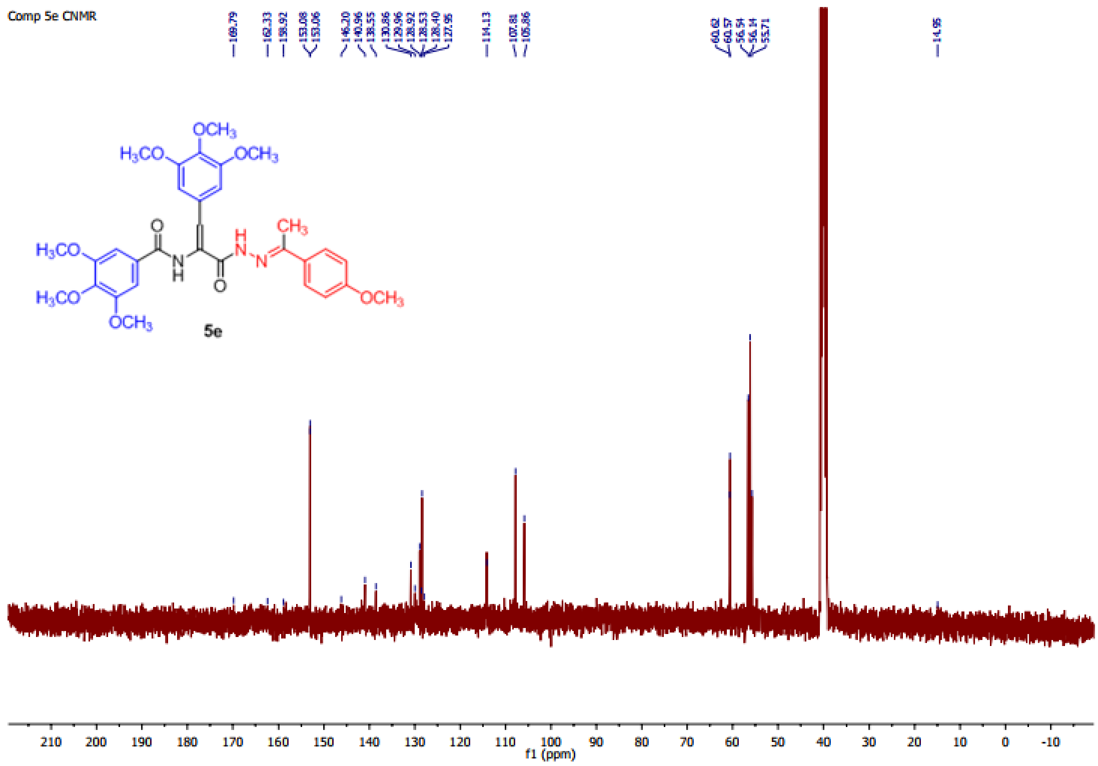

Figure S38: ^{13}C-NMR spectrum of compound **5e**

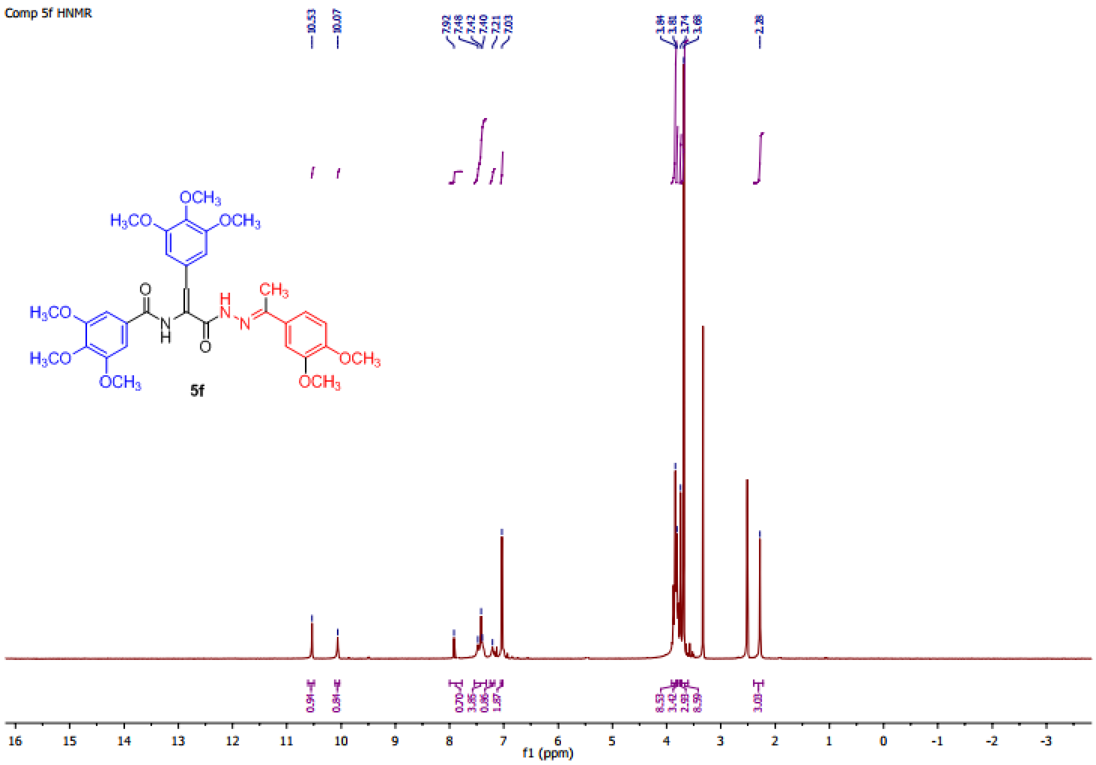

Figure S39: ^{1}H-NMR spectrum of compound **5f**

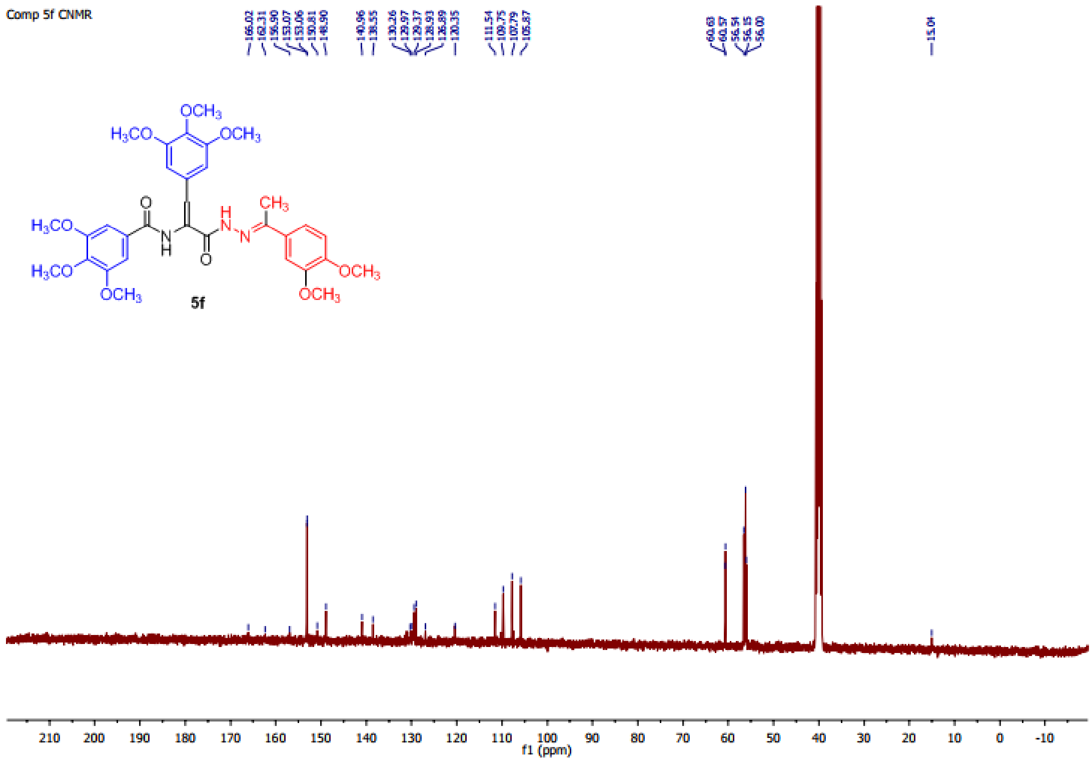

Figure S40: ^{13}C-NMR spectrum of compound **5f**

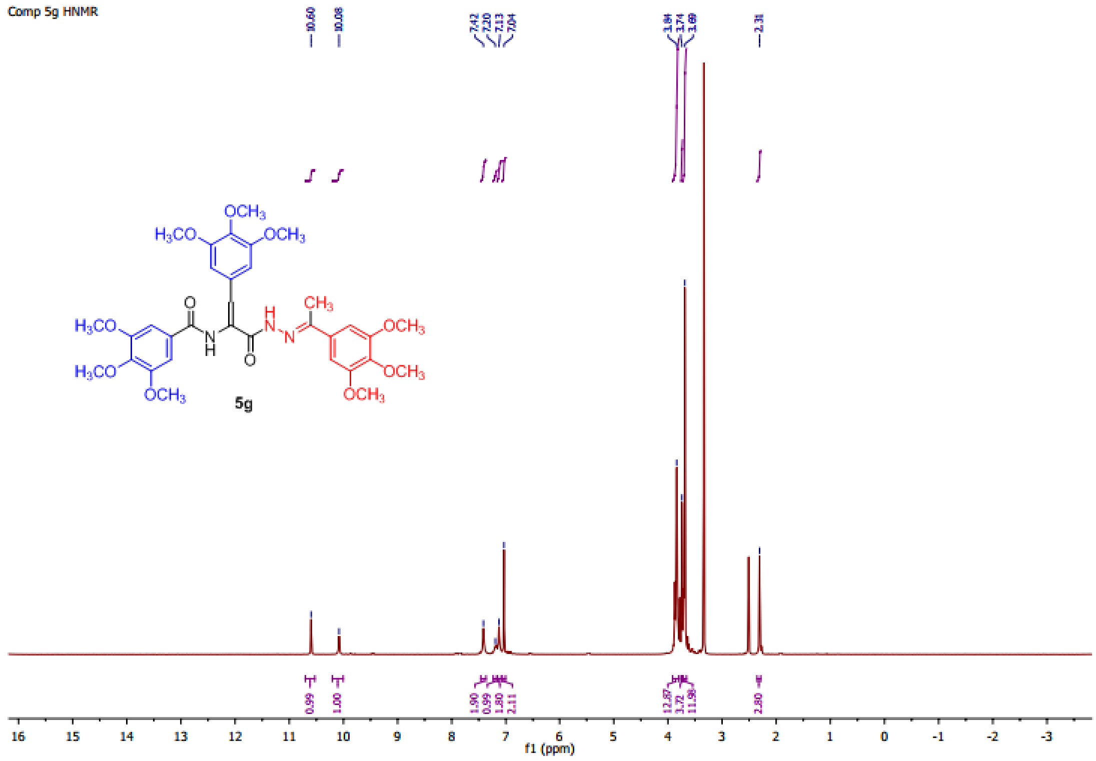

Figure S41: ^{1}H-NMR spectrum of compound **5g**

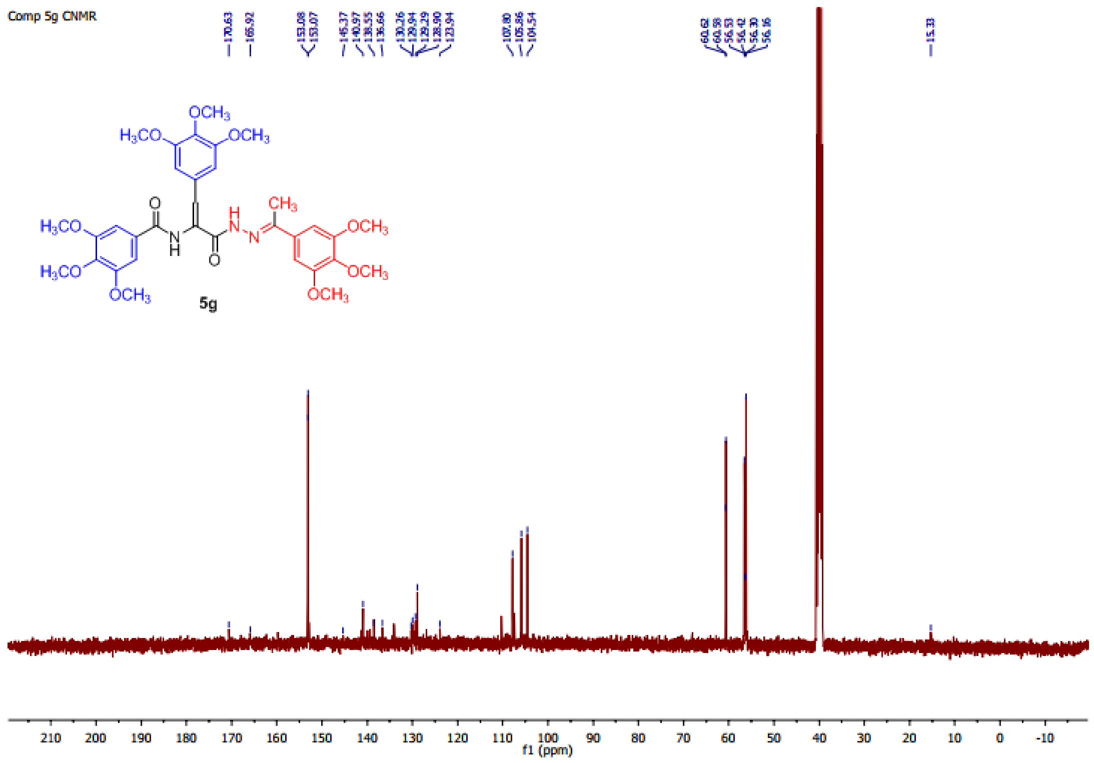

Figure S42: ^{13}C-NMR spectrum of compound **5g**
